# Supplementary material for: Randomized, double-blind, placebo-controlled trial of rapamycin in amyotrophic lateral sclerosis
Source: Nat Commun. 2023 Aug 17;14:4970. doi: 10.1038/s41467-023-40734-8 (PMC10435464; doi:10.1038/s41467-023-40734-8)
Supplement: Supplementary file 1 — Supplementary Information [file 41467_2023_40734_MOESM1_ESM.pdf]

SUPPLEMENTARY APPENDIX

This appendix has been provided by the authors to give readers additional information about their work.

Supplement to: Randomized, double.-blind, placebo-controlled trial of rapamycin in amyotrophic lateral sclerosis

TITLE

Randomized, double.-blind, placebo-controlled trial of rapamycin in amyotrophic lateral sclerosis

Authors

Jessica Mandrioli[1,2*], Roberto D'Amico[3,4§], Elisabetta Zucchi[2,5*], Sara De Biasi[4*], Federico Banchelli[3*], Ilaria Martinelli[2,6], Cecilia Simonini[2], Domenico Lo Tartaro[4], Roberto Vicini[3], Nicola Fini[2], Giulia Gianferrari[1,2], Marcello Pinti[7], Christian Lunetta[8,9], Francesca Gerardi[8], Claudia Tarlarini[8], Letizia Mazzini[10], Fabiola De Marchi[10], Ada Scognamiglio[10], Gianni Sorarù[11,12], Andrea Fortuna[11], Giuseppe Lauria[13], Eleonora Dalla Bella[13], Claudia Caponnetto[14], Giuseppe Meo[14], Adriano Chio[15], Andrea Calvo[15], Andrea Cossarizza[4,16§].

[1] Department of Biomedical, Metabolic and Neural Sciences, University of Modena and Reggio Emilia, Modena, Italy

[2]Department of Neurosciences, St. Agostino-Estense Hospital, Azienda Ospedaliero Universitaria di Modena, Modena, Italy

[3]Unit of Statistical and Methodological Support to Clinical Research, Azienda Ospedaliero-Universitaria, Modena, Italy.

[4]Department of Medical and Surgical Sciences for Children and Adults, University of Modena and Reggio Emilia, Modena, Italy.

[5]Neurosciences PhD Program, University of Modena and Reggio Emilia, Modena, Italy

[6]Clinical and Experimental Medicine PhD Program, University of Modena and Reggio Emilia, Modena, Italy

[7]Department of Life Sciences, University of Modena and Reggio Emilia, 41125 Modena, Italy.

[8]NEuroMuscular Omnicenter, Serena Onlus Foundation, Milan, Italy

[9]Istituto Maugeri IRCCS Milano, Milan, Italy

[10]ALS Centre, Neurologic Clinic, Maggiore della Carità University Hospital, Novara,Italy

[11]Department of Neurosciences, University of Padua, Padua, Italy

[12]Centro Regionale Specializzato Malattie del Motoneurone, Azienda Ospedale Università di Padova, Padua, Italy.

[13]3rd Neurology Unit and ALS Centre, IRCCS 'Carlo Besta' Neurological Institute, Milan, Italy

[14]Department of Neurosciences, Rehabilitation, Ophthalmology, Genetics, Mother and Child Disease, Ospedale Policlinico San Martino, Genova, Italy

[15]'Rita Levi Montalcini' Department of Neurosciences, ALS Centre, University of Turin and Azienda Ospedaliero Universitaria Città della Salute e della Scienza, Turin, Italy

[16]National Institute for Cardiovascular Research, via Irnerio 48, 40126 Bologna, Italy

*these authors contributed equally

§ these authors jointly supervised this work

Corresponding author:

Jessica Mandrioli

Department of Biomedical, Metabolic and Neural Sciences, University of Modena and Reggio Emilia, Modena, Italy; Department of Neurosciences, St. Agostino-Estense Hospital, Azienda Ospedaliero Universitaria di Modena, Modena, Italy

Via Pietro Giardini n. 1355, 41126 Modena, Italy

Tel. +390593961640 Fax +390593963775
Cell +393392633246
E-Mail jessica.mandrioli@unimore.it

Table of contents

1. Supplementary Introduction: Proposed Mechanisms of Action of Rapamycin in ALS 7

2. RAP-ALS Clinical Trial Sites and Site Investigators 9

3. Supplementary Methods... 11

Section 3.1. Biological outcome measures: laboratory methods... 11

Section 3.1.1 Blood collection and PBMC isolation 11

Section 3.1.2 Monocyte and lymphocytes immunophenotype by polychromatic flow cytometry 11

Section 3.1.3 Treg cells function after in vitro stimulation………………………………   21

Section 3.1.4 T helper differentiation………………………………………………….....22

Section 3.1.5 Neurofilament quantification 23

Section 3.1.6 Cytokine plasma levels quantification 23

Section 3.1.7 Monocytes’ isolation 23

Section 3.1.8 RNA extraction and gene expression analyses... 23

Section 3.2. Clinical outcome measures: methods.. 24

4. Supplementary Figures.. 25

Figure S1. Percentage of Tregs (CD4+, FoxP3+, CD127-) expressing LAG-3 measured in healthy controls and ALS patients before and after experimental treatment................................... 25

Figure S2. Th1 CD4+ T cells in healthy controls and in ALS patients at baseline and after treatment ……………………………………………………………………………...26

Figure S3. Individual rates of decline in ALSFRS-R total score of patients enrolled in RAP-ALS over the study. 28

Figure S4. Tracheostomy free survival (post hoc analysis, last observation set on 31st December 2021)... 28

Figure S5. Respiratory muscle function as assessed by FVC score decline from baseline to weeks 4, 8, 12, 18, 30, 42, and 54 across treatment arms.. 29

Figure S6: Box plots of mean rapamycin dosages from week 2 (second dosage from baseline) to week 12 (last dosage before treatment end). 31

Figure S7. CSF dosage of Sirolimus in 6 CSF samples of patients allocated to the three treatment arms. ... 32

5. Supplementary Tables ... 33

Table S1. Biological features of patients enrolled in the three trial arms at baseline ... 33

Table S2. Patients exhibiting a positive response (increase in Treg of at least 30%), comparing baseline and treatment end (week 18) between rapamycin and placebo arm. Per Protocol analysis. ... 34

Table S3. Changes in Treg cells during and after treatment across treatment arms ... 35

Table S4. Changes from baseline to week 18 in blood cells population across treatment arms ... 36

Table S5. Changes from baseline to week 18 in inflammasome and cytokines across treatment arms ... 42

Table S6. Changes from baseline to week 8-18-30-54 in serum pNfH in patients treated with rapamycin or placebo ... 44

Table S7. Changes from baseline to week 8-18-30-54 in serum NfL in in patients treated with rapamycin or placebo ... 45

Table S8. Changes from baseline to week 18 in CSF NfL and pNfH in patients treated with rapamycin or placebo ... 46

Table S9. Changes from baseline to week 18 and week 30 in the phosphorylation of S6 ribosomal protein across treatment arms ... 47

Table S10. Absolute changes from baseline to week 8-18-30-54 in ALSFRS-R total score in patients treated with rapamycin or placebo ... 48

Table S11. Tracheostomy free survival (post hoc analysis, last observation set on 31st December 2021) ... 49

Table S12. Respiratory function as measured by FVC (%) at different time points in patients treated with Rapamycin and placebo ... 50

Table S13. ALSAQ40 administered at different time points in patients treated with rapamycin and placebo ... 51

Table S14. Changes in clinical outcome measures during and after treatment across treatment arms54

Table S15. Correlations among clinical outcome measures ...55

Table S16 Individuals with Adverse Events across different treatment arms ... 56

Table S17. Adverse Events across different treatment arms 56

Table S18. Individuals with Serious Adverse Events across different treatment arms 56

Table S19. Analysis of drop out during the study treatment and follow up 57

Table S20. Trial Drug Adherence 57

6. References 58

7. Abbreviations list ……………………………………………………………………….61

8. Study protocol, version 2, 21st April 2017…………………………….………………64

1. Supplementary Introduction: Proposed Mechanisms of Action of Rapamycin in ALS

Despite extreme heterogeneity, the two pillars of ALS pathogenesis are represented by protein misfolding and dysfunction of the immune system, which present in the early stages in all patients and constantly evolve to the terminal phases[1].

The immune system in the CNS turns from protective to neurotoxic when immunoregulation failure leads to a state of sustained immune system activation, causing the secretion of pathogenic levels of proinflammatory mediators from reactive astrocytes and microglia that enhance MNs death and disease progression in ALS [2,3].

Peripheral immune function is dysregulated as well in ALS patients, with a reduced number and dysfunction of regulatory T lymphocytes (Tregs), consequent upregulation of self-sustaining inflammatory cytokines, enhanced peripheral immune cell migration into the brain[4,5]. Tregs number, which were proved to be less effective in suppressing responder T-lymphocytes, correlates with disease progression (low Tregs, faster disease progression)[6,7]. Enhancing effector Tregs in SOD1G93A transgenic mice slowed down disease progression and improved ALS mice lifespan[8].

The other primary drivers of this pathogenic process are protein misfolding and impairment of protein quality control system within motor neuronsplay a key role[9]. Autophagy is the primary cellular lysosomal degradative pathway involved in the degradation of damaged proteins and dysfunctional organelles; in ALS, misfolded protein aggregates in motor neurons are a histopathological hallmark, the majority of sporadic ALS showing TDP-43 aggregates and a minority of patients harboring SOD1 and FUS aggregates[10]. After this initial neuronal insult, neuroinflammation and impaired protein aggregates removal are related in a vitious cycle that auto-maintains itself[11] and that leads to inflammation-mediated neurodegeneration[12].

In facts, autophagy, the primary cellular lysosomal degradative pathway involved in the degradation of damaged proteins and dysfunctional organelles, is crucial non only for cell-autonomous clearance mechanisms, but limits detrimental and uncontrolled activation of inflammasomes[13], and impairments in this machinery induce abnormal activation of inflammasomes[14]. Aggregates may activate a cascade of events, that drives chronic inflammation via caspase-1-mediated proteolytic cleavage and secretion of proinflammatory cytokines, that further amplify inflammatory responses, resulting in chronic inflammation, tissue damage and cell death[15].

In summary, immune response and related autophagy play a major role in ALS pathogenesis and should be considered promising therapeutic targets because their dysregulation is seen in all patients, independently of the genetic background.

Mechanistic Target of Rapamycin (mTOR) refers to two protein complexes, mTOR Complex 1 and 2 (mTORC1 and mTORC2), that function as master switches in the integration of cell's crucial signals. mTORC1 supports protein synthesis via translation regulation and controls both protein and organelle degradation through autophagy[16].

Rapamycin, a drug used to prevent renal transplantation rejection, inhibits mTORC1 and in this way exerts different actions on key process underlying ALS pathogenesis.

Rapamycin enhances the autophagic degradation of various aggregate-prone proteins with subsequent reduction of their toxicity in cellular or animal models not only in ALS, but also in other neurodegenerative diseases e.g. Huntington disease [17-20]. Autophagy enhancement by rapamycin is mediated by the unc-51-like kinase 1 complex and the formation of autophagosome from the phagophore.[19]

Besides, rapamycin increases the expression Beclin-1 and LC3-II/LC3-I that are required for the initiation of autophagosome formation, and reduces the expression of P62, promoting autophagosome formation[21].

Rapamycin induction of autophagy has been demonstrated to be time-dependent and concentration-dependent in iPSCs, where it determined the largest effect at a high concentration of 200 nM[22].

As far as ALS is concerned, rapamycin administration exerted a beneficial effect in cell lines characterized by TDP-43 and FUS pathology[23-25] and in several ALS animal models, namely TDP-43 mouse, SQSTM1 knock-down zebrafish and in Drosophila with VAPB mutation, improving motor and cognitive phenotype[26-30]. In human stem cell-derived neurons and astrocytes with mutant TDP43, autophagy enhancement by rapamycin improved TDP43 clearance and localization and enhanced survival[18.]

Besides autophagy, mTOR inhibitors maintain homeostasis of T-cells by preventing them from engaging alternative paths. Indeed, naïve CD4+ T-cells can develop into TH1, TH2 or TH17 effectors using pathways promoted by mTOR, that on the contrary inhibits Tregs induction.

In particular, mTOR phosphorylation and activation of lineage-specific transcription factors drives T cell differentiation that leads to mTOR-mediated control of cytotoxic T lymphocytes trafficking. mTOR inhibitors obstacle cytotoxic T lymphocytes ability to migrate to peripheral tissues for optimal function[31]. Furthermore mTOR upregulate the senescence-associated secretory phenotype (SASP),[32] that is a specific set of cytokines (including IL-1, IL-6, and IL-8), proteases, and growth factors secreted by metabolically active senescent cells which create a pro-inflammatory microenvironment[33]. mTOR upregulates the SASP and NF-κB and IL-1R provide a positive feedback loop, stimulating the transcription of multiple genes encoding inflammatory cytokines[34]. Rapamycin suppresses mTOR and therefore IL-6, IL-8 and several pro-inflammatory cytokines, chemokines and growth factors secretion[35]. Previous studies demonstrated that mTOR inhibition controls immunosenescence,

decreases CD4 and CD8 T cells number with PD-1 expression and keep tissue homeostasis by reducing chronic, low-grade inflammation which characterize aging tissues[36].
Several trials tried to increase or stimulate Tregs in ALS in an attempt to slow down disease progression. In a phase I trial, infusion of ex vivo expanded autologous Tregs was found safe and well-tolerated in ALS patients, that showed a slowed disease progression[37], and reduced oxidative stress and circulating pro-inflammatory acute phase proteins[38].
Treatment with IL-2/IL-2-antibody complex increased Tregs in mSOD1 mice and slowed down disease progression as well[39].
Inhibition of mTORC1 by rapamycin expands Tregs and enhances autophagy, also in ALS cellular and animal models where it facilitates TDP43 clearance and regulates immune responses[18]. Rapamycin has never been tested in ALS.
Since these promising mechanisms of action are relevant for ALS pathogenesis, a phase II clinical trial of rapamycin in ALS patients was conceived.

2. RAP-ALS Clinical Trial Sites and Site Investigators

Trial Sites and Locations	Principal Investigator	Sub-investigators and collaborators
**Modena**		
ALS Center, Azienda Ospedaliero Universitaria di Modena, Modena, Italy	Jessica Mandrioli, M.D.	Nicola Fini, M.D.<br>Ilaria Martinelli, M.D.<br>Elisabetta Zucchi, M.D.<br>Giulia Gianferrari, M.D.<br>Cecilia Simonini, BSc<br>Roberta Bedin, BSc<br>Annalisa Gessani, S.L.P.<br>Francesca Prompicai, R.N.<br>Silvia Parisi, R.N.<br>Daniela Gallesi, M.D.
**Novara**		
ALS Center, Maggiore della Carità Hospital, University of Piemonte Orientale, Novara, Italy.	Letizia Mazzini, M.D.	Fabiola De Marchi, M.D.<br>Ada Scognamiglio, M.D.<br>Paola Odoli
**Turin**		
ALS Centre, Azienda Ospedaliero Universitaria Città della Salute e della Scienza, University of Turin, Turin, Italy	Adriano Chiò, M.D.	Andrea Calvo, Ph.D.<br>Cristina Moglia, Ph.D.
**Genoa**		
Neurological Clinic, IRCCS Ospedale Policlinico San Martino, Genoa, Italy.	Claudia Caponnetto, M.D.	Giuseppe Meo, M.D.<br>Corrado Cabona, M.D.
**Padua**		
Centro Regionale Specializzato Malattie del Motoneurone, Azienda Ospedale Università di Padova, Padua, Italy.	Gianni Sorarù, Ph.D.	Andrea Fortuna, M.D.
**Milan**		
NEuroMuscular Omnicentre (NEMO), Fondazione Serena Onlus, Milan, Italy.	Christian Lunetta, M.D.	Valeria Sansone, M.D.<br>Francesca Gerardi, BSc.<br>Claudia Tarlarini, BSc.
IRCCS Istituto Neurologico Carlo Besta di Milano, Milan, Italy	Eleonora Dalla Bella, M.D.	Giuseppe Lauria Pinter, M.D.<br>Enrica Bersano, M.D.<br>Raffaella Lombardi, BSc.

RAP-ALS investigators group includes:

ALS Center, Azienda Ospedaliero Universitaria di Modena, University of Modena and Reggio Emilia, Modena, Italy: Jessica Mandrioli, Nicola Fini, Ilaria Martinelli, Elisabetta Zucchi, Giulia Gianferrari, Chiara Biral, Cecilia Simonini, Roberta Bedin, Annalisa Gessani, Francesca Prompicai, Silvia Parisi, Daniela Gallesi

Unit of Statistical and Methodological Support to Clinical Research, Azienda Ospedaliero Universitaria di Modena, University of Modena and Reggio Emilia, Modena, Italy: Roberto D'Amico, Federico Banchelli, Roberto Vicini, Riccardo Cuoghi Costantini

Laboratory of Immunology, Department of Medical and Surgical Sciences for Children and Adults, University of Modena and Reggio Emilia, Modena, Italy: Andrea Cossarizza, Sara de Biasi, Domenico Lo Tartaro, Anita Neroni, Marcello Pinti

ALS Center, Maggiore della Carità Hospital, University of Piemonte Orientale, Novara, Italy: Letizia Mazzini, Ada Scognamiglio, Fabiola De Marchi, Paola Odoli

ALS Centre, Azienda Ospedaliero Universitaria Città della Salute e della Scienza, University of Turin, Turin, Italy: Adriano Chiò, Adrea Calvo, Cristina Moglia

IRCCS Istituto Neurologico Carlo Besta di Milano, Milan, Italy: Giuseppe Lauria, Eleonora Dalla Bella, Raffaella Lombardi, Enrica Bersano

NEuroMuscular Omnicentre (NEMO), Fondazione Serena Onlus, Milan, Italy: Christian Lunetta, Claudia Tarlarini, Francesca Gerardi, Valeria Sansone

Neurological Clinic, IRCCS Ospedale Policlinico San Martino, Genoa, Italy: Claudia Caponnetto, Giuseppe Meo, Corrado Cabona

Centro Regionale Specializzato Malattie del Motoneurone, Azienda Ospedale Università di Padova, Padua, Italy: Gianni Sorarù, Andrea Fortuna

3. Supplementary Methods

Section 3.1. Biological outcome measures: laboratory methods

Section 3.1.1 Blood collection and PBMC isolation

Thirty milliliters of blood were collected in vacuette containing EDTA and immediately processed according to biosafety rules. Isolation of PBMC was performed by using Ficoll–Paque according to standard procedures[40]. PBMC were used as freshly isolated for monocytes, NK cells and T cells immunophenotyping and detection of S6 Ribosomal Protein phosphorilation or stored in liquid nitrogen in FBS added with 10% DMSO for B cell analysis. Plasma was collected, centrifuged at 2120 g, and stored at−80°C until use.

Section 3.1.2 Monocyte and lymphocytes immunophenotype by polychromatic flow cytometry

Freshly isolated PBMC were washed and stained with commercially available monoclonal antibodies (mAb) directly conjugated with different fluorochromes. To fully dissect monocytes, NK cells and T cells we used three different flow cytometry panels. For monocyte phenotype: beside viability marker (AQUA, Live Dead, ThermoFisher, Eugene, OR) anti-CD14-APC, -CD16-AF488, HLA-DR-Pe-Cy7, and chemokine receptors CCR2-BV421, CXCR4-PE, CCR5-BV605 (all from Biolegend, San Diego, CA).

Classical monocytes were defined as CD14+, CD16+, intermediate monocytes were defined as CD14+,CD16bright, non-classical monocytes were defined as CD14-CD16bright. Median fluorescent intensity (MFI) of chemokine receptors was measured in all monocyte subpopulations.

For the immunophenotype of NK cells the following markers were used, beside viability marker (AQUA Live Dead, ThermoFisher, Eugene, OR): anti-CD16-BV421, -CD56-PE-Cy7, -CD158a-PE, -CD158b-FITC, HLA-DR-AF700, CD62L-BV605, CD57-APC, -CD3-PE-Dazzle-594, -CD4-PE-Dazzle-594-PE-Dazzle-594, -CD14-PE-Dazzle-594, -CD19-PE-Dazzle-594.

For T regulatory cells and T cells phenotype the following markers, beside viability marker (AQUA Live Dead) were used: anti-CD3-Pe-Cy5, -CD4-AF700, CD8-APC-Cy7, CD25-PE, CD127-APC-Cy7, HLA-DR-PE-Cy7, CXCR3-BV421, CD38-BV605, PD-1-BV605, CD39-BV421. Cells were fixed and perm using Human FoxP3 Buffer Set (BD Bioscience, San José, CA) and the stained with anti-FoxP3-PE (BD).

Finally, functional analysis on freshly isolated PBMC after *in vitro* stimulation for 16 hours with anti-CD3 plus -CD28 (1ug/ml, each, Miltenyi, Germany) was performed to evaluate the metabolic mTOR function by the evaluation of the phosphorylation of S6 Ribosomal Protein, a protein phosphorylated by mTOR. Cells were stained with viability marker (AQUA Live Dead, ThermoFisher), anti- CD3-PE-Cy5, -CD4-AF700, -CD8-APC-Cy7, -CD127-APC-Cy7, -CCR7-FITC, -CD45RA-PE-Cy7, -CD25-BV605. Cells were washed and then fixed and perm with Intracellular Fixation & Permeabilization Buffer Set (eBioscience, San Diego, CA, USA). Finally, cells were stained with PE-conjugated S6 Ribosomal Protein (Ser235/Ser236) (eBioscience, San Diego, CA, USA) to evaluate the activation of mTOR. Cells were acquired on Attune NxT acoustic flow cytometer and a minimum of 500 000 cells was acquired.

B-cell immunophenotype was perfomed by using DuraClone IM B tubes (Beckman Coulter, Hialeah, FL). Thawed PBMC were stained with viability marker Promokine IR-840 (PromoCell GmbH, Heidelberg, Germany) for 20 min at room temperature in PBS. One million PBMC were washed with FACSbuffer and stained with DuraClone IM B cells containing the following lyophilized directly conjugated mAbs: IgD-FITC, CD21-PE, CD19-ECD, CD27-PC7, CD24-APC, CD38-AF750, IgM-PB, CD45-KrO. Cells were washed with FACS buffer and acquired at CytoflexLX flow cytometer (Beckman Coulter, Hialeah, FL). A minimum of 500 000 cells was acquired on a CytoFLEX LX flow cytometer (Beckman Coulter) according to the state-of-the-art methodology[41].

Flow cytometry data were compensated in FlowJo by using single stained controls (BD Compbeads incubated with fluorochrome-conjugated antibodies) and gates were put according to fluorescence minus one (FMO) controls.

Reagents used in the flow cytometry panel are presented in the following tables (3.3.2 from A to D)

Gating strategies are presented in the following figures (3.3.2 from A to D)

Table 3.1.2A mAbs used for Monocyte phenotype

Specifity	Fluorocrome	Manufacturer	Cat.#	Titer (uL)
**Viability**	AQUA	Thermo-Fisher	L34966	1.25
**CD14**	APC	Biolegend	367118	0.3
**CD16**	AF488	Biolegend	302019	0.3
**HLA-DR**	PE-Cy7	Biolegend	307616	0.3
**CCR2**	BV605	Biolegend	357214	0.3
**CCR5**	BV421	Biolegend	306506	0.3
**CXCR4**	PE	Biolegend	359118	0.3

Table 3.1.2B mAbs used for NK cells phenotype

Specifity	Fluorocrome	Manufacturer	Cat.#	Titer (uL)
**Viability**	AQUA	Thermo-Fisher	L34966	1.25
**CD3**	PE-DAZZLE 594	Biolegend	300450	0.3
**CD4**	PE-DAZZLE 594	Biolegend	300548	0.3
**CD14**	PE-DAZZLE 594	Biolegend	325634	0.3
**CD19**	PE-DAZZLE 594	Biolegend	302252	0.3
**CD16**	BV421	Biolegend	302038	0.3
**CD56**	PE-Cy7	Biolegend	304628	0.3
**CD8**	APC-Cy7	Biolegend	301016	0.3
**CD62L**	BV605	Biolegend	304834	0.3
**CD158A**	PE	Biolegend	339506	0.3
**CD158B**	FITC	Biolegend	312604	0.3
**HLA-DR**	AF700	Biolegend	327014	0.3
**CD57**	APC	Biolegend	359614	0.3

Table 3.1.2C mAbs used for T cells phenotype

Specifity	Fluorocrome	Manufacturer	Cat.#	Titer (uL)
**Viability**	AQUA	Thermo-Fisher	L34966	1.25
**CD3**	PE-Cy5	Biolegend	300410	0.6
**CD4**	AF700	Biolegend	300526	0.6
**CD8**	APC-Cy7	Biolegend	301016	0.6
**CD127**	APC-Cy7	Biolegend	351316	0.6
**CD25**	PE	Biolegend	302606	3.75
**CXCR3**	BV421	Biolegend	353716	20
**Foxp3**	AF488	BD	560047	1.25
**CD38**	BV605	Biolegend	303532	0.6
**HLA-DR**	PE-Cy7	Biolegend	307616	0.6
**PD1**	BV605	Biolegend	329924	2.5
**CD39**	BV421	Biolegend	328212	0.6

Table 3.1.2D mAbs used for pS6 phosphorylation in Treg cells

**Specifity**	**Fluorocrome**	**Manufacturer**	**Cat.#**	Titer (uL)
**Viability**	AQUA	Thermo-Fisher	L34966	1.25
**CD3**	PE-Cy5	Biolegend	300410	0.6
**CD4**	AF700	Biolegend	300526	0.6
**CD8**	APC-Cy7	Biolegend	301016	0.6
**CD127**	APC-Cy7	Biolegend	351316	0.6
**CCR7**	FITC	Biolegend	353208	0.6
**CD45RA**	PE-Cy7	Biolegend	304108	1.25
**CD25**	BV605	Biolegend	302632	1.25
**pS6**	PE	eBioscience	25-9007-42	3.75

Figure 3.1.2A. Treg cells quantification. Lymphocytes have been identified according to physical parameters (FSC-H and SSC-H), doublets were removed according to FSC-A vs FSC-H plot, living CD3+ T cells were selected and in this population those cells expressing CD4 were selected. Treg cells were identified as FoxP3+, CD25++, CD127- cells within CD4+ T cells.

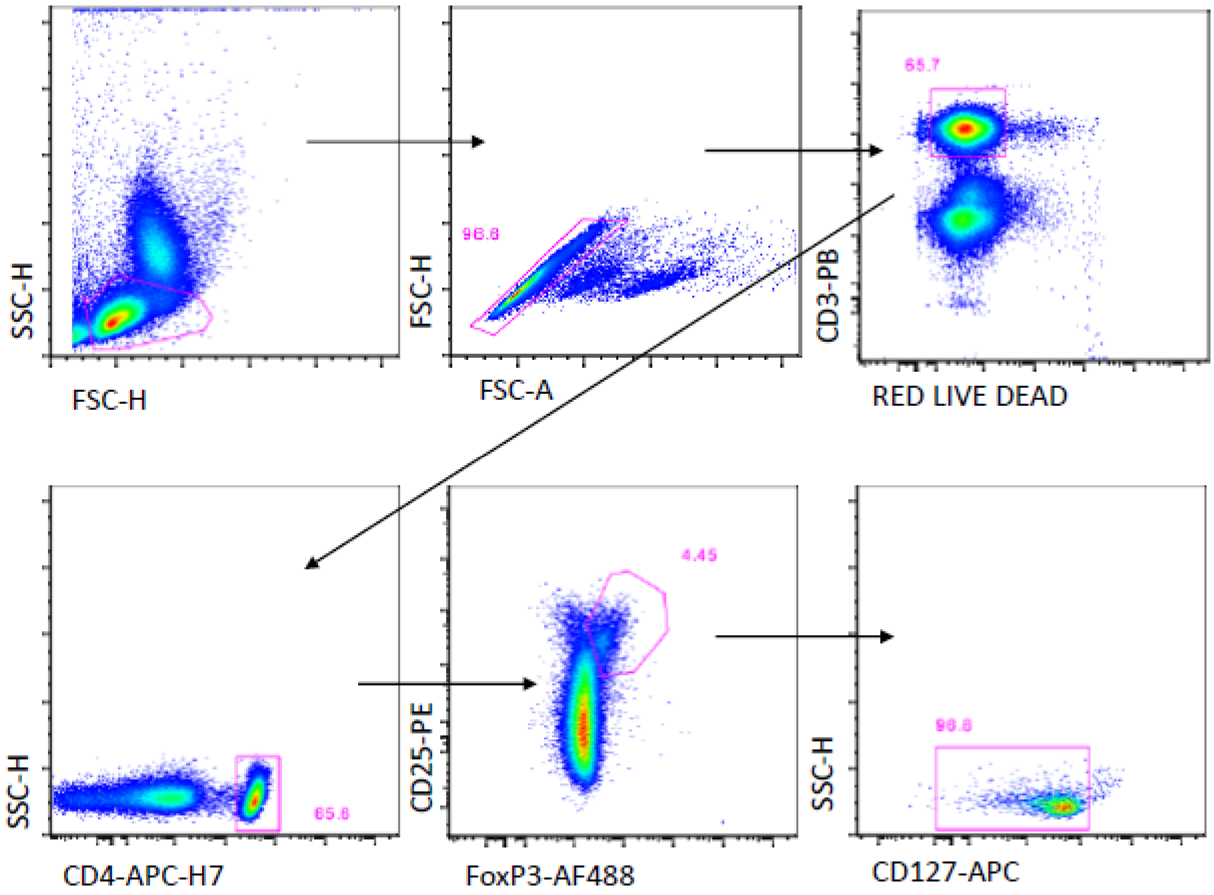

Figure 3.1.2.B. T cell Activation panel.
Lymphocytes have been identified according to physical parameters (FSC-H and SSC-H), doublets were removed according to FSC-A vs FSC-H plot, living CD3+ T cells were selected and in this population those cells expressing CD4 and CD8 were selected. Within CD4+ T cells and CD8+ T cells the expression of CD38, HLA-DR and CXCR3 was analysed. Activated cells were defined those expressing both CD38 and HLA-DR. Cells expressing CXCR3, a homing receptor, were also analyzed. Activation status and homing properties have been investigated also in Treg cells.

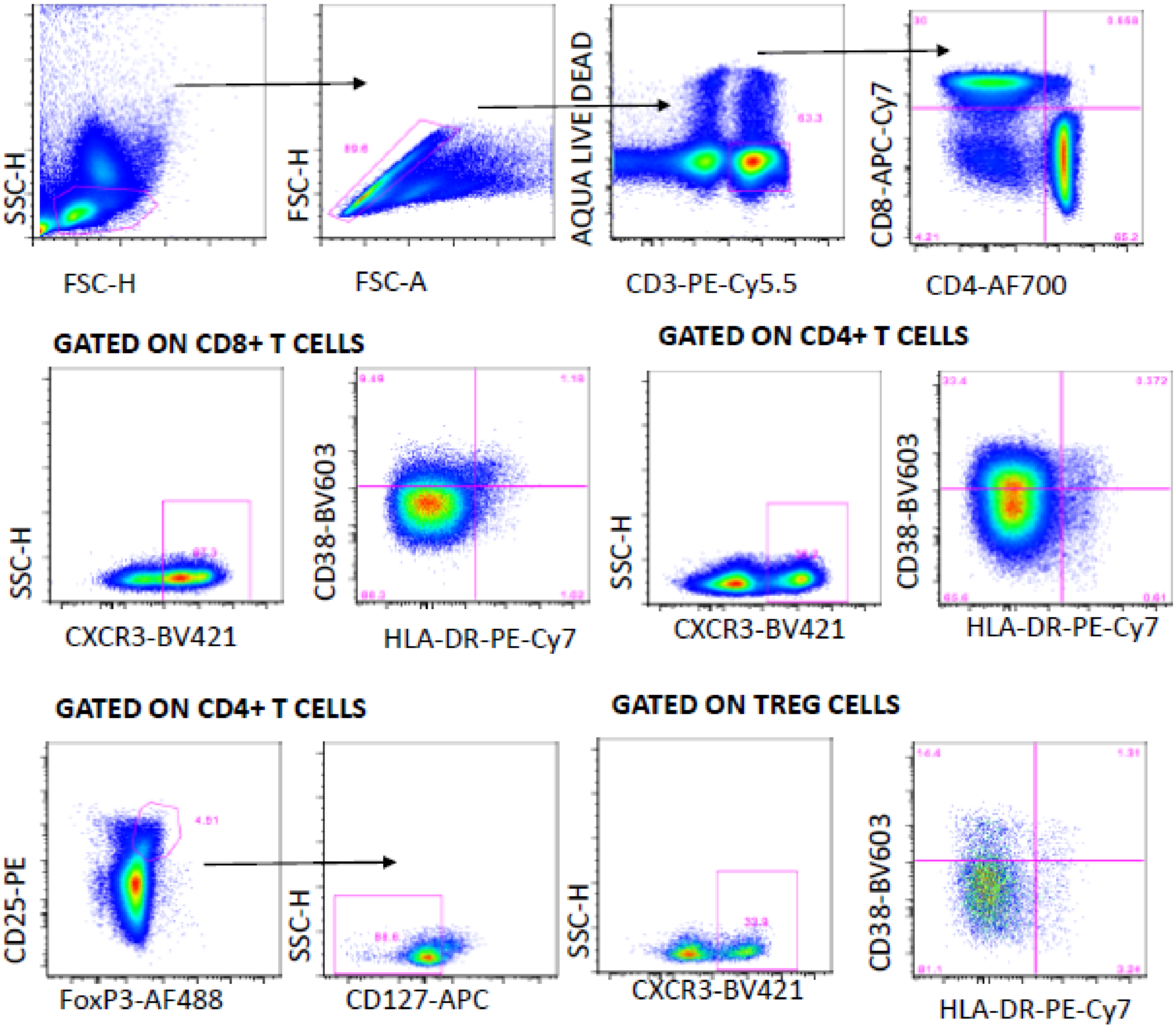

Figure 3.1.2.C. T cell Metabolic/Exhaustion panel. Lymphocytes have been identified according to physical parameters (FSC-H and SSC-H), doublets were removed according to FSC-A vs FSC-H plot, living CD3+ T cells were selected and in this population those cells expressing CD4 and CD8 were selected. Within CD4+ T cells and CD8+ T cells the expression of CD39, and PD1 was analysed. The expression of these molecules has been investigated also in Treg cells.

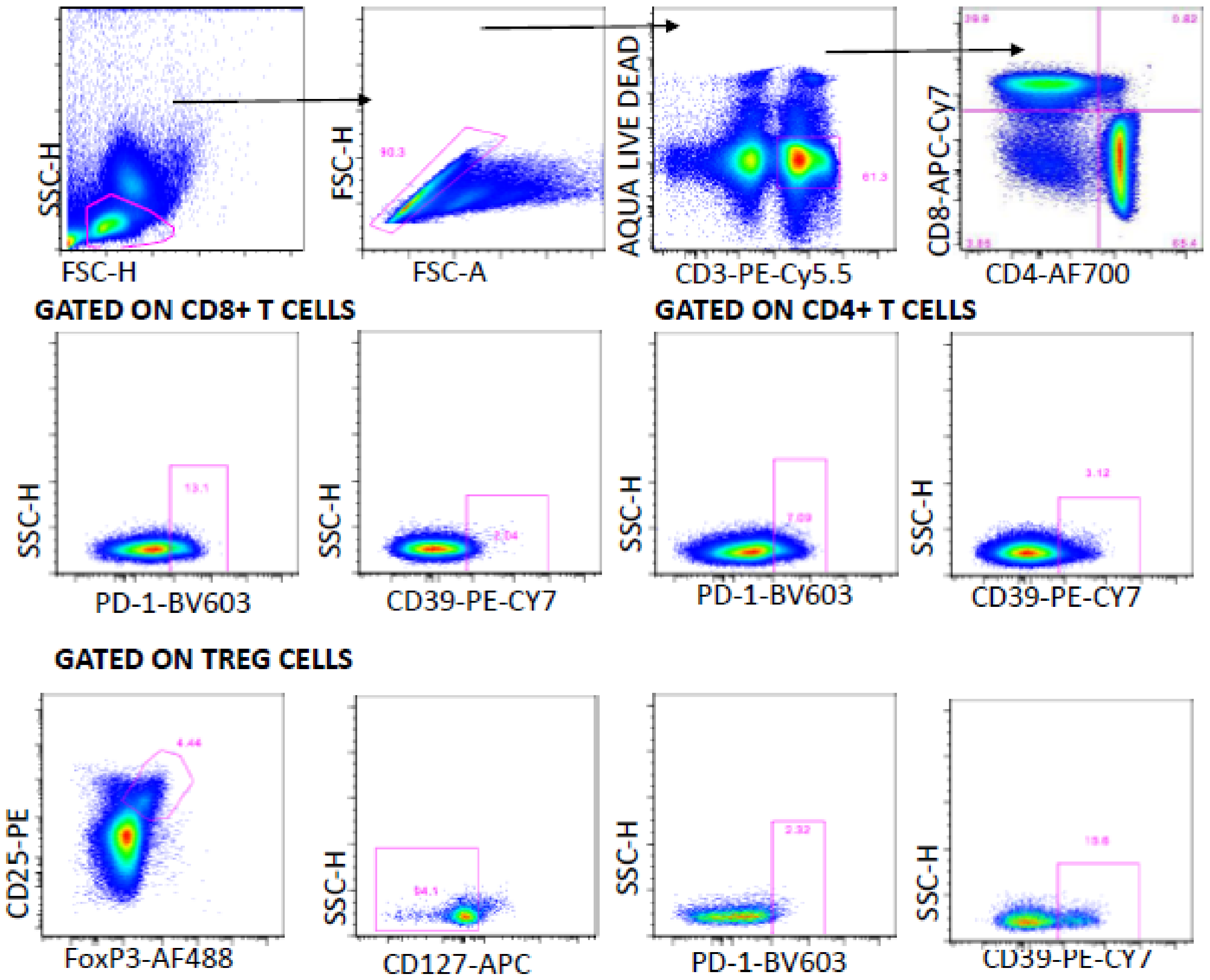

Figure 3.1.2.D. Gating strategy of NK cells. NK cells were identified according to physical parameters, i.e., FSC-H and SSC-H. Then, we excluded cell doublets from the analysis, and we identified live cells. Dump channel containing anti-CD19, -CD4, -CD3, -CD14 was used to get rid of all unwanted cells. In this negative population, cells expressing CD16 and CD56 has been defined as NK. In particular, three populations of NK cells have been identified according to the expression of CD56 (negative, dim and bright). In the main represented population (CD16+, CD56 dim), the expression of CD57, CD62L, HLA-DR, CD158, CD158B have been investigated.

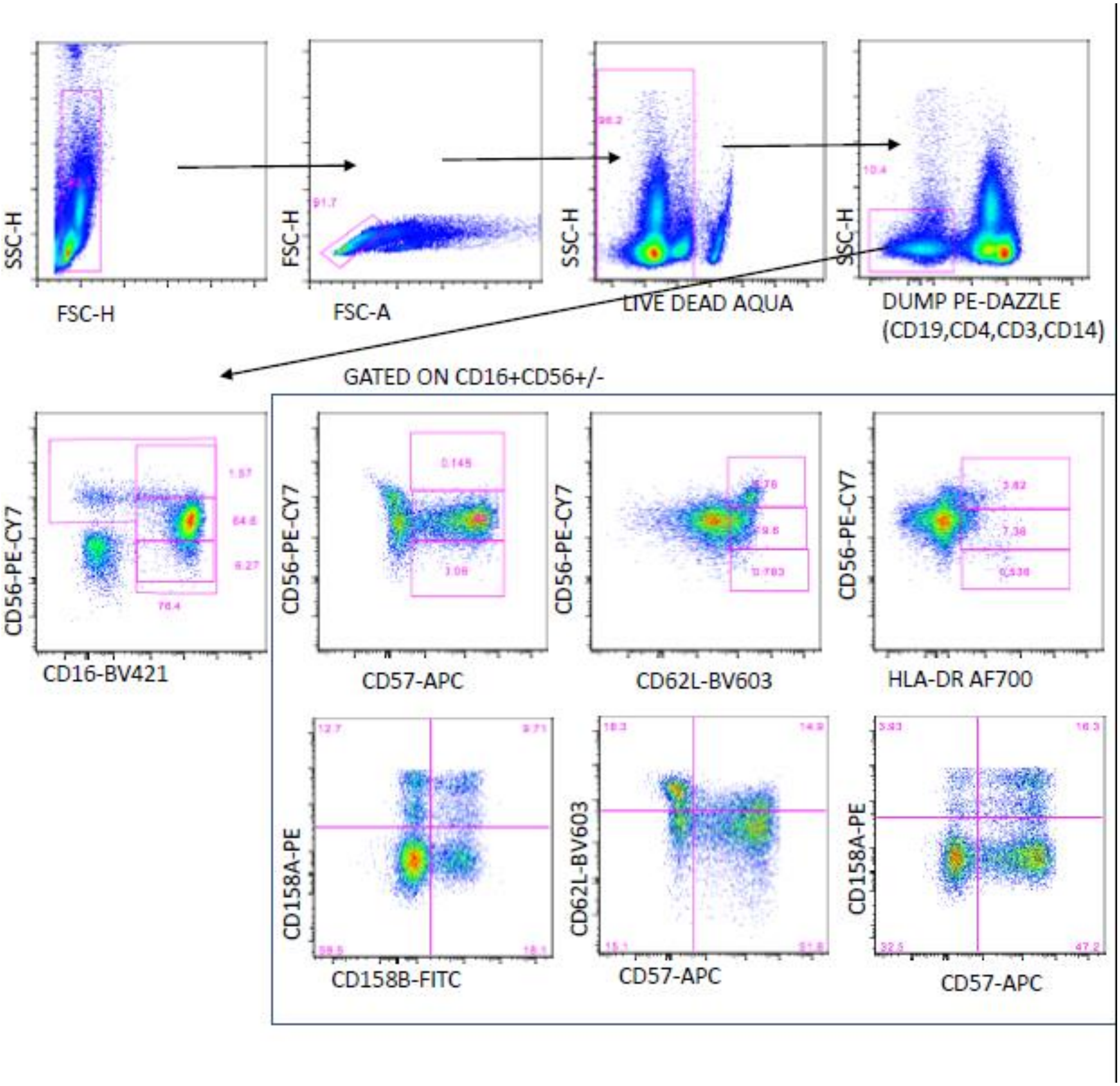

Figure 3.1.2.E. T cell differentiation status and pS6 quantification in CD4+, CD8+ T cells and Treg cells. Lymphocytes have been identified according to physical parameters (FSC-H and SSC-H), doublets were removed according to FSC-A *vs* FSC-H plot, living CD3+ T cells were selected and in this population those cells expressing CD4 and CD8 were selected. Within CD4+ T cells and CD8+ T cells the expression of CD45RA, CCR7 and pS6 was analysed. The expression of these molecules has been investigated also in Treg cells. Naive T cells (N): CD45RA+CCR7+; effector memory T cells (EM): CD45RA-, CCR7; central memory T cells (CM) CD45RA-,CCR7; terminal differentiated effector memory T cells (EMRA): CCR4-,CD45RA+.

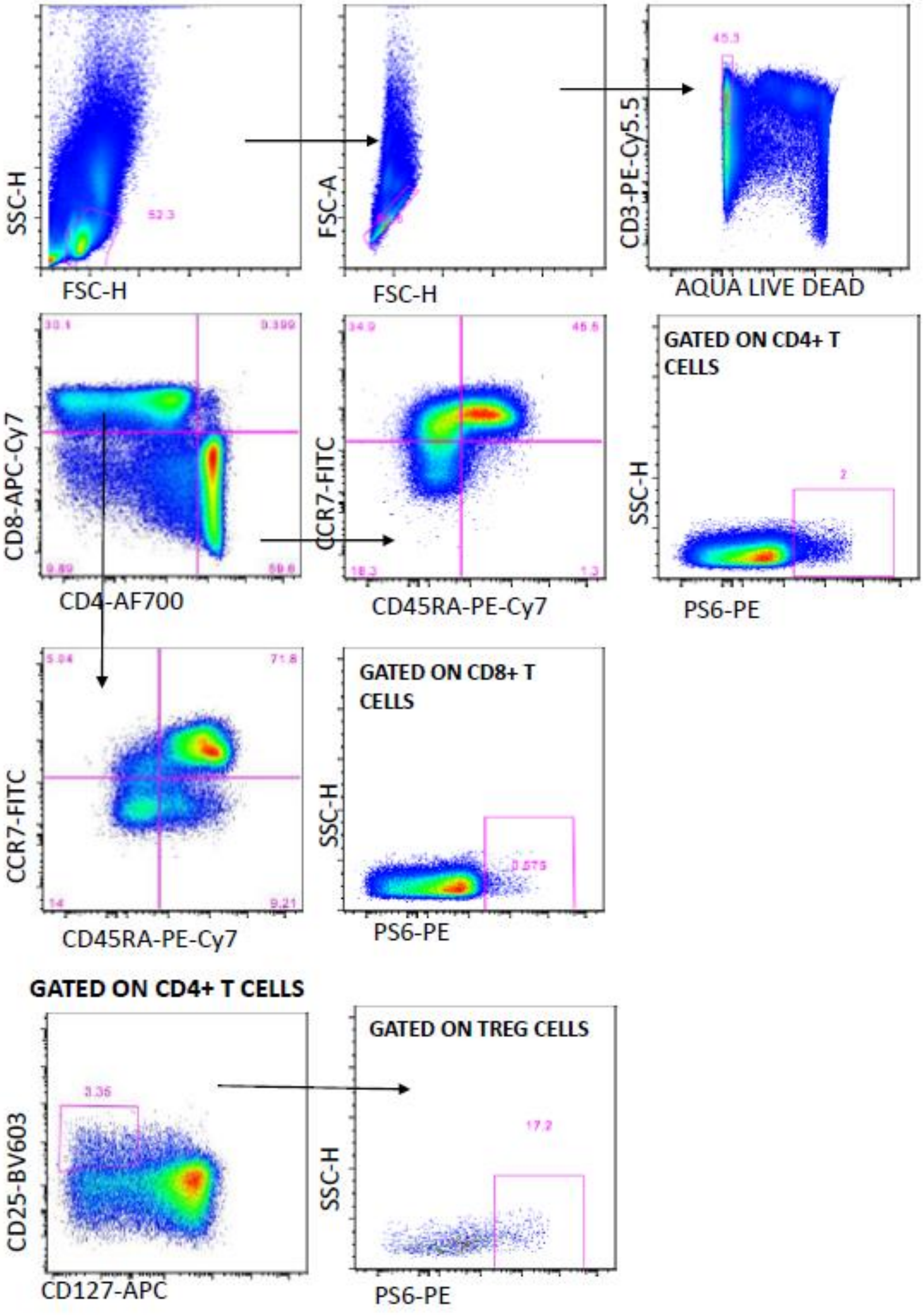

Figure 3.1.2.F. Gating strategy used for monocytes. Monocytes were identified according to physical parameters, i.e., forward scatter-height (FSC-H) and side scatter-height (SSC-H). Then, we excluded cell doublets from the analysis, and we identified alive monocytes that express HLA-DR. Finally, we recognized monocyte subpopulations on the basis of CD14 and CD16 expression: classical (CD14++, CD16-), intermediate (CD14++, CD16+), and non-classical (CD14+, CD16+) monocytes. The median fluorescence intensity (MFI) value for the three membrane receptors CCR2, CCR5, and CXCR4 was evaluated in the different monocyte subsets.

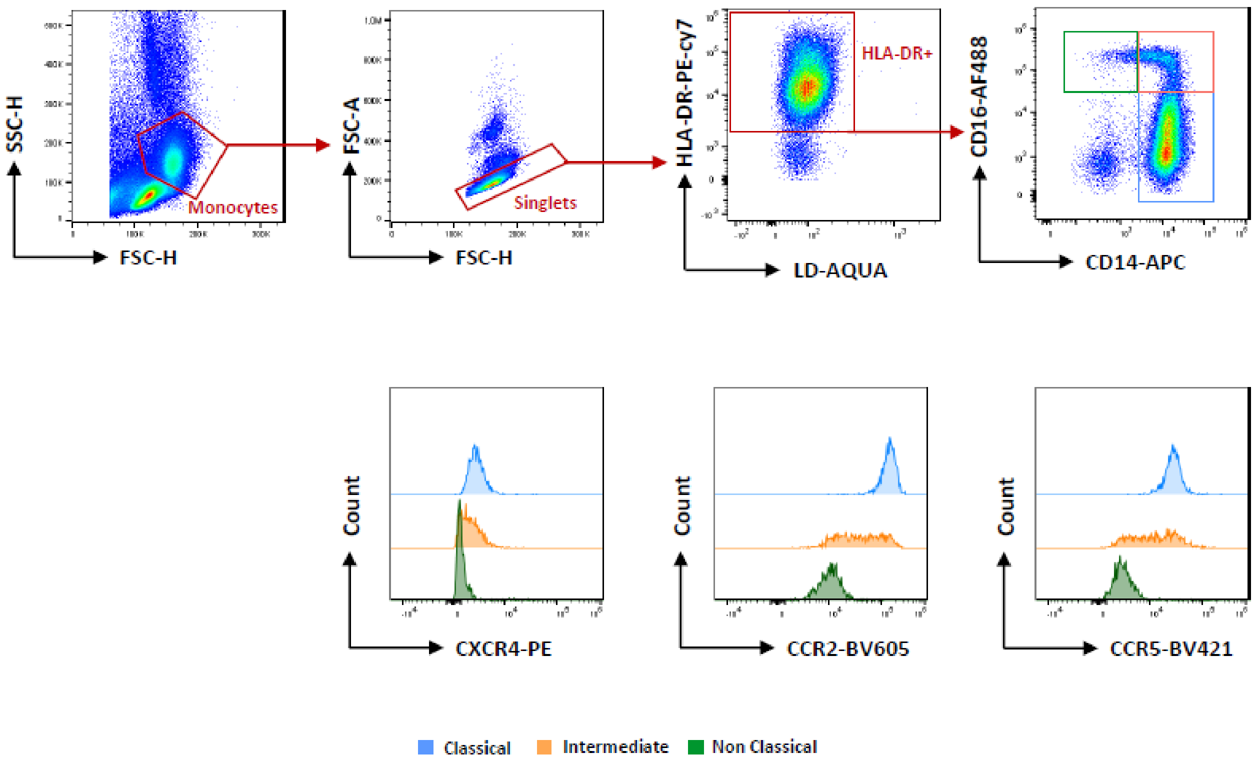

Figure 3.1.2.G. Gating strategy used to identify B cells subpopulations. A first gate was set in IgD and time, then on CD45+ cells, on physical parameters (FSC-H vs FSC-W) to eliminate doublets, then on CD19 and promokine to identify alive B cells. Naïve, exhausted and IgD-IgM expressing B cells were evaluated on B cells gate. Switched memory and unswitched memory were evaluated on IgD- IgM- and IgD+ IgM+ gate, respectively. IgM-only memory B cells were evaluated on IgD- IgM+. Finally, transitional B cells were evaluated among CD38dim, CD38high gate.

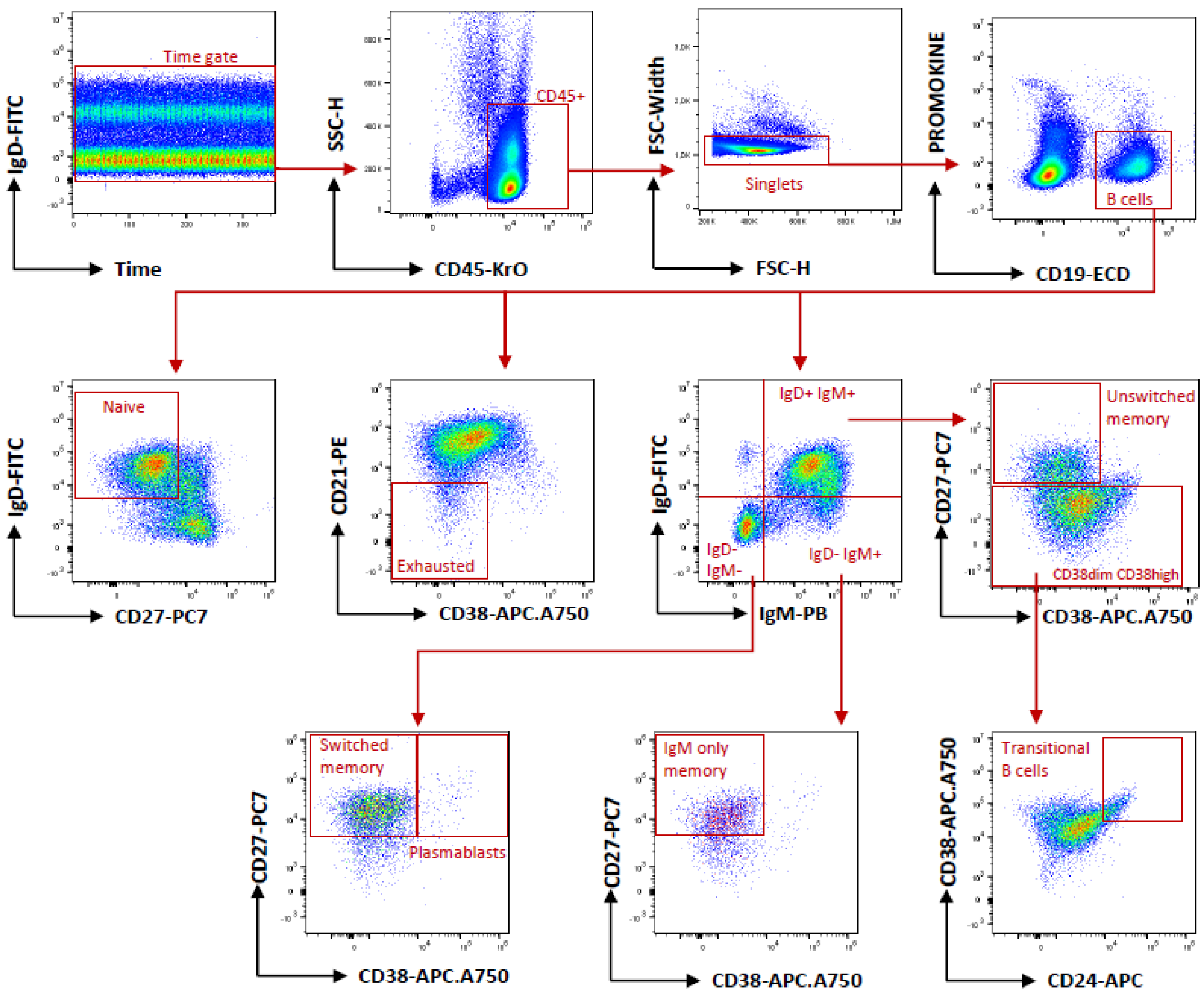

Section 3.1.3 Treg cells function after in vitro stimulation.

Treg functionality was tested through the expression of lymphocyte activation gene-3 (LAG-3), CD49b, IL-10 and the phosphorylation of p38 MAPK. LAG-3 is an inhibitory receptor highly expressed on Treg cells. LAG-3 intrinsically limits Treg cell proliferation and function at inflammatory sites, promotes autoimmunity in a chronic autoimmune-prone environment and may contribute to Treg cell insufficiency in autoimmune disease [42]. CD49b, α2 integrin, defines functionally mature Treg cells. They are short-lived effector Treg cell subset and exhibits a unique tissue distribution, being abundant in peripheral blood. CD49b+ Treg cells, which display superior functionality revealed by in vitro and in vivo assays, appear to develop after multiple rounds of cell division and TCR-dependent activation. They are the apex of the Treg developmental trajectory[43]. IL -10 signaling in regulatory T cells is required for suppression of Th17 cell-mediated inflammation[44]. p38 MAPK is involved in cell cycle control and its activity is a prerequisite for the induction and maintenance of the anergic state in Treg.

Using a subset of patients involved in the study (9 patients receiving 1 mg or 2 mg of Rapamycin, based on frozen PBMC available), Treg functionality have been analyzed after in vitro stimulation with anti-CD3/CD28 (16 hours of in vitro stimulation and 1ug/ml each). All samples were incubated with a protein-transport inhibitor containing brefeldin A (Golgi Plug, Becton Dickinson) Flow cytometry panel has been set up to investigate the expression of LAG-3, IL-10, CD49b and p38 MAPK.

Figure 3.1.3 Gating strategy used to investigate the expression of LAG-3, IL-10, CD49b and p38 MAPK in Treg cells

Treg cells have been identified as CD127-, FoxP3+ cells within alive CD3+, CD4+ T cells. In this population, Treg cells expressing of IL-10, LAG-3, CD49b have been analyzed. Median Fluorescence Intensity (MFI) of p38 MAPK has been quantified.

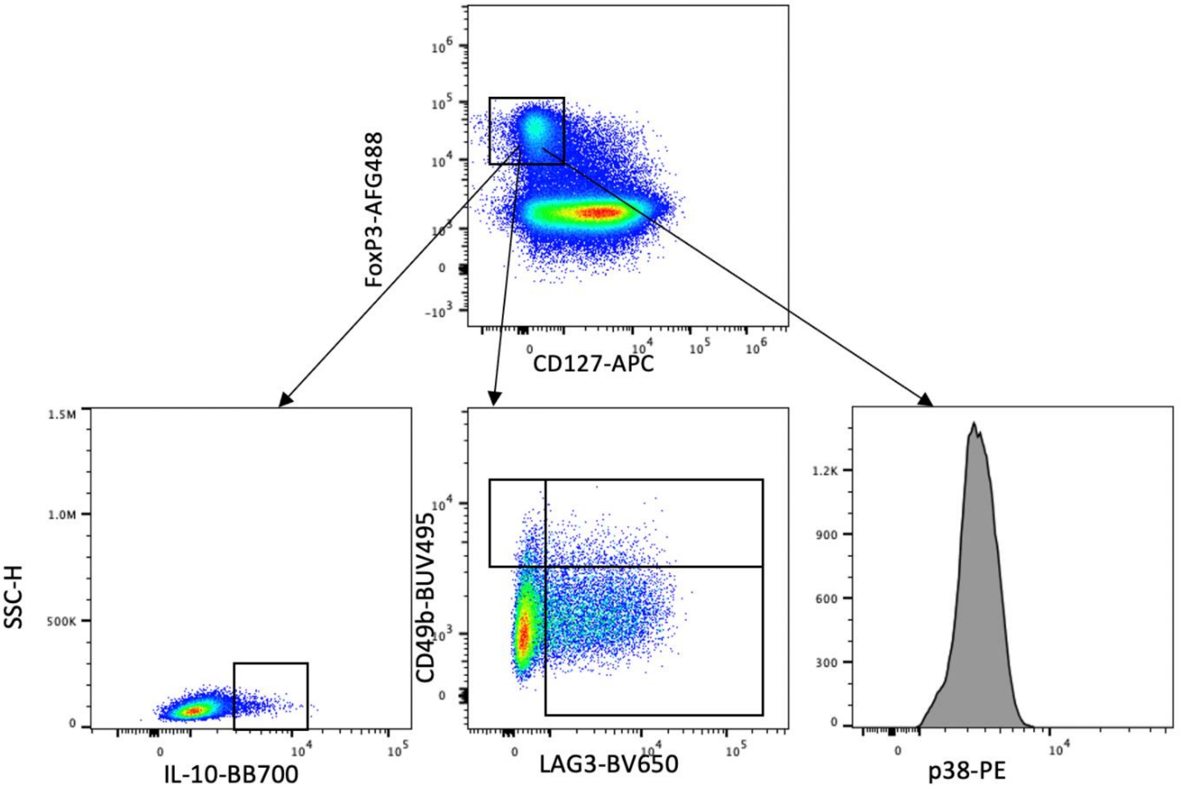

Section 3.1.4 T helper differentiation

Using the frozen PBMC from the same patients analyzed in the previous experiment (section 3.3.3), CD4+ T cells and CD8+ T cells subpopulations have been investigated in terms of T helper differentiation, by analyzing the expression of CXCR3, TBET, CCR4, CCR6 and CD161. In particular, CXCR3+, TBET+ has been defined as Th1, CCR4+ as Th2 and CCR6+,CD161+ as putative mucosal associated invariant T cells (MAIT) or Th17. Moreover, CCR4+,CCR6+ has been defined as Th2/Th17.

Here below a representative example of gating strategy and the percentages of healthy donors (CTR) and ALS patients before (W0) and after 18 weeks of therapy (W18).

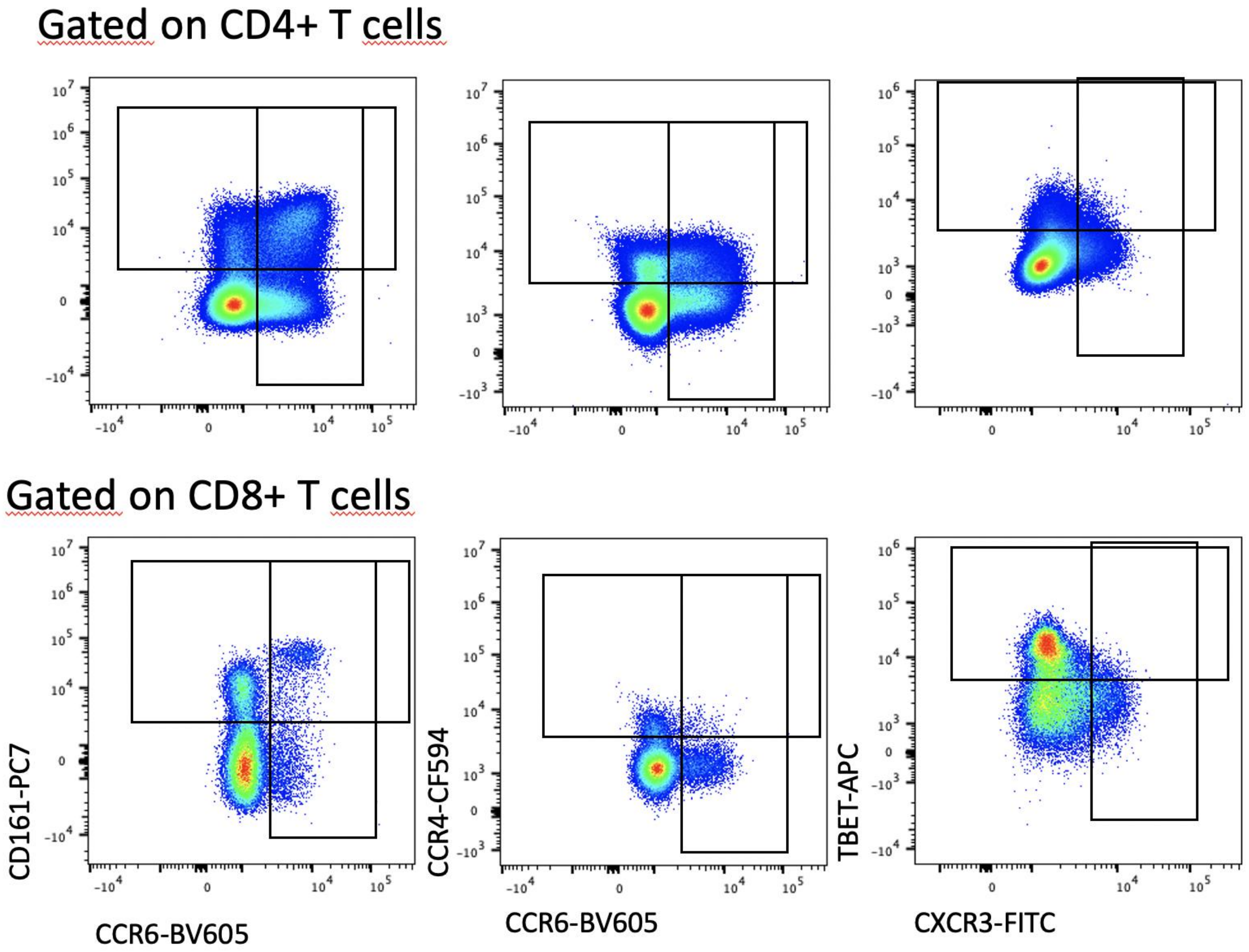

Section 3.1.5 Neurofilament quantification

Cerebrospinal Fluid (CSF) and plasma samples were collected, processed, and stored at -80°C, until quantification for neurofilament heavy and light chain protein (NF). According to manufacturer instructions, CSF and plasma NF levels were determined using an Ella Automated Immunoassay System (Simple Plex Human NF Cartridge, R&D System, Minneapolis, MN). Each sample was measured in triplicate.

Section 3.1.6 Cytokine plasma levels quantification

According to manufacturer instructions, the plasma levels of nine molecules linked to pro and/or anti-inflammatory responses were quantified using a Luminex platform (Human Cytokine Discovery, R&D System, Minneapolis, MN). The following molecules were simultaneously detected: IFN-gamma, IL-1 alpha/IL-1F1, IL-6, IL-10, IL-12 p70, IL-17/IL-17A, IL-18/IL-1F4, TNF-alpha, and TGF-beta.

Section 3.1.7 Monocytes' isolation

CD14+ cells were isolated starting from ten million PBMC, using well-standardized immunomagnetic separation (MACS, Miltenyi, Bergisch Gladbach, Germany). *Ex-vivo* monocytes were immediately stored at -80° until RNA extraction.

Section 3.1.8 RNA extraction and gene expression analyses

Total RNA was extracted from monocytes using the Quick-RNA Miniprep kit (Zymo Research, Irvine, CA) and reverse-transcribed with the iScript cDNA Synthesis Kit (Bio-Rad, Hercules, CA). The quantification of the genes expression was performed using the CFX96 Touch Real-Time PCR Detection System (Bio-Rad, Hercules, CA) and the following pre-validated primer assays (Bio-Rad): *RPS18* (Prime PCR Assay, Bio-Rad; identifier, qHsaCED0037454) as the reference gene, *AIM2* (identifier, qHsaCID0018402), *IL1beta* (identifier, qHsaCID0022272), *IL18* (identifier, qHsaCID0006163), *NAIP* (identifier, qHsaCID0038447), *NLRP3* (identifier, qHsaCID0036694), and *PYCARD* (identifier, qHsaCED0042977). Changes in gene expression were calculated through the Delta-Delta threshold cycle method and referred to patients' gene expression at baseline visit.

Section 3.2. Clinical outcome measures: methods

The Amyotrophic Lateral Sclerosis Functional Rating Scale–Revised (ALSFRS-R) is a clinical scale widely used to measure the functional status of ALS patients. It is made up of 12 questions that cover four main domains representing bulbar, fine motor, gross motor, and respiratory function. The maximum total score is 48 and correspond to complete preservation of the four function. The minimum total score is 0 and represents complete loss of the four functions with patient's dependence on eating, communicating, movements and respiration. The total score is the result of the sum of the scores obtained from each item covered by the 12 questions. Each question can score from 4 (no impairment, normal function) to 0 (no function, complete impairment)[45].

Forced vital capacity (FVC) is a noninvasive test of respiratory function used in clinical practice to assess respiratory function in patients with ALS. FVC was measured in an upright position for at least three trials per assessment and FVC volumes were standardized to the percentage of the predicted normal value on the basis of age, sex, weight and height. The highest score from all attempts was used for analysis.

decline from baseline to weeks 4, 8, 12, 18, 30, 42, and 54, with assessment of 8 muscles for each side of the upper limbs, 7 muscles for each side of the lower limbs, and flexor and extensor muscles of the neck.

The Medical Research Council (MRC) scale for muscular strength is a standard resource in clinical practice that grades muscle power on a scale of 0 to 5 in relation to the maximum expected for that muscle.

For this trial, 16 muscle groups of the neck, upper and lower limbs were tested: neck flexors and neck extensors, left and right deltoid, left and right triceps, left and right biceps, left and right wrist extensor, left and right wrist flexor, left and right fingers extensors, left and right fingers flexors, left and right opponent thumb, left and right iliopsoas, left and right quadriceps, left and right biceps femori, left and right tibialis anterior, left and right suralis triceps, left and right fingers planta flexors, left and right fingers dorsiflexors.

Percentage scores of muscular strength were obtained as the ratio of the observed raw scores divided by the maximum possible score.

Quality of life was measure using the ALSAQ40 questionnaire. ALSAQ40 was scored as indicated by the authors, resulting in five sub-domain scores ranging from 0 to 100: physical mobility; activities of daily living / independence; eating and drinking; communication; emotional functioning. The total ALSAQ40 score was obtained as the unweighted average of the five sub-domain scores.

4. Supplementary Figures

Figure S1. Percentage of Tregs (CD4+, FoxP3+, CD127-) expressing LAG-3 measured in healthy controls and ALS patients before and after experimental treatment.

We assessed percentage of Tregs (CD4+, FoxP3+, CD127-) expressing LAG-3 in 9 patients treated with rapamycin and 4 healthy controls. Demographic features of patients and controls can be found in the following table:

**Group**	**Gender (M/F ratio)**	**Mean Age**
Rapamycin 1mg/m^2/d	0.25	51.80
Rapamycin 2mg	3.00	58.25
CTR	0.50	52.75

Similar percentage of Tregs (CD4+, FoxP3+, CD127-) expressing LAG-3 were measured in healthy controls (4 subjects) and ALS patients (9 patients) before and after experimental treatment.
The same results were obtained as far as IL-10, CD49b and p38 MAPK phosphorylation were considered. These exploratory tests suggest that Tregs from ALS patients were functionally similar to those from age and sex-matched healthy controls (n=4). Moreover, treatment with rapamycin did not induce any changes in Treg functionality. The panel shows the percentages of Tregs expressing LAG-3, CD49b, IL-10 and p38 MAPK within Treg from healthy controls (CTR) and ALS patients before (W0) and after rapamycin treatment (W18). **Data are presented as mean values +/- S.E.M.**

	**CTR *vs* W0 (exact q value)**	**CTR *vs* W18 (exact q value)**	**W0 *vs* W18 (exact q value)**
**LAG-3**	0.76	0.76	0.81
**CD49d**	0.92	0.66	0.66
**IL-10**	0.93	0.93	0.93
**p38 MAPK**	0.85	0.66	0.42

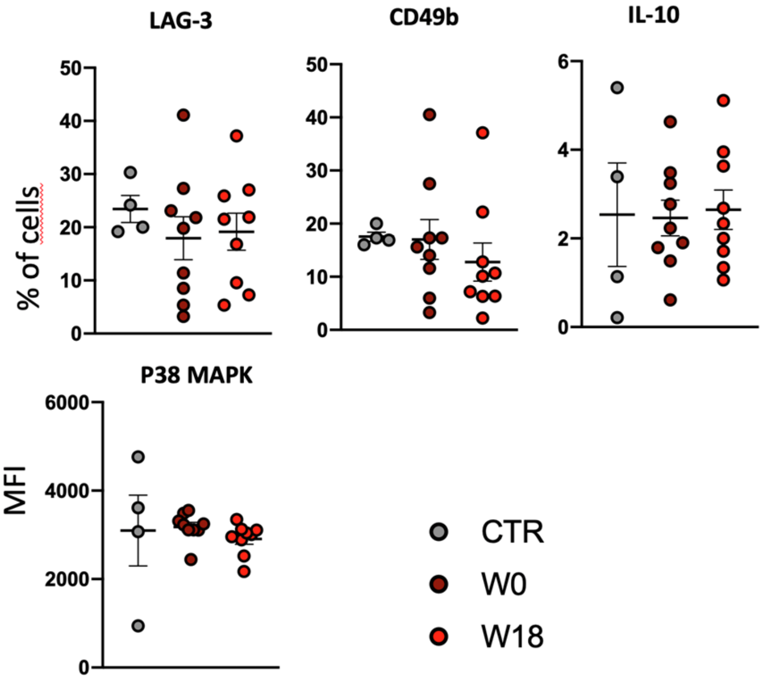

CTR= healthy controls, W0=ALS patients before rapamycin treatment (baseline), W18= ALS patients after rapamycin treatment (treatment end, week 18).
One way Anova for paired samples followed by Original FDR Method Benjamini Hochberg correction
Source data are provided as a Source Data file.

Figure S2: Th1 CD4+ T cells in healthy controls and in ALS patients at baseline and after treatment

Th1 CD4+ T cells were measured in 4 healthy subjects and 9 ALS patients before and after experimental treatment (see table reported for Figure S1).

ALS patients at baseline displayed higher percentages of Th1 CD4+ T cells than healthy controls, but there was no difference in terms of Th1 skewing across groups. This percentage remains similar after 18 weeks of treatment. The same trend has been observed for CD8+ T cells. Data are presented as mean values +/- S.E.M.

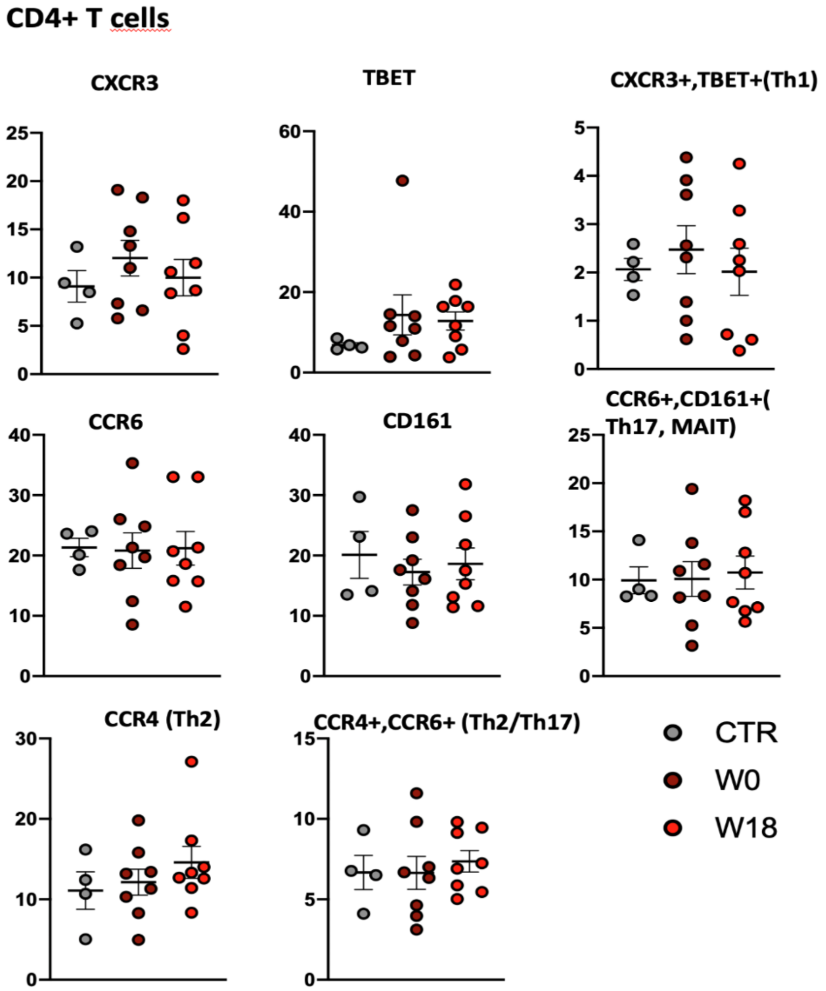

	CTR *vs* W0 (exact q value)	CTR *vs* W18 (exact q value)	W0 *vs* W18 (exact q value)
**CXCR3**	0.63	0.77	0.42
**TBET**	0.50	0.50	0.76
**CXCR3+, TBET+ (Th1)**	0.91	0.95	0.91
**CCR6**	0.97	0.97	0.97
**CD161**	0.73	0.73	0.73
**CCR6+, CD161+ (Th17, MAIT)**	0.95	0.95	0.95
**CCR4**	0.73	0.51	0.51
**CCR4+,CCR6+ (Th2/Th17)**	0.96	0.96	0.96

CTR= healthy controls, W0=ALS patients before rapamycin treatment (baseline), W18= ALS patients after rapamycin treatment (treatment end, week 18). One way Anova for paired samples followed by Original FDR Method Benjamini Hochberg correction was applied. Source data are provided as a Source Data file.

Figure S3. Individual rates of decline in ALSFRS-R total score of patients enrolled in RAP-ALS over the study. Individual rates of decline in ALSFRS-R total score (ITT population) of patients enrolled in RAP-ALS over the study(baseline to week 54) based on treatment arm allocation: placebo (box A), rapamycin 1 mg/m^2/day (box B), rapamycin 2 mg/m^2/day (box C).

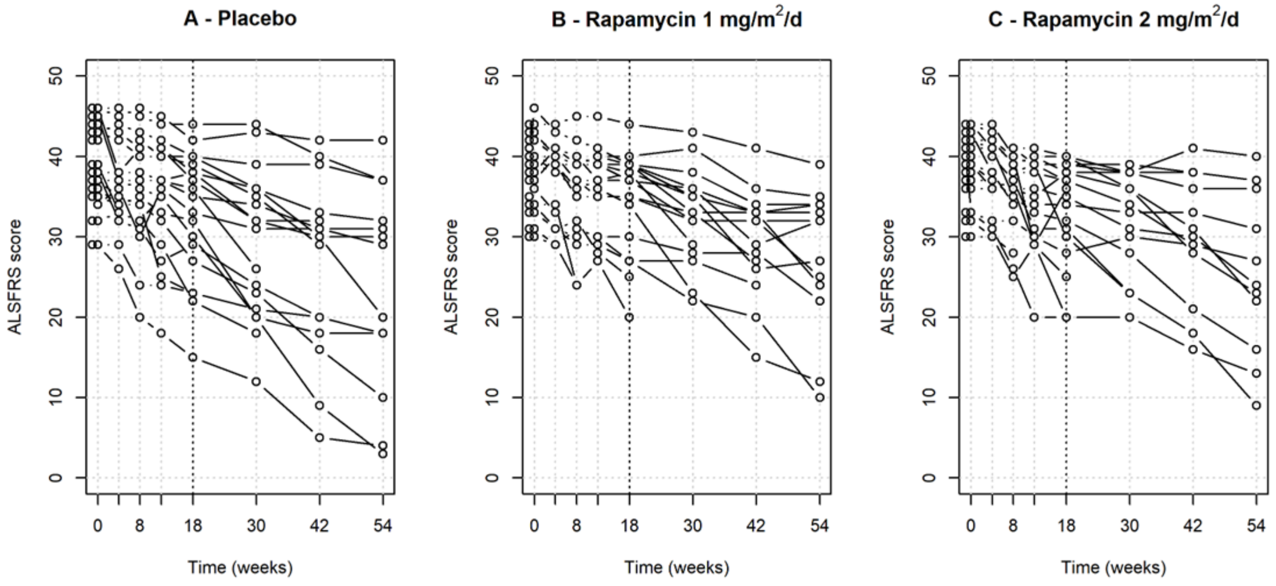

Figure S4. Tracheostomy free survival (post hoc analysis, last observation set on 31st December 2021)
Tracheostomy-free survival of patients enrolled in RAP-ALS based on treatment arm allocation (red = rapamycin 1 mg/m2/d, violet = rapamycin 2 mg/m2/d, blue = placebo). Thick marks represent participants lost to follow-up. The number of participants at risk is displayed in the table. Source data are provided as a Source Data file.

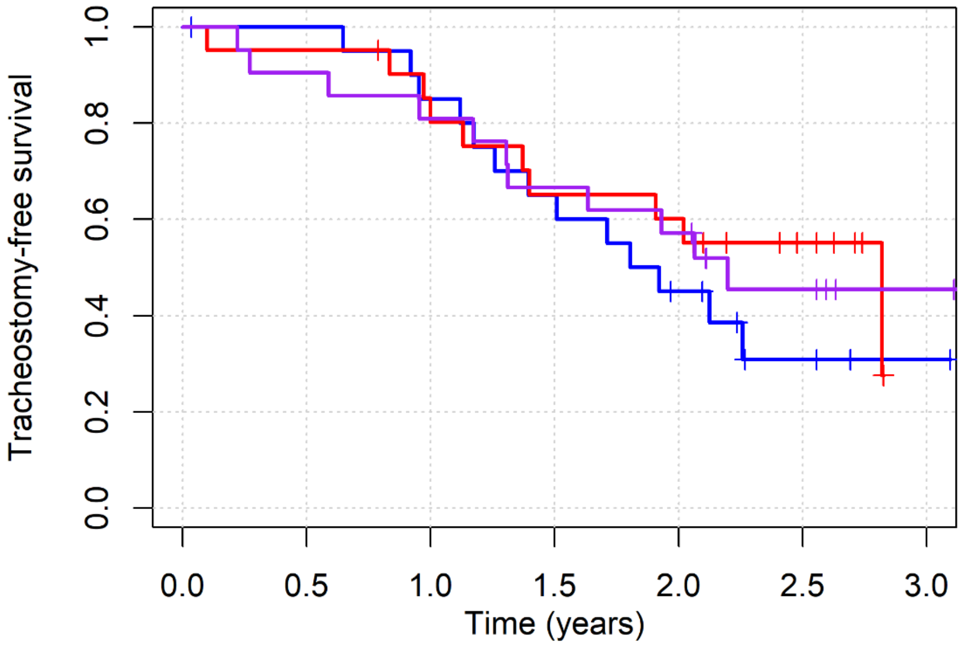

P	21	20	17	13	8	3	1
R1	21	20	16	13	12	6	0
R2	21	19	17	14	12	7	3

Figure S5. Respiratory muscle function as assessed by FVC score decline from baseline to weeks 4, 8, 12, 18, 30, 42, and 54 across treatment arms.
Mean rates of decline in FVC% (ITT population) of patients enrolled in RAP-ALS over the study (baseline to week 54) based on treatment arm allocation (red = rapamycin 1 mg/m2/d, violet = rapamycin 2 mg/m2/d, blue = placebo). Source data are provided as a Source Data file.

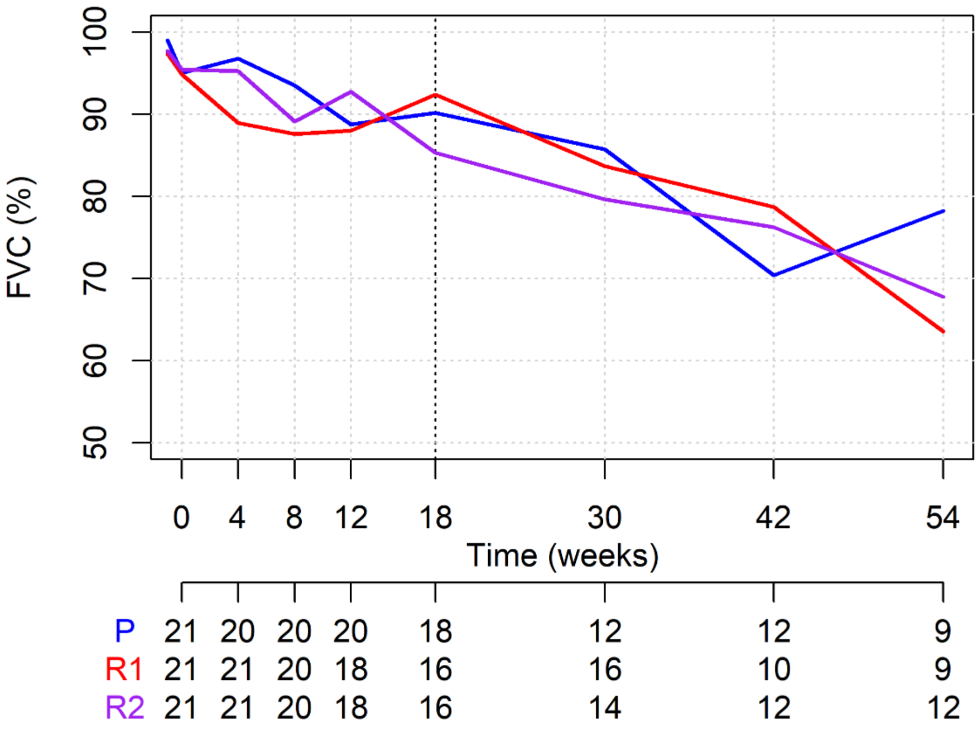

Figure S6: Box plots of mean rapamycin dosages from week 2 (second dosage from baseline) to week 12 (last dosage before treatment end), a-b-c) based on treatment arm allocation (placebo, 1 mg/m2/day, 2 mg/m2/day), d-e-f) based on the dosage assigned during the treatment period just before plasma dosage and clinical evaluations.

Boxes represent upper and lower quartiles and median values, whereas whiskers represent minimum and maximum values.

The number of analysed patients in the placebo arm was 20, whereas in the rapamycin 1 mg/m2/day arm it was 21 up to week 4, 20 from week 6 to 8, and 19 at week 12, and in the rapamycin 2 mg/m2/day arm it was 21 up to week 6, 20 at week 8, and 17 at week 12.

Source data are provided as a Source Data file.

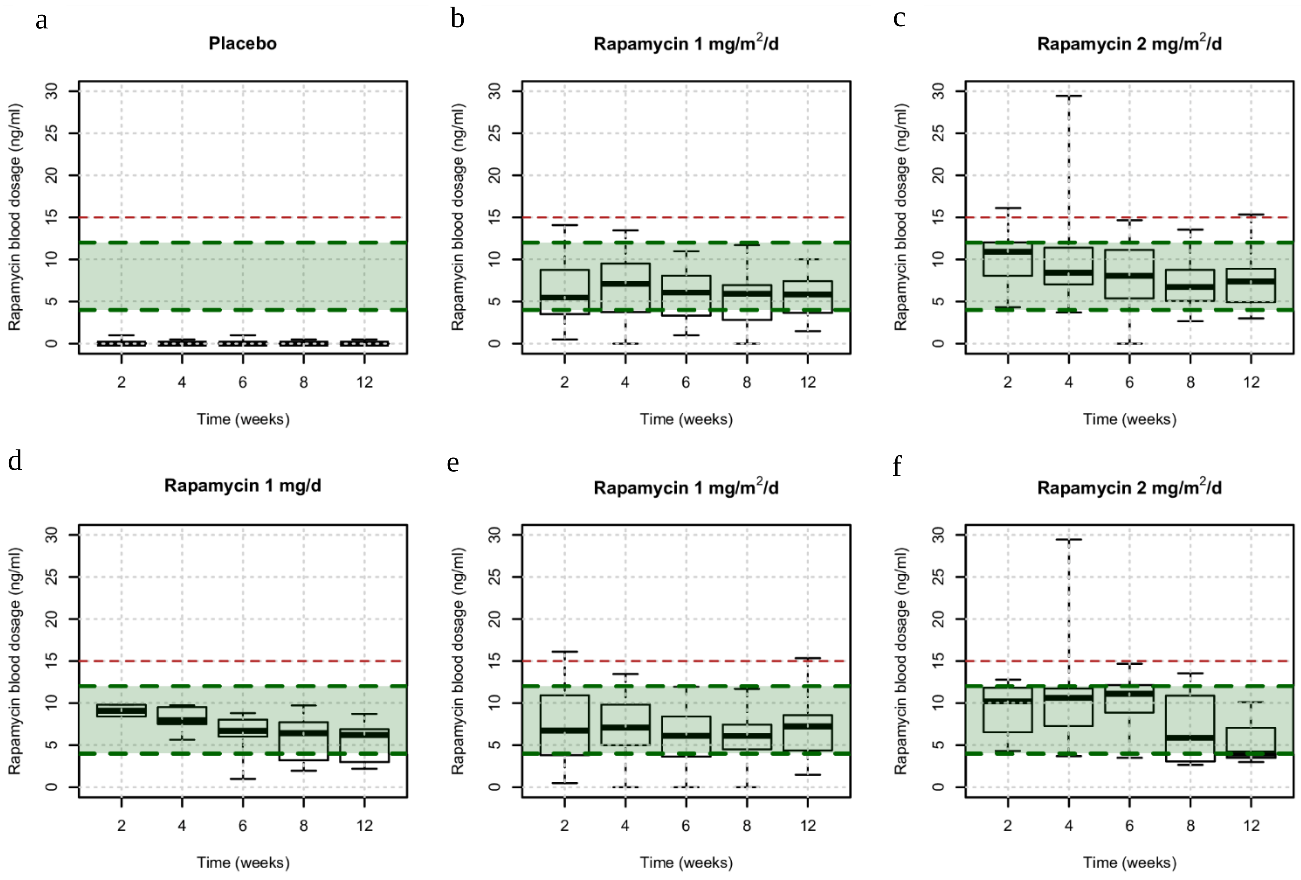

Figure S7. CSF dosage of Sirolimus in 6 CSF samples of patients allocated to the three treatment arms.

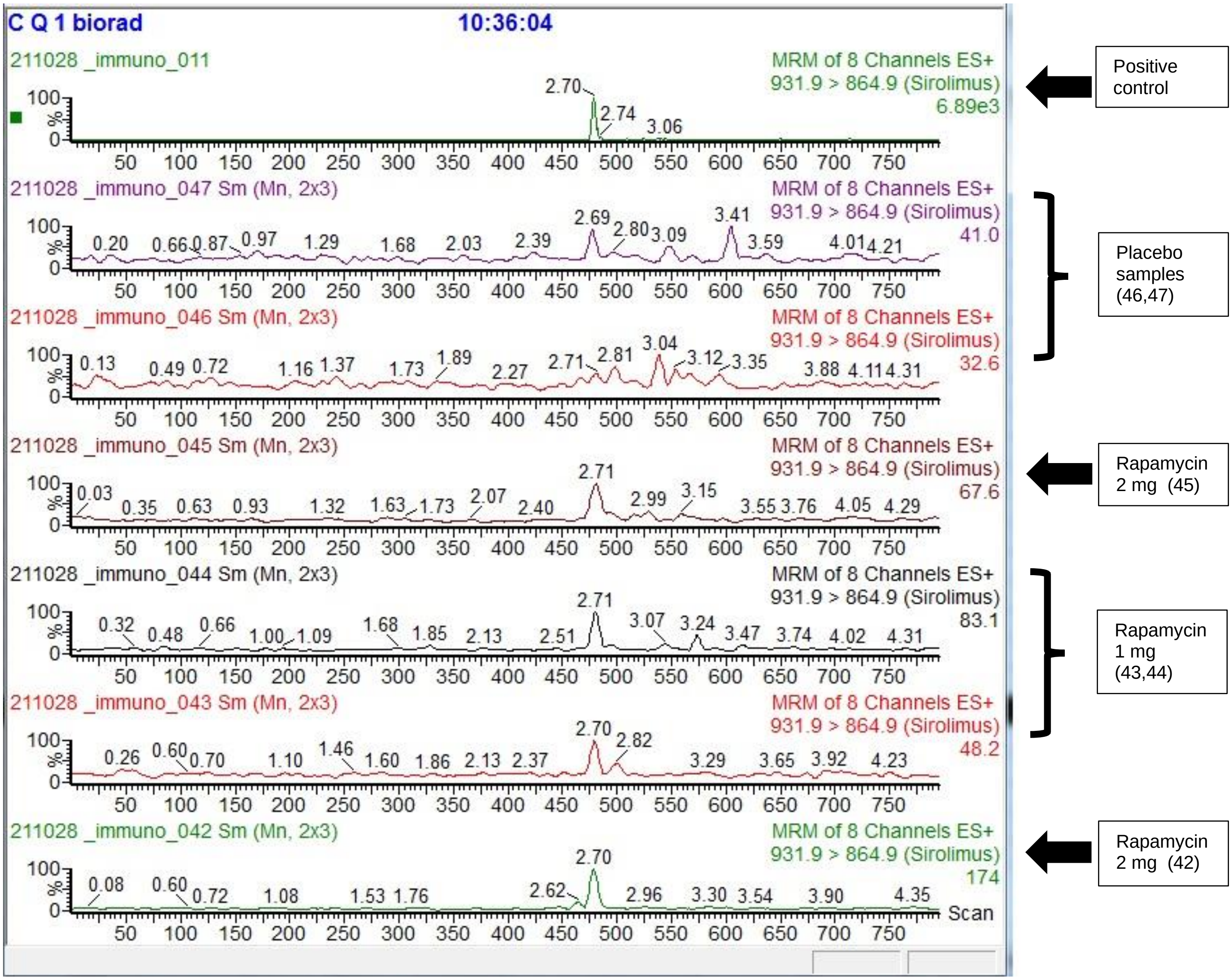

5. Supplementary Tables

Table S1. Biological features of patients enrolled in the three trial arms at baseline

		Placebo (n = 21)		Rapamycin 1 mg/m^2/d (n = 21)		Rapamycin 2 mg/m^2/d (n = 21)	
		mean	SD	mean	SD	mean	SD
CD3+ T CELLS	%	62.52	11.78	60.31	13.57	65.25	14.07
CD4+ T CELLS	%	37.78	9.88	38.75	10.96	41.50	8.05
CD38+,HLA-DR+/CD4+ T CELLS	%	0.71	0.44	0.72	0.50	0.63	0.46
CXCR3+/CD4+ T CELLS	%	23.23	17.88	27.51	13.69	31.29	14.30
CD39+/CD4+ T CELLS	%	3.16	2.35	5.12	2.40	5.85	3.12
PD1+/CD4+ T CELLS	%	5.29	3.13	5.47	2.53	4.49	3.07
TREG/CD4+	%	4.39	2.13	4.17	1.56	5.21	2.06
CD38+,HLA-DR+/ TREG	%	3.75	2.22	3.67	2.67	3.18	1.95
CXCR3+/TREG	%	20.76	14.80	28.81	14.52	32.72	9.99
CD39+/TREG	%	33.84	24.28	44.15	18.77	45.89	20.05
PD1+/TREG	%	1.55	0.80	1.81	1.69	1.05	0.83
CD8+ T CELLS	%	17.87	7.68	18.34	13.24	19.78	6.45
CD38+,HLA-DR+/CD8+ T CELLS	%	0.76	0.59	1.19	1.47	1.19	1.39
CXCR3+/CD8+ T CELLS	%	38.28	32.54	34.93	29.78	59.25	27.36
CD39+/CD8+ T CELLS	%	1.30	1.44	2.08	1.66	2.54	3.73
PD1+/CD8+ T CELLS	%	10.77	6.94	9.78	4.66	8.99	4.90
TREG CD8+	%	0.09	0.09	0.13	0.17	0.09	0.07
TREG CD8+ CD38+ DR+	%	3.17	3.74	2.35	2.89	3.34	3.52
TREG CD8+ CXCR3+	%	17.54	17.24	26.53	22.52	31.85	18.28
TREG CD8+ CD39+	%	42.86	30.31	50.34	28.06	48.28	29.51
TREG CD8+ PD1+	%	0.67	0.66	1.10	1.39	0.79	1.03
B cells/CD45+	%	8.20	4.06	8.05	5.36	9.74	6.17
Naïve B cells/CD45+	%	4.74	2.55	4.45	3.78	6.31	5.65
memory unswitched B cells /CD45+	%	0.63	0.45	0.55	0.62	0.50	0.43
memory switched B cells /CD45+	%	0.90	0.48	1.12	0.79	1.08	0.80
IgM+ B cells /CD45+	%	0.37	0.14	0.54	0.46	0.38	0.18
plasmablasts/CD45+	%	0.09	0.05	0.05	0.03	0.06	0.06
Transitional B cells/CD45+	%	0.17	0.15	0.22	0.24	0.20	0.32
Exhausted B cells/CD45+	%	0.53	0.57	0.33	0.24	0.29	0.26
CD16+,CD56+ NK CELLS	%	12.71	7.67	9.52	3.16	6.86	4.27
CD56++CD62L++/ NK CELLS	%	2.92	1.51	4.00	3.78	5.51	4.92
CD56+-- HLADR+/NK CELLS	%	1.62	1.40	3.08	2.70	2.06	1.89
CD56+- HLADR+/NK CELLS	%	10.54	4.82	15.71	6.51	14.33	11.03
CD57+/NK CELLS	%	44.04	18.49	35.08	11.64	33.86	17.40
CD62L+/NK CELLS	%	8.72	5.08	10.54	8.94	11.32	8.49
HLADR+/NK CELLS	%	2.26	1.11	2.74	2.74	3.06	2.57
CD158A+/NK CELLS	%	15.84	12.76	10.97	5.86	13.45	8.15
CD158A+,CD158B+/NK CELLS	%	12.33	7.47	6.72	2.32	10.35	6.95
CD158B+/NK CELLS	%	20.54	11.57	29.85	13.81	17.42	7.01
Total monocytes	%	17.45	4.74	17.41	5.06	18.24	4.91
Classical monocytes/CD14+	%	84.36	4.77	74.39	17.98	87.44	4.56
Intermediate monocytes/CD14+	%	5.97	4.22	8.78	5.46	4.19	2.40
Not classical monocytes/CD14+	%	4.88	3.36	6.07	3.87	4.02	2.71
PS6/CD8+ T CELLS	%	15.14	9.75	15.85	6.47	5.51	9.53
PS6/TREG	%	28.26	16.75	42.80	11.20	17.42	18.38
PS6/CD4+ T CELLS	%	23.93	14.45	30.35	6.52	13.00	14.03
CM/CD8+ T CELLS	%	8.75	2.80	11.85	10.03	13.90	10.28
NAIVE/CD8+ CELLS	%	22.12	14.75	18.42	10.63	22.59	16.11
EMRA/CD8+ T CELLS	%	29.88	14.23	39.18	23.94	20.99	11.74
EM/CD8+ T CELLS	%	39.10	13.14	30.23	10.31	42.32	16.27
CM/TREG	%	30.74	9.98	37.75	14.06	36.07	12.23
NAIVE/TREG	%	17.58	12.40	9.97	8.04	10.63	18.29
EMRA/TREG	%	2.31	1.97	1.19	1.26	0.70	0.51
EM/TREG	%	49.28	20.87	50.93	17.75	52.44	19.09
CM/CD4+ T CELLS	%	37.90	16.24	35.22	12.83	41.27	9.83

		Placebo (n = 21)		Rapamycin 1 mg/m^2/d (n = 21)		Rapamycin 2 mg/m^2/d (n = 21)	
		mean	SD	mean	SD	mean	SD
NAIVE/CD4+ T CELLS	%	31.30	16.79	42.93	20.15	36.57	13.52
EMRA/CD4+ T CELLS	%	6.48	6.28	7.13	10.88	2.65	2.48
EM/CD4+ T CELLS	%	24.27	14.74	14.69	8.66	19.52	9.67
Inflammasome (RNA) AIM2	AU	7.88	1.10	7.66	1.11	8.21	1.09
IL-1b (mRNA)	AU	4.55	2.89	6.28	1.51	5.41	2.48
IL-18 (mRNA)	AU	7.88	0.97	7.87	0.84	7.67	0.62
Inflammasome (RNA) NAIP	AU	4.67	2.11	5.56	1.52	5.30	1.47
Inflammasome (RNA) NLRP3	AU	5.49	2.00	6.60	1.62	5.89	2.13
Inflammasome (RNA) PYCARD	AU	2.15	0.70	2.07	0.80	2.14	0.52
TGF-B1	pg/ml	40879.70	33694.73	32113.62	22403.71	31447.34	24813.03
IFNy	pg/ml	-	-	727.81	-	-	-
IL-10	pg/ml	-	-	5.44	3.64	-	-
IL-12	pg/ml	125.88	70.51	187.58	194.22	95.49	58.98
IL-17	pg/ml	3.69	0.72	17.14	21.26	5.02	4.05
IL-18	pg/ml	236.94	72.72	289.46	139.71	258.20	92.28
IL-1a	pg/ml	-	-	23.92	-	3.12	-
IL-6	pg/ml	3.87	1.09	4.56	5.45	6.59	11.10
TNFa	pg/ml	3.39	3.40	5.44	5.59	2.72	1.65
pNfH serum	pg/ml	1843.40	1772.31	1830.48	901.40	1291.57	1298.74
pNfH CSF	pg/ml	6730.80	3626.39	6475.48	2601.14	6164.45	3273.17
NfL serum	pg/ml	163.25	126.48	133.05	41.02	108.19	54.25
NfL CSF	pg/ml	12205.20	15475.90	8486.19	6318.99	7573.40	4835.97

AU= Arbitrary Unit (see methods section)

Table S2. Patients exhibiting a positive response (increase in Treg of at least 30%), comparing baseline and treatment end (week 18) between rapamycin and placebo arm. Per Protocol analysis.

	positive response		not positive response		RR	95% CI	p
	n	%	n	%			
Placebo	2	11.8	15	88.2			
Rapamycin 1 mg/m^2/d	4	23.5	13	76.5	2.00	0.42 - 9.50	0.3683
Rapamycin 2 mg/m^2/d	3	20.0	12	80.0	1.70	0.33 - 8.84	0.5220
Rapamycin	7	21.9	25	78.1	1.86	0.43 - 7.98	0.3843

The comparisons were carried out with a chi-square test without any correction. For the comparison of Rapamycin and placebo arms, a P value of 0.05 or less was considered to indicate statistical significance. For the comparisons between Rapamycin 1 mg/m2/d or 2 mg/m2/d arms and the placebo arm, a P value of 0.025 or less was considered to indicate statistical significance to account for multiple arms comparison with the Bonferroni method. All statistical tests were two-tailed. RR = relative risk; CI = confidence interval

Table S3. Changes in Treg cells during and after treatment across treatment arms

Average monthly variations during and after treatment for the placebo group, as well as the comparisons between arms, are shown. Comparisons were performed using segmented repeated measures linear mixed models. Two segments of time were analyzed: during the treatment (after baseline and up to week 18), and after the treatment (after week 18). The dependent variables were the raw measurements of the outcomes, whereas the independent variables were: arm, time (months from baseline) x period (during or after treatment) interaction, and arm x time x period interaction. A random intercept term was also used to account for repeated measurements over the same individual, as well as a random slope term was used to account for individual linear variations over time. Random intercept and random slope terms were kept in the model if they improved the overall goodness-of-fit of the model. For the comparison of Rapamycin and placebo arms, a P value of 0.05 or less was considered to indicate statistical significance. For the comparisons between Rapamycin 1 mg/m2/d or 2 mg/m2/d arms and the placebo arm, a P value of 0.025 or less was considered to indicate statistical significance to account for multiple arms comparison with the Bonferroni method. CIs were calculated based on the exact t distribution using the Satterthwaite's method for degrees of freedom. All statistical tests were two-tailed.

Outcome	Time		Arm	**MD**	**95% CI**		**p**
TREG/CD4+ (1)	During treatment	Monthly variation	Placebo	-0.10	-0.25	0.05	0.1818
	After treatment		Placebo	-0.04	-0.09	0.02	0.2159
	During treatment	Comparison with placebo (monthly variation)	Rapamycin 1 mg	0.17	-0.03	0.38	0.1003
			Rapamycin 2 mg	0.08	-0.13	0.30	0.4503
			Rapamycin	0.13	-0.05	0.31	0.1565
	After treatment		Rapamycin 1 mg	0.04	-0.03	0.12	0.2652
			Rapamycin 2 mg	-0.01	-0.09	0.07	0.7863
			Rapamycin	0.02	-0.05	0.09	0.6098

MD = mean difference; CI = confidence interval.

Table S4. Changes from baseline to week 18 in blood cells population across treatment arms

Time point	Arm	absolute change from baseline			Unadjusted analysis				Adjusted analysis*			
		n	mean	SD	MD	95% CI&		p&	MD	95% CI&		p&
CD3+ T CELLS	Placebo	17	-0.10	4.41	-	-	-	-	-	-	-	-
	Rapamycin 1 mg/m^2/d	18	0.76	9.80	0.86	-4.94	6.66	0.7664	1.15	-5.34	7.63	0.7233
	Rapamycin 2 mg/m^2/d	15	-2.42	10.25	-2.32	-8.39	3.75	0.4461	-2.87	-9.58	3.84	0.3935
	Rapamycin	33	-0.68	9.98	-0.58	-5.71	4.54	0.8194	-0.72	-6.47	5.03	0.8013
CD4+ T CELLS	Placebo	17	0.75	3.54	-	-	-	-	-	-	-	-
	Rapamycin 1 mg/m^2/d	18	1.58	7.68	0.83	-3.36	5.02	0.6926	1.81	-2.80	6.42	0.4324
	Rapamycin 2 mg/m^2/d	15	-3.06	6.43	-3.81	-8.20	0.58	0.0870	-2.69	-7.46	2.08	0.2612
	Rapamycin	33	-0.53	7.41	-1.28	-5.12	2.55	0.5049	-0.28	-4.47	3.90	0.8916
CD38+,HLA-DR+/CD4+ T CELLS	Placebo	12	0.02	0.61	-	-	-	-	-	-	-	-
	Rapamycin 1 mg/m^2/d	10	-0.40	0.61	-0.43	-0.88	0.03	0.0681	-0.55	-1.07	-0.04	0.0358
	Rapamycin 2 mg/m^2/d	10	-0.02	0.23	-0.05	-0.51	0.41	0.8336	-0.10	-0.66	0.46	0.7136
	Rapamycin	20	-0.21	0.49	-0.24	-0.64	0.16	0.2381	-0.36	-0.84	0.11	0.1261
CXCR3+/CD4+ T CELLS	Placebo	12	-0.19	6.96	-	-	-	-	-	-	-	-
	Rapamycin 1 mg/m^2/d	9	0.78	10.91	0.96	-7.74	9.67	0.8219	-1.64	-11.14	7.86	0.7242
	Rapamycin 2 mg/m^2/d	9	4.77	11.26	4.96	-3.75	13.66	0.2529	-2.53	-13.51	8.45	0.6376
	Rapamycin	18	2.77	10.95	2.96	-4.36	10.28	0.4141	-1.98	-10.32	6.37	0.6296
CD39+/CD4+ T CELLS	Placebo	13	0.78	3.41	-	-	-	-	-	-	-	-
	Rapamycin 1 mg/m^2/d	10	1.19	6.04	0.41	-3.11	3.93	0.8146	1.91	-1.98	5.79	0.3232
	Rapamycin 2 mg/m^2/d	13	0.78	2.74	-0.01	-3.29	3.28	0.9960	0.90	-2.99	4.78	0.6401
	Rapamycin	23	0.96	4.37	0.17	-2.69	3.03	0.9028	1.40	-1.90	4.71	0.3926
PD1+/CD4+ T CELLS	Placebo	13	-0.09	2.74	-	-	-	-	-	-	-	-
	Rapamycin 1 mg/m^2/d	10	0.63	5.25	0.71	-2.62	4.05	0.6667	2.42	-1.29	6.13	0.1926
	Rapamycin 2 mg/m^2/d	13	1.48	3.68	1.57	-1.54	4.68	0.3117	3.07	-0.65	6.78	0.1019
	Rapamycin	23	1.11	4.34	1.20	-1.52	3.91	0.3770	2.74	-0.41	5.89	0.0854
TREG/CD4+	Placebo	17	-0.46	1.58	-	-	-	-	-	-	-	-
	Rapamycin 1 mg/m^2/d	18	0.41	1.56	0.87	-0.20	1.93	0.1088	0.53	-0.56	1.62	0.3327
	Rapamycin 2 mg/m^2/d	15	-0.05	1.55	0.41	-0.71	1.52	0.4655	0.09	-1.04	1.22	0.8757
	Rapamycin	33	0.20	1.55	0.66	-0.28	1.59	0.1645	0.32	-0.63	1.28	0.4985
CD38+,HLA-DR+/ TREG	Placebo	12	0.27	2.70	-	-	-	-	-	-	-	-
	Rapamycin 1 mg/m^2/d	10	-1.18	2.64	-1.45	-3.56	0.66	0.1692	-1.31	-3.58	0.97	0.2482
	Rapamycin 2 mg/m^2/d	10	-0.07	1.68	-0.35	-2.46	1.76	0.7374	-0.27	-2.75	2.21	0.8216
	Rapamycin	20	-0.63	2.22	-0.90	-2.70	0.90	0.3139	-0.87	-2.89	1.14	0.3811
CXCR3+/TREG	Placebo	12	-1.37	8.39	-	-	-	-	-	-	-	-
	Rapamycin 1 mg/m^2/d	10	-5.16	14.83	-3.79	-13.88	6.31	0.4489	-5.85	-16.25	4.56	0.2581
	Rapamycin 2 mg/m^2/d	10	-4.92	11.05	-3.54	-13.64	6.55	0.4787	-12.31	-23.65	-0.96	0.0347
	Rapamycin	20	-5.04	12.73	-3.67	-12.12	4.79	0.3828	-8.55	-17.89	0.78	0.0709
CD39+/TREG	Placebo	13	7.28	21.75	-	-	-	-	-	-	-	-
	Rapamycin 1 mg/m^2/d	9	-1.73	5.54	-9.02	-21.80	3.77	0.1605	-12.20	-28.04	3.63	0.1255
	Rapamycin 2 mg/m^2/d	13	7.38	8.07	0.10	-11.47	11.67	0.9861	-6.27	-20.68	8.14	0.3802
	Rapamycin	22	3.65	8.37	-3.63	-14.10	6.84	0.4857	-8.66	-21.68	4.37	0.1845
PD1+/TREG	Placebo	13	-0.15	0.87	-	-	-	-	-	-	-	-
	Rapamycin 1 mg/m^2/d	9	-0.80	1.84	-0.65	-1.66	0.37	0.2028	-0.54	-1.85	0.77	0.4065
	Rapamycin 2 mg/m^2/d	13	0.46	0.70	0.62	-0.30	1.53	0.1791	0.69	-0.50	1.89	0.2433

Time point	Arm	absolute change from baseline			Unadjusted analysis				Adjusted analysis*			
		n	mean	SD	MD	95% CI[&]		p[&]	MD	95% CI[&]		p[&]
	Rapamycin	22	-0.05	1.40	0.10	-0.78	0.98	0.8177	0.20	-0.95	1.34	0.7253
CD8+ T CELLS	Placebo	13	-0.31	3.70	-	-	-	-	-	-	-	-
	Rapamycin 1 mg/m²/d	9	0.31	4.79	0.63	-3.15	4.41	0.7367	0.62	-4.21	5.45	0.7925
	Rapamycin 2 mg/m²/d	10	0.07	4.47	0.38	-3.29	4.05	0.8330	-0.34	-4.83	4.14	0.8761
	Rapamycin	19	0.18	4.49	0.50	-2.58	3.58	0.7437	0.07	-3.88	4.02	0.9715
CD38+,HLA-DR+/CD8+ T CELLS	Placebo	12	-0.06	0.51	-	-	-	-	-	-	-	-
	Rapamycin 1 mg/m²/d	9	-0.98	1.46	-0.92	-1.75	-0.08	0.0321	-0.93	-2.02	0.17	0.0935
	Rapamycin 2 mg/m²/d	8	-0.51	0.55	-0.44	-1.31	0.42	0.2997	-0.13	-1.22	0.97	0.8152
	Rapamycin	17	-0.76	1.12	-0.70	-1.41	0.02	0.0556	-0.53	-1.49	0.44	0.2692
CXCR3+/CD8+ T CELLS	Placebo	12	-2.61	23.77	-	-	-	-	-	-	-	-
	Rapamycin 1 mg/m²/d	9	4.00	15.03	6.62	-12.29	25.53	0.4784	7.71	-17.01	32.42	0.5246
	Rapamycin 2 mg/m²/d	8	-0.03	21.71	2.58	-17.00	22.16	0.7886	2.90	-21.86	27.65	0.8105
	Rapamycin	17	2.10	17.98	4.72	-11.17	20.61	0.5474	5.31	-15.48	26.09	0.6024
CD39+/CD8+ T CELLS	Placebo	13	0.31	0.96	-	-	-	-	-	-	-	-
	Rapamycin 1 mg/m²/d	9	0.00	1.07	-0.31	-1.08	0.47	0.4250	-0.40	-1.35	0.56	0.4005
	Rapamycin 2 mg/m²/d	10	-0.15	0.46	-0.46	-1.21	0.29	0.2223	-0.64	-1.53	0.25	0.1501
	Rapamycin	19	-0.08	0.79	-0.39	-1.02	0.25	0.2221	-0.54	-1.32	0.25	0.1709
PD1+/CD8+ T CELLS	Placebo	13	0.32	3.19	-	-	-	-	-	-	-	-
	Rapamycin 1 mg/m²/d	9	-1.17	5.04	-1.48	-5.91	2.94	0.4980	-1.40	-7.29	4.49	0.6289
	Rapamycin 2 mg/m²/d	10	3.45	6.63	3.13	-1.16	7.42	0.1461	3.00	-2.47	8.47	0.2699
	Rapamycin	19	1.26	6.24	0.95	-2.90	4.79	0.6193	1.12	-3.91	6.15	0.6506
TREG CD8+	Placebo	13	0.03	0.13	-	-	-	-	-	-	-	-
	Rapamycin 1 mg/m²/d	9	-0.04	0.19	-0.07	-0.19	0.04	0.2097	-0.13	-0.27	0.02	0.0834
	Rapamycin 2 mg/m²/d	10	0.00	0.04	-0.04	-0.15	0.08	0.5351	-0.05	-0.18	0.09	0.4886
	Rapamycin	19	-0.02	0.13	-0.05	-0.15	0.04	0.2677	-0.08	-0.20	0.04	0.1842
TREG CD8+ CD38+ DR+	Placebo	12	1.08	4.70	-	-	-	-	-	-	-	-
	Rapamycin 1 mg/m²/d	9	-1.57	3.77	-2.65	-6.99	1.69	0.2216	-3.18	-8.54	2.19	0.2328
	Rapamycin 2 mg/m²/d	9	1.15	5.75	0.07	-4.27	4.41	0.9738	0.80	-4.42	6.01	0.7550
	Rapamycin	18	-0.21	4.92	-1.29	-4.98	2.40	0.4805	-1.08	-5.82	3.67	0.6437
TREG CD8+ CXCR3+	Placebo	12	-1.38	11.92	-	-	-	-	-	-	-	-
	Rapamycin 1 mg/m²/d	9	-4.00	16.70	-2.62	-16.06	10.81	0.6916	-1.20	-16.49	14.09	0.8727
	Rapamycin 2 mg/m²/d	9	6.71	16.42	8.08	-5.35	21.51	0.2276	13.31	-1.56	28.19	0.0769
	Rapamycin	18	1.35	16.98	2.73	-8.87	14.33	0.6337	6.48	-7.46	20.41	0.3472
TREG CD8+ CD39+	Placebo	13	1.29	26.68	-	-	-	-	-	-	-	-
	Rapamycin 1 mg/m²/d	9	-2.65	10.15	-3.94	-21.28	13.40	0.6459	-3.76	-26.80	19.28	0.7395
	Rapamycin 2 mg/m²/d	10	-1.18	13.81	-2.46	-19.28	14.36	0.7667	-5.31	-26.71	16.09	0.6138
	Rapamycin	19	-1.87	11.91	-3.16	-17.30	10.98	0.6513	-4.65	-23.41	14.12	0.6149
TREG CD8+ PD1+	Placebo	13	0.44	1.60	-	-	-	-	-	-	-	-
	Rapamycin 1 mg/m²/d	9	-0.30	1.12	-0.74	-1.98	0.50	0.2329	-0.85	-2.24	0.54	0.2175
	Rapamycin 2 mg/m²/d	10	0.18	1.35	-0.26	-1.46	0.95	0.6663	0.19	-1.09	1.48	0.7580
	Rapamycin	19	-0.05	1.23	-0.49	-1.51	0.54	0.3396	-0.25	-1.44	0.93	0.6663
B cells/CD45+	Placebo	7	-0.75	3.13	-	-	-	-	-	-	-	-
	Rapamycin 1 mg/m²/d	5	1.82	4.44	2.57	-1.74	6.87	0.2274	4.23	-0.94	9.40	0.1016
	Rapamycin 2 mg/m²/d	10	0.53	3.27	1.27	-2.35	4.90	0.4712	3.98	-1.13	9.09	0.1179
	Rapamycin	15	0.96	3.60	1.70	-1.60	5.01	0.2954	4.10	-0.29	8.50	0.0654
Naïve B cells/CD45+	Placebo	7	-0.42	2.14	-	-	-	-	-	-	-	-
	Rapamycin 1 mg/m²/d	5	1.08	2.59	1.50	-1.46	4.46	0.3029	2.58	-0.91	6.07	0.1356

Time point	Arm	absolute change from baseline			Unadjusted analysis				Adjusted analysis*			
		n	mean	SD	MD	95% CI&		p&	MD	95% CI&		p&
	Rapamycin 2 mg/m^2/d	10	0.69	2.51	1.11	-1.38	3.61	0.3612	3.14	-0.30	6.59	0.0709
	Rapamycin	15	0.82	2.45	1.24	-1.01	3.49	0.2638	2.87	-0.11	5.85	0.0578
memory unswitched B cells /CD45+	Placebo	7	-0.31	0.57	-	-	-	-	-	-	-	-
	Rapamycin 1 mg/m^2/d	5	0.17	0.37	0.47	-0.02	0.97	0.0579	0.65	0.08	1.21	0.0277
	Rapamycin 2 mg/m^2/d	10	-0.02	0.25	0.29	-0.13	0.70	0.1610	0.35	-0.21	0.91	0.2032
	Rapamycin	15	0.04	0.30	0.35	-0.03	0.73	0.0691	0.49	-0.01	1.00	0.0543
memory switched B cells /CD45+	Placebo	7	-0.25	0.42	-	-	-	-	-	-	-	-
	Rapamycin 1 mg/m^2/d	5	0.58	0.44	0.82	0.35	1.29	0.0016	0.98	0.42	1.55	0.0022
	Rapamycin 2 mg/m^2/d	10	0.02	0.32	0.26	-0.13	0.66	0.1779	0.34	-0.22	0.90	0.2159
	Rapamycin	15	0.20	0.45	0.45	0.03	0.87	0.0359	0.65	0.07	1.24	0.0307
IgM+ B cells /CD45+	Placebo	7	-0.05	0.05	-	-	-	-	-	-	-	-
	Rapamycin 1 mg/m^2/d	5	-0.06	0.29	-0.01	-0.22	0.20	0.9229	0.05	-0.21	0.31	0.6901
	Rapamycin 2 mg/m^2/d	10	-0.02	0.15	0.03	-0.15	0.21	0.7136	0.05	-0.21	0.30	0.7050
	Rapamycin	15	-0.03	0.20	0.02	-0.14	0.18	0.8202	0.05	-0.17	0.27	0.6511
plasmablasts/CD45+	Placebo	7	-0.01	0.06	-	-	-	-	-	-	-	-
	Rapamycin 1 mg/m^2/d	5	-0.02	0.02	-0.01	-0.07	0.04	0.5435	-0.01	-0.06	0.05	0.8368
	Rapamycin 2 mg/m^2/d	10	-0.02	0.03	-0.01	-0.06	0.03	0.4832	0.01	-0.05	0.06	0.8346
	Rapamycin	15	-0.02	0.02	-0.01	-0.05	0.02	0.4347	0.00	-0.05	0.05	0.9944
Transitional B cells/CD45+	Placebo	7	0.04	0.09	-	-	-	-	-	-	-	-
	Rapamycin 1 mg/m^2/d	5	-0.12	0.27	-0.16	-0.35	0.03	0.0998	-0.15	-0.39	0.09	0.1937
	Rapamycin 2 mg/m^2/d	10	-0.09	0.12	-0.13	-0.29	0.03	0.1160	-0.13	-0.37	0.11	0.2542
	Rapamycin	15	-0.10	0.18	-0.14	-0.29	0.01	0.0637	-0.14	-0.35	0.06	0.1590
Exhausted B cells/CD45+	Placebo	7	-0.16	0.43	-	-	-	-	-	-	-	-
	Rapamycin 1 mg/m^2/d	5	-0.07	0.23	0.09	-0.28	0.47	0.6062	0.28	-0.15	0.72	0.1831
	Rapamycin 2 mg/m^2/d	10	-0.09	0.23	0.07	-0.24	0.39	0.6381	0.30	-0.13	0.73	0.1565
	Rapamycin	15	-0.08	0.22	0.08	-0.21	0.37	0.5677	0.29	-0.08	0.66	0.1122
CD16+,CD56+ NK CELLS	Placebo	4	-1.97	3.63	-	-	-	-	-	-	-	-
	Rapamycin 1 mg/m^2/d	3	-1.59	2.44	0.38	-6.33	7.10	0.9018	9.47	-6.37	25.31	0.1939
	Rapamycin 2 mg/m^2/d	6	1.69	4.57	3.67	-2.01	9.34	0.1805	10.66	-3.19	24.51	0.1087
	Rapamycin	9	0.60	4.15	2.57	-2.74	7.88	0.3092	10.64	-1.89	23.18	0.0847
CD56++CD62L++/ NK CELLS	Placebo	4	-1.03	1.67	-	-	-	-	-	-	-	-
	Rapamycin 1 mg/m^2/d	3	-1.69	5.29	-0.66	-7.05	5.74	0.8235	-10.84	-24.54	2.86	0.1011
	Rapamycin 2 mg/m^2/d	6	1.02	3.92	2.06	-3.35	7.46	0.4165	-5.63	-17.61	6.35	0.2941
	Rapamycin	9	0.12	4.30	1.15	-3.83	6.13	0.6208	-5.70	-19.24	7.84	0.3525
CD56+-- HLADR+/NK CELLS	Placebo	4	-0.24	0.21	-	-	-	-	-	-	-	-
	Rapamycin 1 mg/m^2/d	3	-0.09	1.08	0.15	-3.13	3.43	0.9227	0.43	-8.58	9.45	0.9105
	Rapamycin 2 mg/m^2/d	6	0.44	2.63	0.67	-2.10	3.45	0.5995	0.26	-7.62	8.15	0.9373
	Rapamycin	9	0.26	2.17	0.50	-1.95	2.95	0.6627	0.27	-6.79	7.32	0.9313
CD56+- HLADR+/NK CELLS	Placebo	4	-0.98	3.12	-	-	-	-	-	-	-	-
	Rapamycin 1 mg/m^2/d	3	-8.56	11.88	-7.58	-26.92	11.77	0.4033	-11.56	-68.97	45.85	0.6397
	Rapamycin 2 mg/m^2/d	6	2.78	14.01	3.76	-12.59	20.11	0.6191	2.32	-47.88	52.52	0.9137
	Rapamycin	9	-1.00	13.79	-0.02	-15.71	15.68	0.9982	2.13	-47.91	52.16	0.9228
CD57+/NK CELLS	Placebo	4	-1.38	5.92	-	-	-	-	-	-	-	-
	Rapamycin 1 mg/m^2/d	3	-8.29	5.54	-6.92	-30.26	16.42	0.5238	3.37	-62.42	69.15	0.9045
	Rapamycin 2 mg/m^2/d	6	5.39	18.52	6.76	-12.96	26.49	0.4626	11.65	-45.88	69.18	0.6380

Time point	Arm	absolute change from baseline			Unadjusted analysis				Adjusted analysis*			
		n	mean	SD	MD	95% CI[&]		p[&]	MD	95% CI[&]		p[&]
	Rapamycin	9	0.83	16.39	2.20	-16.74	21.14	0.8028	11.53	-41.59	64.65	0.6236
CD62L+/NK CELLS	Placebo	4	-0.87	3.32	-	-	-	-	-	-	-	-
	Rapamycin 1 mg/m^2/d	3	-7.13	13.49	-6.26	-19.70	7.18	0.3237	-20.51	-51.65	10.64	0.1583
	Rapamycin 2 mg/m^2/d	6	6.07	6.73	6.94	-4.42	18.29	0.2034	-2.94	-30.18	24.29	0.8004
	Rapamycin	9	1.67	10.83	2.54	-9.89	14.97	0.6619	-3.19	-40.23	33.85	0.8445
HLADR+/NK CELLS	Placebo	4	-0.71	0.93	-	-	-	-	-	-	-	-
	Rapamycin 1 mg/m^2/d	3	-1.08	2.93	-0.37	-3.10	2.36	0.7680	-4.68	-9.84	0.48	0.0681
	Rapamycin 2 mg/m^2/d	6	-0.01	1.09	0.70	-1.61	3.01	0.5134	-2.20	-6.70	2.31	0.2784
	Rapamycin	9	-0.36	1.78	0.34	-1.77	2.46	0.7267	-2.23	-7.87	3.41	0.3810
CD158A+/NK CELLS	Placebo	4	0.52	0.76	-	-	-	-	-	-	-	-
	Rapamycin 1 mg/m^2/d	3	-0.22	0.36	-0.74	-3.15	1.67	0.5096	3.04	-2.11	8.19	0.1990
	Rapamycin 2 mg/m^2/d	6	0.01	1.90	-0.51	-2.55	1.53	0.5913	2.70	-1.81	7.20	0.1932
	Rapamycin	9	-0.07	1.52	-0.59	-2.38	1.21	0.4874	2.70	-1.36	6.77	0.1601
CD158A+,CD158B+/NK CELLS	Placebo	4	-0.09	2.35	-	-	-	-	-	-	-	-
	Rapamycin 1 mg/m^2/d	3	1.17	1.20	1.26	-1.79	4.31	0.3797	1.92	-5.04	8.87	0.5259
	Rapamycin 2 mg/m^2/d	6	1.06	1.60	1.15	-1.43	3.73	0.3453	1.06	-5.02	7.15	0.6844
	Rapamycin	9	1.10	1.40	1.19	-1.08	3.45	0.2738	1.07	-4.54	6.68	0.6646
CD158B+/NK CELLS	Placebo	4	1.85	1.79	-	-	-	-	-	-	-	-
	Rapamycin 1 mg/m^2/d	3	1.87	2.80	0.02	-8.68	8.72	0.9957	1.93	-20.79	24.65	0.8424
	Rapamycin 2 mg/m^2/d	6	4.34	6.87	2.49	-4.86	9.84	0.4678	2.41	-17.46	22.28	0.7763
	Rapamycin	9	3.51	5.74	1.67	-4.93	8.26	0.5890	2.41	-15.39	20.20	0.7585
Total monocytes	Placebo	7	1.51	7.15	-	-	-	-	-	-	-	-
	Rapamycin 1 mg/m^2/d	5	-3.16	5.64	-4.66	-11.41	2.08	0.1621	-4.88	-13.49	3.73	0.2406
	Rapamycin 2 mg/m^2/d	7	0.61	2.53	-0.89	-7.05	5.27	0.7629	-4.62	-12.14	2.90	0.2058
	Rapamycin	12	-0.96	4.34	-2.46	-7.98	3.05	0.3594	-4.71	-11.49	2.07	0.1575
Classical monocytes/CD14+	Placebo	7	-9.36	16.54	-	-	-	-	-	-	-	-
	Rapamycin 1 mg/m^2/d	5	8.40	9.62	17.76	3.32	32.20	0.0191	11.34	0.13	22.56	0.0478
	Rapamycin 2 mg/m^2/d	7	-3.79	5.06	5.57	-7.61	18.75	0.3835	-5.23	-15.02	4.57	0.2676
	Rapamycin	12	1.29	9.33	10.65	-1.76	23.05	0.0878	0.33	-12.76	13.42	0.9577
Intermediate monocytes/CD14+	Placebo	7	8.20	15.61	-	-	-	-	-	-	-	-
	Rapamycin 1 mg/m^2/d	5	-5.28	5.81	-13.48	-26.08	-0.88	0.0376	-5.47	-15.97	5.02	0.2781
	Rapamycin 2 mg/m^2/d	7	0.92	2.89	-7.27	-18.78	4.23	0.1987	1.14	-8.02	10.31	0.7902
	Rapamycin	12	-1.66	5.20	-9.86	-20.07	0.35	0.0575	-1.07	-10.19	8.04	0.8032
Not classical monocytes/CD14+	Placebo	7	1.12	3.21	-	-	-	-	-	-	-	-
	Rapamycin 1 mg/m^2/d	5	-1.68	4.91	-2.80	-7.02	1.41	0.1772	-4.82	-10.44	0.80	0.0864
	Rapamycin 2 mg/m^2/d	7	2.85	2.08	1.73	-2.11	5.58	0.3533	1.66	-3.25	6.57	0.4768
	Rapamycin	12	0.96	4.07	-0.16	-3.96	3.65	0.9316	-0.51	-6.33	5.30	0.8514
CM/CD8+ T CELLS	Placebo	4	1.65	4.85	-	-	-	-	-	-	-	-
	Rapamycin 1 mg/m^2/d	3	1.83	2.15	0.18	-17.90	18.26	0.9828	-5.05	-40.43	30.33	0.7288
	Rapamycin 2 mg/m^2/d	5	5.42	15.05	3.77	-12.11	19.66	0.6039	-1.10	-35.32	33.13	0.9375
	Rapamycin	8	4.08	11.58	2.43	-11.29	16.14	0.7018	-2.66	-32.39	27.07	0.8340
NAIVE/CD8+ CELLS	Placebo	4	2.42	6.20	-	-	-	-	-	-	-	-
	Rapamycin 1 mg/m^2/d	3	-0.33	3.50	-2.75	-18.54	13.04	0.7027	-10.65	-38.67	17.37	0.3735
	Rapamycin 2 mg/m^2/d	5	8.67	12.37	6.25	-7.62	20.12	0.3348	0.35	-26.76	27.46	0.9748
	Rapamycin	8	5.29	10.61	2.87	-10.10	15.85	0.6322	-4.00	-33.58	25.58	0.7519

Time point	Arm	absolute change from baseline			Unadjusted analysis				Adjusted analysis*			
		n	mean	SD	MD	95% CI[&]		p[&]	MD	95% CI[&]		p[&]
EMRA/CD8+ T CELLS	Placebo	4	2.03	11.22	-	-	-	-	-	-	-	-
	Rapamycin 1 mg/m^2/d	3	-7.57	8.55	-9.59	-27.62	8.44	0.2594	-16.29	-56.56	23.99	0.3462
	Rapamycin 2 mg/m^2/d	5	-2.73	10.68	-4.76	-20.59	11.07	0.5137	-12.30	-51.26	26.66	0.4540
	Rapamycin	8	-4.55	9.61	-6.57	-20.38	7.23	0.3138	-13.88	-47.54	19.79	0.3521
EM/CD8+ T CELLS	Placebo	4	-6.13	12.19	-	-	-	-	-	-	-	-
	Rapamycin 1 mg/m^2/d	3	6.13	9.92	12.26	-17.81	42.33	0.3805	31.39	-40.21	103.00	0.3109
	Rapamycin 2 mg/m^2/d	5	-9.78	22.82	-3.66	-30.07	22.75	0.7612	14.04	-55.22	83.31	0.6245
	Rapamycin	8	-3.81	19.84	2.31	-22.10	26.72	0.8372	20.91	-44.70	86.51	0.4651
CM/TREG	Placebo	4	1.78	9.96	-	-	-	-	-	-	-	-
	Rapamycin 1 mg/m^2/d	3	-8.23	12.55	-10.01	-39.38	19.36	0.4605	-22.32	-99.16	54.52	0.4889
	Rapamycin 2 mg/m^2/d	5	8.08	22.29	6.31	-19.49	32.10	0.5938	-3.65	-77.99	70.68	0.9044
	Rapamycin	8	1.96	20.01	0.19	-23.83	24.21	0.9865	-11.04	-81.48	59.40	0.7146
NAIVE/TREG	Placebo	4	6.37	11.89	-	-	-	-	-	-	-	-
	Rapamycin 1 mg/m^2/d	3	-1.59	8.21	-7.96	-30.35	14.42	0.4417	-16.28	-55.51	22.94	0.3347
	Rapamycin 2 mg/m^2/d	5	6.04	15.43	-0.33	-19.99	19.33	0.9702	-9.97	-47.91	27.97	0.5293
	Rapamycin	8	3.18	13.07	-3.20	-20.56	14.17	0.6905	-12.47	-46.32	21.38	0.4022
EMRA/TREG	Placebo	4	0.32	2.24	-	-	-	-	-	-	-	-
	Rapamycin 1 mg/m^2/d	3	-0.37	1.76	-0.70	-3.72	2.32	0.6127	-0.32	-5.74	5.10	0.8855
	Rapamycin 2 mg/m^2/d	5	0.63	1.24	0.30	-2.35	2.95	0.8028	0.21	-5.03	5.45	0.9216
	Rapamycin	8	0.25	1.43	-0.07	-2.41	2.26	0.9452	0.00	-4.53	4.53	0.9995
EM/TREG	Placebo	4	-8.50	4.90	-	-	-	-	-	-	-	-
	Rapamycin 1 mg/m^2/d	3	10.47	16.85	18.97	-24.78	62.72	0.3524	39.51	-69.03	148.04	0.3924
	Rapamycin 2 mg/m^2/d	5	-14.44	35.81	-5.94	-44.37	32.48	0.7344	13.98	-91.02	118.98	0.7461
	Rapamycin	8	-5.10	31.31	3.40	-32.53	39.33	0.8374	24.08	-74.79	122.95	0.5730
CM/CD4+ T CELLS	Placebo	4	0.60	5.83	-	-	-	-	-	-	-	-
	Rapamycin 1 mg/m^2/d	3	2.90	3.85	2.30	-8.78	13.38	0.6500	3.88	-19.19	26.95	0.6836
	Rapamycin 2 mg/m^2/d	5	6.48	7.72	5.88	-3.86	15.62	0.2050	7.54	-14.78	29.86	0.4250
	Rapamycin	8	5.14	6.46	4.54	-4.03	13.11	0.2654	6.09	-13.79	25.98	0.4819
NAIVE/CD4+ T CELLS	Placebo	4	3.43	3.95	-	-	-	-	-	-	-	-
	Rapamycin 1 mg/m^2/d	3	-6.37	10.43	-9.79	-20.44	0.86	0.0673	-22.96	-45.39	-0.53	0.0465
	Rapamycin 2 mg/m^2/d	5	-0.10	4.40	-3.53	-12.88	5.83	0.4161	-16.08	-37.78	5.62	0.1152
	Rapamycin	8	-2.45	7.26	-5.88	-14.67	2.92	0.1675	-18.80	-40.56	2.96	0.0789
EMRA/CD4+ T CELLS	Placebo	4	-2.43	5.73	-	-	-	-	-	-	-	-
	Rapamycin 1 mg/m^2/d	3	0.34	0.60	2.77	-3.29	8.84	0.3284	0.69	-10.92	12.29	0.8852
	Rapamycin 2 mg/m^2/d	5	-1.56	1.71	0.87	-4.46	6.20	0.7196	-1.90	-13.12	9.33	0.6821
	Rapamycin	8	-0.85	1.66	1.58	-3.10	6.27	0.4681	-0.88	-11.34	9.59	0.8446

Time point	Arm	absolute change from baseline			Unadjusted analysis				Adjusted analysis*			
		n	mean	SD	MD	95% CI[&]		p[&]	MD	95% CI[&]		p[&]
EM/CD4+ T CELLS	Placebo	4	-1.55	5.21	-	-	-	-	-	-	-	-
	Rapamycin 1 mg/m^2/d	3	3.13	7.07	4.68	-10.28	19.65	0.4970	17.94	-16.59	52.46	0.2394
	Rapamycin 2 mg/m^2/d	5	-4.51	11.11	-2.96	-16.11	10.19	0.6228	10.17	-23.23	43.57	0.4693
	Rapamycin	8	-1.64	10.03	-0.09	-12.18	12.00	0.9866	13.24	-17.95	44.43	0.3389

Mean absolute changes from baseline to week 18 are showed for each treatment group and comparison were performed using linear regression models that include indicator variables for treatment arms as the independent variables. For the comparison of Rapamycin and placebo arms, a P value of 0.05 or less was considered to indicate statistical significance. For the comparisons between Rapamycin 1 mg/m2/d or 2 mg/m2/d arms and the placebo arm, a P value of 0.025 or less was considered to indicate statistical significance to account for multiple arms comparison with the Bonferroni method. CIs were calculated based on the exact t distribution. All statistical tests were two-tailed.
The number of outcomes that were tested here was 55, and no correction was applied for multiple outcomes.
MD = mean difference; CI = confidence interval.

*Adjusted analyses for sex, ALSFRS-R slope at baseline, disease duration from onset to baseline and edaravone treatment.

Table S5. Changes from baseline to week 18 in inflammasome and cytokines across treatment arms

Outcome	Arm	Absolute change from baseline*			Unadjusted analysis				Adjusted analysis§			
		n	mean	SD	MD	95% CI		p	MD	95% CI		p
**Inflammasome (RNA) AIM2**	Placebo	7	0.77	2.36	-	-	-	-	-	-	-	-
	Rapamycin 1 mg/m²/d	8	0.63	2.87	-0.14	-2.33	2.06	0.8989	1.39	-0.39	3.17	0.1184
	Rapamycin 2 mg/m²/d	10	-0.41	0.24	-1.17	-3.26	0.92	0.2568	0.81	-1.01	2.62	0.3640
	Rapamycin	18	0.05	1.93	-0.71	-2.60	1.18	0.4436	1.12	-0.50	2.74	0.1658
**IL-1b (mRNA)**	Placebo	7	-0.33	0.42	-	-	-	-	-	-	-	-
	Rapamycin 1 mg/m²/d	8	1.50	3.88	1.83	-1.04	4.70	0.2015	3.08	0.23	5.93	0.0355
	Rapamycin 2 mg/m²/d	12	0.93	2.47	1.26	-1.38	3.90	0.3349	0.82	-2.05	3.70	0.5565
	Rapamycin	20	1.15	3.03	1.49	-0.91	3.88	0.2127	1.98	-0.80	4.76	0.1536
**IL-18 (mRNA)**	Placebo	7	0.34	0.81	-	-	-	-	-	-	-	-
	Rapamycin 1 mg/m²/d	8	-0.11	0.46	-0.45	-1.01	0.10	0.1015	-0.48	-1.17	0.21	0.1593
	Rapamycin 2 mg/m²/d	12	-0.26	0.29	-0.60	-1.10	-0.09	0.0228	-0.69	-1.38	0.01	0.0522
	Rapamycin	20	-0.20	0.36	-0.54	-1.00	-0.08	0.0234	-0.58	-1.20	0.04	0.0648
**Inflammasome (RNA) NAIP**	Placebo	7	-0.22	0.93	-	-	-	-	-	-	-	-
	Rapamycin 1 mg/m²/d	8	-0.20	0.49	0.02	-0.87	0.91	0.9642	0.19	-0.86	1.25	0.7061
	Rapamycin 2 mg/m²/d	12	-0.24	0.94	-0.02	-0.84	0.80	0.9594	0.17	-0.90	1.23	0.7463
	Rapamycin	20	-0.22	0.78	0.00	-0.74	0.73	0.9903	0.18	-0.76	1.12	0.6932
**Inflammasome (RNA) NLRP3**	Placebo	7	-0.48	0.31	-	-	-	-	-	-	-	-
	Rapamycin 1 mg/m²/d	8	0.29	0.95	0.77	-0.53	2.06	0.2317	1.08	-0.33	2.49	0.1256
	Rapamycin 2 mg/m²/d	11	0.29	1.63	0.77	-0.43	1.98	0.1982	1.35	-0.07	2.77	0.0619
	Rapamycin	19	0.29	1.35	0.77	-0.31	1.85	0.1531	1.21	-0.05	2.47	0.0590
**Inflammasome (RNA) PYCARD**	Placebo	7	0.00	0.46	-	-	-	-	-	-	-	-
	Rapamycin 1 mg/m²/d	8	0.00	0.43	0.01	-0.50	0.51	0.9762	-0.08	-0.62	0.46	0.7635
	Rapamycin 2 mg/m²/d	12	-0.06	0.51	-0.06	-0.53	0.40	0.7894	0.12	-0.43	0.66	0.6608
	Rapamycin	20	-0.04	0.47	-0.03	-0.45	0.39	0.8708	0.02	-0.47	0.51	0.9451
**TGF-B1**	Placebo	16	2818.74	27182.94	-	-	-	-	-	-	-	-
	Rapamycin 1 mg/m²/d	17	13767.69	26521.74	10948.95	-6332.68	28230.58	0.2086	13184.53	-6387.92	32756.98	0.1813
	Rapamycin 2 mg/m²/d	16	6517.51	19337.67	3698.76	-13842.76	21240.28	0.6732	6924.12	-12389.08	26237.32	0.4734
	Rapamycin	33	10252.45	23249.25	7433.71	-7625.93	22493.35	0.3258	9971.12	-6731.23	26673.46	0.2352
**IL-12**	Placebo	7	-42.66	64.25	-	-	-	-	-	-	-	-
	Rapamycin 1 mg/m²/d	10	-82.21	231.93	-39.55	-196.88	117.78	0.6080	66.25	-145.30	277.80	0.5200
	Rapamycin 2 mg/m²/d	9	-5.33	69.72	37.33	-123.56	198.22	0.6358	122.16	-77.51	321.84	0.2158
	Rapamycin	19	-45.79	174.96	-3.13	-144.48	138.21	0.9639	99.20	-85.57	283.96	0.2760
**IL-18**	Placebo	16	58.75	105.41	-	-	-	-	-	-	-	-
	Rapamycin 1 mg/m²/d	17	-49.05	120.91	-107.80	-176.70	-38.90	0.0029	-126.32	-197.62	-55.03	0.0009
	Rapamycin 2 mg/m²/d	16	-44.25	53.98	-103.00	-172.93	-33.06	0.0048	-87.33	-157.68	-16.98	0.0162
	Rapamycin	33	-46.72	93.17	-105.47	-165.07	-45.87	0.0009	-106.31	-167.73	-44.89	0.0011
**IL-6**	Placebo	1	-2.91	-	-	-	-	-	-	-	-	-
	Rapamycin 1 mg/m²/d	4	-3.56	4.81	-0.65	-12.85	11.56	0.8969	8.84	-30.69	48.37	0.4376
	Rapamycin 2 mg/m²/d	3	1.78	3.23	4.69	-7.91	17.30	0.3825	10.48	-37.78	58.74	0.4488
	Rapamycin	7	-1.27	4.81	1.64	-10.95	14.24	0.7606	8.05	-15.85	31.96	0.3623
**TNFa**	Placebo	10	2.30	4.11	-	-	-	-	-	-	-	-
	Rapamycin 1 mg/m²/d	8	-1.19	9.92	-3.49	-9.77	2.79	0.2603	-1.52	-8.98	5.95	0.6731
	Rapamycin 2 mg/m²/d	6	-0.33	1.43	-2.63	-9.46	4.21	0.4331	-0.50	-8.21	7.22	0.8931
	Rapamycin	14	-0.82	7.35	-3.12	-8.47	2.23	0.2389	-1.04	-7.34	5.26	0.7327

Mean absolute changes from baseline to week 18 are showed for each treatment group and comparison were performed using linear regression models that include indicator variables for treatment arms as the independent variables. For the comparison of Rapamycin and placebo arms, a P value of 0.05 or less was considered to indicate statistical significance. For the comparisons between Rapamycin 1 mg/m2/d or 2 mg/m2/d arms and the placebo arm, a P value of 0.025 or less was considered to indicate statistical significance to account for multiple arms comparison with the Bonferroni method. CIs were calculated based on the exact t distribution. All statistical tests were two-tailed.

The number of outcomes that were tested here was 11, and no correction was applied for multiple outcomes.

MD = mean difference; CI = confidence interval.

*Absolute change from baseline for inflammasome (RNA) AIM2, IL-1b (mRNA), IL-18 (mRNA), Inflammasome (RNA) NAIP, Inflammasome (RNA) NLRP3, Inflammasome (RNA) PYCARD were calculated considering normalized data at baseline (where all values were considered 1)

§Adjusted analyses for sex, ALSFRS-R slope at baseline, disease duration from onset to baseline and edaravone treatment.

Table S6. Changes from baseline to week 8-18-30-54 in serum pNfH in patients treated with rapamycin or placebo

Outcome	Arm	Absolute change from baseline			Unadjusted analysis				Adjusted analysis*			
		n	mean	SD	MD	95% CI		p	MD	95% CI		p
**Week8**	Placebo	19	-174.95	602.19	-	-	-	-	-	-	-	-
	Rapamycin 1 $mg/m^2/d$	20	66.45	334.88	241.40	-19.86	502.65	0.0695	244.92	-37.36	527.20	0.0876
	Rapamycin 2 $mg/m^2/d$	20	35.75	181.00	210.70	-50.56	471.95	0.1118	231.51	-43.55	506.56	0.0972
	Rapamycin	40	51.10	266.15	226.05	0.80	451.29	0.0492	237.84	-3.26	478.95	0.0531
**Week 18**	Placebo	17	-289.35	788.33	-	-	-	-	-	-	-	-
	Rapamycin 1 $mg/m^2/d$	18	138.50	441.16	427.85	63.13	792.58	0.0225	435.79	42.22	829.37	0.0308
	Rapamycin 2 $mg/m^2/d$	16	77.25	192.68	366.60	-9.03	742.24	0.0555	366.81	-33.72	767.33	0.0717
	Rapamycin	34	109.68	343.66	399.03	81.77	716.28	0.0148	402.53	61.44	743.62	0.0218
**Week 30**	Placebo	14	-482.29	927.19	-	-	-	-	-	-	-	-
	Rapamycin 1 $mg/m^2/d$	16	-112.44	577.43	369.85	-168.47	908.17	0.1728	145.11	-469.44	759.66	0.6352
	Rapamycin 2 $mg/m^2/d$	14	-85.29	654.81	397.00	-158.98	952.98	0.1569	352.55	-247.30	952.40	0.2413
	Rapamycin	30	-99.77	604.04	382.52	-87.61	852.65	0.1081	254.55	-278.03	787.13	0.3394
**Week54**	Placebo	8	-122.13	385.46	-	-	-	-	-	-	-	-
	Rapamycin 1 $mg/m^2/d$	11	-122.18	521.74	-0.06	-607.82	607.70	0.9998	-256.86	-976.84	463.12	0.4687
	Rapamycin 2 $mg/m^2/d$	12	-215.75	834.16	-93.63	-690.63	503.38	0.7504	-276.57	-919.73	366.58	0.3836
	Rapamycin	23	-171.00	688.42	-48.88	-576.75	479.00	0.8511	-269.30	-855.33	316.73	0.3530

Mean absolute changes from baseline to week 8-18-30-54 are showed for each treatment group and comparison were performed using linear regression models that include indicator variables for treatment arms as the independent variables. For the comparison of Rapamycin and placebo arms, a P value of 0.05 or less was considered to indicate statistical significance. For the comparisons between Rapamycin 1 mg/m2/d or 2 mg/m2/d arms and the placebo arm, a P value of 0.025 or less was considered to indicate statistical significance to account for multiple arms comparison with the Bonferroni method. CIs were calculated based on the exact t distribution. All statistical tests were two-tailed. MD = mean difference; CI = confidence interval.

*Adjusted analyses for sex, ALSFRS-R slope at baseline, disease duration from onset to baseline and edaravone treatment.

Table S7. Changes from baseline to week 8-18-30-54 in serum NfL in in patients treated with rapamycin or placebo

Outcome	Arm	Absolute change from baseline			Unadjusted analysis				Adjusted analysis*			
		**n**	**mean**	**SD**	**MD**	**95% CI**		**p**	**MD**	**95% CI**		**p**
Week 8	Placebo	18	-11.61	96.07	-	-	-	-	-	-	-	-
	Rapamycin 1 $mg/m^2/d$	20	8.25	20.54	19.86	-16.61	56.33	0.2799	27.14	-12.80	67.08	0.1785
	Rapamycin 2 $mg/m^2/d$	20	5.30	20.08	16.91	-19.56	53.38	0.3568	21.52	-17.69	60.73	0.2757
	Rapamycin	40	6.78	20.10	18.39	-13.18	49.96	0.2483	24.22	-10.16	58.60	0.1635
Week 18	Placebo	17	-25.76	93.46	-	-	-	-	-	-	-	-
	Rapamycin 1 $mg/m^2/d$	17	9.12	29.61	34.88	-5.13	74.90	0.0860	38.03	-5.80	81.86	0.0873
	Rapamycin 2 $mg/m^2/d$	16	9.19	16.91	34.95	-5.68	75.59	0.0901	32.99	-11.28	77.25	0.1402
	Rapamycin	33	9.15	23.93	34.92	0.47	69.36	0.0471	35.56	-2.19	73.31	0.0642
Week 30	Placebo	14	-33.21	101.57	-	-	-	-	-	-	-	-
	Rapamycin 1 $mg/m^2/d$	16	0.13	34.51	33.34	-13.49	80.17	0.1581	42.93	-12.12	97.98	0.1226
	Rapamycin 2 $mg/m^2/d$	14	-6.36	31.15	26.86	-21.51	75.22	0.2686	43.30	-10.43	97.03	0.1110
	Rapamycin	30	-2.90	32.58	30.31	-10.62	71.24	0.1425	43.13	-4.23	90.49	0.0731
Week 54	Placebo	9	-31.00	37.43	-	-	-	-	-	-	-	-
	Rapamycin 1 $mg/m^2/d$	11	-3.27	47.34	27.73	-12.71	68.16	0.1714	19.75	-31.10	70.59	0.4313
	Rapamycin 2 $mg/m^2/d$	12	3.00	45.22	34.00	-5.67	73.67	0.0902	25.98	-19.06	71.01	0.2460
	Rapamycin	23	0.00	45.29	31.00	-3.79	65.79	0.0788	23.70	-17.33	64.74	0.2458

Mean absolute changes from baseline to week 8-18-30-54 are showed for each treatment group and comparison were performed using linear regression models that include indicator variables for treatment arms as the independent variables. For the comparison of Rapamycin and placebo arms, a P value of 0.05 or less was considered to indicate statistical significance. For the comparisons between Rapamycin 1 mg/m2/d or 2 mg/m2/d arms and the placebo arm, a P value of 0.025 or less was considered to indicate statistical significance to account for multiple arms comparison with the Bonferroni method. CIs were calculated based on the exact t distribution. All statistical tests were two-tailed. MD = mean difference; CI = confidence interval.

Large SD in the placebo group are due to a single subject with a very large change in NfL from baseline.*Adjusted analyses for sex, ALSFRS-R slope at baseline, disease duration from onset to baseline and edaravone treatment.

Table S8. Changes from baseline to week 18 in CSF NfL and pNfH in patients treated with rapamycin or placebo

Outcome	Arm	Absolute change from baseline			Unadjusted analysis				Adjusted analysis*			
		n	mean	SD	MD	95% CI		p	MD	95% CI		p
pNfH CSF	Placebo	13	-860.00	1387.21	-	-	-	-	-	-	-	-
	Rapamycin 1 mg/m^2/d	9	-388.11	1524.80	471.89	-723.38	1667.15	0.4276	217.75	-1111.78	1547.29	0.7401
	Rapamycin 2 mg/m^2/d	14	260.29	1205.16	1120.29	58.61	2181.96	0.0392	1014.27	-124.32	2152.87	0.0788
	Rapamycin	23	6.57	1344.76	866.57	-92.39	1825.52	0.0750	725.45	-317.28	1768.18	0.1657
NfL CSF	Placebo	12	-4337.67	11194.97	-	-	-	-	-	-	-	-
	Rapamycin 1 mg/m^2/d	9	-523.33	1307.45	3814.33	-2175.56	9804.22	0.2039	4188.60	-2529.53	10906.72	0.2120
	Rapamycin 2 mg/m^2/d	14	1023.86	1540.09	5361.52	17.69	10705.36	0.0493	5097.90	-480.25	10676.04	0.0717
	Rapamycin	23	418.43	1618.41	4756.10	-23.53	9535.73	0.0511	4797.02	-268.41	9862.45	0.0626

Mean absolute changes from baseline to week 18 are showed for each treatment group and comparison were performed using linear regression models that include indicator variables for treatment arms as the independent variables. For the comparison of Rapamycin and placebo arms, a P value of 0.05 or less was considered to indicate statistical significance. For the comparisons between Rapamycin 1 mg/m2/d or 2 mg/m2/d arms and the placebo arm, a P value of 0.025 or less was considered to indicate statistical significance to account for multiple arms comparison with the Bonferroni method. CIs were calculated based on the exact t distribution. All statistical tests were two-tailed.MD = mean difference; CI = confidence interval.

Large SD in the placebo group are due to a single subject with a very large change in CSF NfL from baseline.

*Adjusted analyses for sex, ALSFRS-R slope at baseline, disease duration from onset to baseline and edaravone treatment.

Table S9. Changes from baseline to week 18 and week 30 in the phosphorylation of S6 ribosomal protein across treatment arms

Time points	Outcome	Arm	Absolute change from baseline			MD	95% CI		p
			n	mean	SD				
Week 18	PS6/CD8+ T CELLS	Placebo	4	1.35	10.56	-	-	-	-
		Rapamycin 1 mg/m²/d	3	0.37	4.50	-0.97	-20.11	18.16	0.9108
		Rapamycin 2 mg/m²/d	5	5.49	13.50	4.14	-12.67	20.94	0.5911
		Rapamycin	8	3.57	10.81	2.22	-12.43	16.87	0.7426
	PS6/TREG	Placebo	4	8.48	16.13	-	-	-	-
		Rapamycin 1 mg/m²/d	3	2.33	8.46	-6.15	-40.74	28.45	0.6972
		Rapamycin 2 mg/m²/d	5	8.62	25.90	0.14	-30.25	30.52	0.9920
		Rapamycin	8	6.26	20.36	-2.22	-28.40	23.96	0.8540
	PS6/CD4+ T CELLS	Placebo	4	5.73	15.37	-	-	-	-
		Rapamycin 1 mg/m²/d	3	-0.30	2.59	-6.03	-34.02	21.95	0.6375
		Rapamycin 2 mg/m²/d	5	5.12	20.24	-0.61	-25.19	23.97	0.9564
		Rapamycin	8	3.09	15.62	-2.64	-23.85	18.56	0.7869
Week 30	PS6/CD8+ T CELLS	Placebo	3	-1.09	16.64	-	-	-	-
		Rapamycin 1 mg/m²/d	3	-1.70	12.50	-0.61	-26.52	25.29	0.9577
		Rapamycin 2 mg/m²/d	5	-3.14	12.73	-2.05	-25.22	21.12	0.8433
		Rapamycin	8	-2.60	11.74	-1.51	-21.40	18.38	0.8672
	PS6/TREG	Placebo	3	16.37	35.45	-	-	-	-
		Rapamycin 1 mg/m²/d	3	-1.83	12.97	-18.20	-72.60	36.19	0.4624
		Rapamycin 2 mg/m²/d	5	4.30	30.93	-12.07	-60.72	36.58	0.5829
		Rapamycin	8	2.00	24.59	-14.37	-56.30	27.56	0.4581
	PS6/CD4+ T CELLS	Placebo	3	8.91	32.09	-	-	-	-
		Rapamycin 1 mg/m²/d	3	-4.23	13.15	-13.14	-57.68	31.39	0.5154
		Rapamycin 2 mg/m²/d	5	1.47	22.75	-7.44	-47.27	32.39	0.6780
		Rapamycin	8	-0.67	18.81	-9.58	-43.96	24.80	0.5442

Mean absolute changes from baseline to weeks 18 and 30 are showed for each treatment group and comparison were performed using linear regression models that include indicator variables for treatment arms as the independent variables. For the comparison of Rapamycin and placebo arms, a P value of 0.05 or less was considered to indicate statistical significance. For the comparisons between Rapamycin 1 mg/m2/d or 2 mg/m2/d arms and the placebo arm, a P value of 0.025 or less was considered to indicate statistical significance to account for multiple arms comparison with the Bonferroni method. CIs were calculated based on the exact t distribution. All statistical tests were two-tailed.MD = mean difference; CI = confidence interval.

Table S10. Absolute changes from baseline to week 8-18-30-54 in ALSFRS-R total score in patients treated with rapamycin or placebo

Mean absolute changes from baseline to week 8-18-30-54 are showed for each treatment group and comparison were performed using linear regression models that include indicator variables for treatment arms as the independent variables. For the comparison of Rapamycin and placebo arms, a P value of 0.05 or less was considered to indicate statistical significance. For the comparisons between Rapamycin 1 mg/m2/d or 2 mg/m2/d arms and the placebo arm, a P value of 0.025 or less was considered to indicate statistical significance to account for multiple arms comparison with the Bonferroni method. CIs were calculated based on the exact t distribution. All statistical tests were two-tailed.MD = mean difference; CI = confidence interval.
*Adjusted analyses for sex, ALSFRS-R slope at baseline, disease duration from onset to baseline and edaravone treatment.

**Week 8**	**Arm**	Absolute change from baseline			**Unadjusted analysis**				**Adjusted analysis***			
		**n**	**mean**	**SD**	**MD**	**95% CI[&]**		**p[&]**	MD	**95% CI[&]**		**p[&]**
**Week 8**	Placebo	20	-2.90	3.52	-	-	-	-	-	-	-	-
	Rapamycin 1 mg/m^2/d	20	-2.75	2.86	0.15	-1.70	2.00	0.8716	-0.28	-2.02	1.46	0.7472
	Rapamycin 2 mg/m^2/d	20	-2.95	2.24	-0.05	-1.90	1.80	0.9570	-0.53	-2.21	1.16	0.5329
	Rapamycin	40	-2.85	2.54	0.05	-1.54	1.64	0.9500	-0.41	-1.89	1.06	0.5767
**Week 18**	Placebo	20	-6.15	4.63	-	-	-	-	-	-	-	-
	Rapamycin 1 mg/m^2/d	18	-4.00	3.14	2.15	-0.67	4.97	0.1316	0.82	-1.39	3.03	0.4612
	Rapamycin 2 mg/m^2/d	18	-6.06	4.94	0.09	-2.72	2.91	0.9466	-0.88	-3.03	1.27	0.4156
	Rapamycin	36	-5.03	4.21	1.12	-1.32	3.56	0.3604	-0.08	-1.99	1.83	0.9347
**Week 30**	Placebo	18	-9.33	5.35	-	-	-	-	-	-	-	-
	Rapamycin 1 mg/m^2/d	16	-6.75	4.34	2.58	-1.13	6.30	0.1685	0.16	-2.48	2.80	0.9035
	Rapamycin 2 mg/m^2/d	15	-7.47	6.32	1.87	-1.92	5.65	0.3257	0.17	-2.42	2.76	0.8952
	Rapamycin	31	-7.10	5.31	2.24	-0.94	5.41	0.1630	0.17	-2.05	2.38	0.8811
**Week 54**	Placebo	14	-15.36	9.72	-	-	-	-	-	-	-	-
	Rapamycin 1 mg/m^2/d	13	-12.23	7.25	3.13	-4.11	10.36	0.3867	-1.32	-5.48	2.84	0.5229
	Rapamycin 2 mg/m^2/d	13	-12.85	10.49	2.51	-4.72	9.74	0.4861	-0.48	-4.27	3.32	0.8001
	Rapamycin	26	-12.54	8.84	2.82	-3.32	8.96	0.3585	-0.82	-4.21	2.57	0.6262

Table S11. Tracheostomy free survival (post hoc analysis, last observation set on 31st December 2021)

	Death or IV		No IV and alive		p-value
	n	%	n	%	
Placebo	13	61.9%	8	38.1%	-
Rapamycin 1 mg	10	47.6%	11	52.4%	0.6451
Rapamycin 2 mg	12	57.1%	9	42.9%	
Rapamycin	22	52.4%	20	47.6%	0.3559

Comparison between treatments arms was performed using the log-rank test.

Table S12. Respiratory function as measured by FVC (%) at different time points in patients treated with Rapamycin and placebo

Outcome	Arm	Absolute change from baseline			MD	95% CI		p
		n	mean	SD				
Week 18	Placebo	16	-6.82	17.31	-	-	-	-
	Rapamycin 1 mg/m^2/d	18	-3.62	17.21	3.20	-8.09	14.48	0.5714
	Rapamycin 2 mg/m^2/d	14	-11.39	14.12	-4.57	-15.86	6.71	0.4193
	Rapamycin	32	-7.50	15.98	-0.69	-10.44	9.07	0.8881
Week 30	Placebo	13	-12.41	21.71	-	-	-	-
	Rapamycin 1 mg/m^2/d	16	-15.67	16.74	-3.26	-17.30	10.78	0.6412
	Rapamycin 2 mg/m^2/d	13	-14.39	16.41	-1.98	-16.44	12.49	0.7836
	Rapamycin	29	-15.07	16.31	-2.66	-15.06	9.73	0.6666
Week 54	Placebo	9	-24.18	35.72	-	-	-	-
	Rapamycin 1 mg/m^2/d	8	-39.22	28.27	-15.04	-42.41	12.32	0.2693
	Rapamycin 2 mg/m^2/d	9	-26.38	21.34	-2.21	-27.80	23.39	0.8610
	Rapamycin	17	-31.89	24.75	-7.71	-30.82	15.41	0.5002

Mean absolute changes from baseline to week 18-30-54 are showed for each treatment group and comparison were performed using linear regression models that include indicator variables for treatment arms as the independent variables. For the comparison of Rapamycin and placebo arms, a P value of 0.05 or less was considered to indicate statistical significance. For the comparisons between Rapamycin 1 mg/m2/d or 2 mg/m2/d arms and the placebo arm, a P value of 0.025 or less was considered to indicate statistical significance to account for multiple arms comparison with the Bonferroni method. CIs were calculated based on the exact t distribution. All statistical tests were two-tailed.MD = mean difference; CI = confidence interval.

Notes: MD = mean difference; CI = confidence interval

Table S13. ALSAQ40 administered at different time points in patients treated with rapamycin and placebo

ALSAQ40 domains	Time points	Arm	Absolute change from baseline			Unadjusted analysis				Adjusted analysis*			
			n	mean	SD	MD	95% CI		p	MD	95% CI		p
**Total % score**	Week 8	Placebo	20	6.69	12.22	-	-	-	-	-	-	-	-
		Rapamycin 1 mg/m^2/d	20	4.41	10.29	-2.28	-9.18	4.62	0.5109	0.93	-6.32	8.18	0.7983
		Rapamycin 2 mg/m^2/d	20	5.31	10.05	-1.37	-8.27	5.53	0.6916	-0.77	-7.77	6.24	0.8272
		Rapamycin	40	4.86	10.05	-1.83	-7.75	4.10	0.5396	0.02	-6.11	6.16	0.9944
	Week 18	Placebo	20	13.97	12.17	-	-	-	-	-	-	-	-
		Rapamycin 1 mg/m^2/d	18	7.68	12.09	-6.29	-14.81	2.23	0.1445	-2.95	-11.75	5.84	0.5029
		Rapamycin 2 mg/m^2/d	17	10.96	14.96	-3.01	-11.66	5.65	0.4888	-1.82	-10.47	6.83	0.6742
		Rapamycin	35	9.27	13.46	-4.69	-12.01	2.62	0.2037	-2.37	-9.79	5.05	0.5244
	Week 30	Placebo	16	18.15	12.64	-	-	-	-	-	-	-	-
		Rapamycin 1 mg/m^2/d	16	13.11	13.83	-5.04	-14.89	4.81	0.3083	2.16	-7.29	11.61	0.6469
		Rapamycin 2 mg/m^2/d	15	7.46	14.99	-10.69	-20.71	-0.68	0.0370	-5.87	-15.19	3.45	0.2101
		Rapamycin	31	10.38	14.45	-7.77	-16.37	0.83	0.0753	-1.97	-10.32	6.37	0.6355
	Week 54	Placebo	12	27.01	24.33	-	-	-	-	-	-	-	-
		Rapamycin 1 mg/m^2/d	12	23.02	18.43	-3.99	-20.63	12.65	0.6291	7.28	-9.74	24.29	0.3893
		Rapamycin 2 mg/m^2/d	13	19.49	16.90	-7.52	-23.83	8.79	0.3555	-0.66	-15.69	14.38	0.9294
		Rapamycin	25	21.18	17.37	-5.83	-19.96	8.31	0.4083	2.15	-11.98	16.28	0.7582
**Physical mobility % score**	Week 8	Placebo	20	7.75	15.19	-	-	-	-	-	-	-	-
		Rapamycin 1 mg/m^2/d	20	1.63	12.93	-6.13	-15.12	2.87	0.1779	-1.68	-11.26	7.90	0.7267
		Rapamycin 2 mg/m^2/d	20	5.88	14.38	-1.88	-10.87	7.12	0.6778	-0.93	-10.18	8.33	0.8413
		Rapamycin	40	3.75	13.67	-4.00	-11.78	3.78	0.3075	-1.28	-9.37	6.82	0.7530
	Week 18	Placebo	20	16.50	18.43	-	-	-	-	-	-	-	-
		Rapamycin 1 mg/m^2/d	18	7.08	11.19	-9.42	-20.61	1.77	0.0973	-2.81	-14.33	8.71	0.6262
		Rapamycin 2 mg/m^2/d	17	17.21	20.52	0.71	-10.66	12.07	0.9013	2.41	-8.91	13.74	0.6701
		Rapamycin	35	12.00	16.94	-4.50	-14.33	5.33	0.3629	-0.11	-9.90	9.68	0.9818
	Week 30	Placebo	16	23.28	17.17	-	-	-	-	-	-	-	-
		Rapamycin 1 mg/m^2/d	16	15.63	18.02	-7.66	-21.61	6.30	0.2748	2.54	-11.12	16.19	0.7091
		Rapamycin 2 mg/m^2/d	15	20.67	23.27	-2.61	-16.80	11.57	0.7120	1.32	-12.14	14.78	0.8438
		Rapamycin	31	18.06	20.53	-5.22	-17.29	6.86	0.3888	1.91	-9.72	13.54	0.7416
	Week 54	Placebo	12	36.67	26.08	-	-	-	-	-	-	-	-
		Rapamycin 1 mg/m^2/d	12	24.17	24.69	-12.50	-34.47	9.47	0.2556	6.82	-18.26	31.90	0.5829
		Rapamycin 2 mg/m^2/d	13	30.58	28.36	-6.09	-27.63	15.45	0.5694	3.13	-19.04	25.29	0.7752
		Rapamycin	25	27.50	26.31	-9.17	-27.87	9.54	0.3266	4.43	-16.03	24.90	0.6616
**ADL and independence % score**	Week 8	Placebo	20	5.88	12.88	-	-	-	-	-	-	-	-
		Rapamycin 1 mg/m^2/d	20	9.50	15.04	3.63	-4.72	11.97	0.3881	5.18	-3.88	14.24	0.2567
		Rapamycin 2 mg/m^2/d	20	9.00	11.37	3.13	-5.22	11.47	0.4565	2.55	-6.21	11.30	0.5616
		Rapamycin	40	9.25	13.16	3.38	-3.79	10.54	0.3496	3.77	-3.91	11.46	0.3292
	Week 18	Placebo	20	17.25	16.93	-	-	-	-	-	-	-	-
		Rapamycin 1 mg/m^2/d	18	13.61	15.54	-3.64	-14.54	7.26	0.5059	-2.31	-14.34	9.72	0.7012
		Rapamycin 2 mg/m^2/d	17	15.88	17.65	-1.37	-12.44	9.70	0.8052	0.37	-11.46	12.20	0.9497
		Rapamycin	35	14.71	16.39	-2.54	-11.86	6.79	0.5878	-0.92	-11.09	9.24	0.8557
	Week 30	Placebo	16	21.88	15.04	-	-	-	-	-	-	-	-
		Rapamycin 1 mg/m^2/d	16	23.28	18.50	1.41	-11.68	14.49	0.8295	7.65	-6.56	21.86	0.2831
		Rapamycin 2 mg/m^2/d	15	16.83	21.22	-5.04	-18.34	8.26	0.4489	0.37	-13.63	14.38	0.9571
		Rapamycin	31	20.16	19.80	-1.71	-13.09	9.66	0.7630	3.91	-8.36	16.17	0.5236
	Week 54	Placebo	12	26.25	16.84	-	-	-	-	-	-	-	-

		Rapamycin 1 mg/m^2/d	12	35.00	26.37	8.75	-11.12	28.62	0.3771	18.63	-5.28	42.54	0.1220
		Rapamycin 2 mg/m^2/d	13	30.96	26.97	4.71	-14.77	24.20	0.6263	14.52	-6.61	35.65	0.1707
		Rapamycin	25	32.90	26.21	6.65	-10.22	23.52	0.4290	15.98	-3.55	35.50	0.1052
**Eating and drinking % score**	Week 8	Placebo	20	5.42	28.78	-	-	-	-	-	-	-	-
		Rapamycin 1 mg/m^2/d	20	1.25	17.99	-4.17	-17.64	9.31	0.5382	-1.13	-15.99	13.73	0.8797
		Rapamycin 2 mg/m^2/d	20	7.08	14.38	1.67	-11.81	15.14	0.8053	3.65	-10.70	18.01	0.6119
		Rapamycin	40	4.17	16.34	-1.25	-12.89	10.39	0.8306	1.43	-11.18	14.04	0.8208
	Week 18	Placebo	20	11.67	27.49	-	-	-	-	-	-	-	-
		Rapamycin 1 mg/m^2/d	18	1.39	22.91	-10.28	-26.11	5.56	0.1985	-5.73	-22.24	10.78	0.4887
		Rapamycin 2 mg/m^2/d	17	15.20	21.50	3.53	-12.55	19.61	0.6614	5.93	-10.30	22.17	0.4660
		Rapamycin	35	8.10	23.00	-3.57	-17.46	10.32	0.6082	0.29	-13.91	14.49	0.9672
	Week 30	Placebo	16	14.06	28.17	-	-	-	-	-	-	-	-
		Rapamycin 1 mg/m^2/d	16	3.65	23.17	-10.42	-26.39	5.55	0.1955	-2.06	-18.21	14.09	0.7979
		Rapamycin 2 mg/m^2/d	15	6.11	12.39	-7.95	-24.19	8.28	0.3290	-1.19	-17.11	14.72	0.8802
		Rapamycin	31	4.84	18.48	-9.22	-22.98	4.53	0.1836	-1.61	-15.36	12.13	0.8137
	Week 54	Placebo	12	31.94	43.20	-	-	-	-	-	-	-	-
		Rapamycin 1 mg/m^2/d	12	10.42	26.38	-21.53	-48.18	5.12	0.1099	-14.88	-41.42	11.66	0.2612
		Rapamycin 2 mg/m^2/d	13	21.15	23.96	-10.79	-36.92	15.34	0.4072	-7.05	-30.50	16.41	0.5442
		Rapamycin	25	16.00	25.22	-15.94	-38.75	6.86	0.1646	-9.82	-31.60	11.96	0.3650
**Communication % score**	Week 8	Placebo	20	7.14	14.43	-	-	-	-	-	-	-	-
		Rapamycin 1 mg/m^2/d	20	4.29	15.05	-2.86	-11.90	6.18	0.5293	2.53	-6.12	11.18	0.5603
		Rapamycin 2 mg/m^2/d	20	2.86	13.29	-4.29	-13.32	4.75	0.3464	-2.95	-11.30	5.41	0.4823
		Rapamycin	40	3.57	14.03	-3.57	-11.33	4.19	0.3609	-0.40	-7.83	7.02	0.9139
	Week 18	Placebo	20	12.68	15.44	-	-	-	-	-	-	-	-
		Rapamycin 1 mg/m^2/d	18	7.14	18.82	-5.54	-17.23	6.16	0.3467	-2.38	-14.69	9.93	0.6987
		Rapamycin 2 mg/m^2/d	17	6.09	19.67	-6.59	-18.46	5.29	0.2709	-5.45	-17.55	6.66	0.3701
		Rapamycin	35	6.63	18.96	-6.05	-16.04	3.95	0.2304	-3.96	-14.37	6.44	0.4474
	Week 30	Placebo	16	16.07	23.62	-	-	-	-	-	-	-	-
		Rapamycin 1 mg/m^2/d	16	13.17	22.39	-2.90	-19.06	13.25	0.7191	3.07	-14.12	20.25	0.7200
		Rapamycin 2 mg/m^2/d	15	-0.48	21.93	-16.55	-32.97	-0.13	0.0483	-9.84	-26.78	7.10	0.2475
		Rapamycin	31	6.57	22.87	-9.50	-23.84	4.83	0.1885	-3.57	-18.63	11.49	0.6345
	Week 54	Placebo	12	25.60	40.77	-	-	-	-	-	-	-	-
		Rapamycin 1 mg/m^2/d	12	29.46	26.56	3.87	-22.28	30.01	0.7654	14.59	-15.14	44.33	0.3241
		Rapamycin 2 mg/m^2/d	13	12.64	25.36	-12.96	-38.60	12.68	0.3116	-7.61	-33.88	18.67	0.5588
		Rapamycin	25	20.71	26.81	-4.88	-27.60	17.84	0.6654	0.25	-25.17	25.68	0.9839
**Emotional functioning % score**	Week 8	Placebo	20	7.25	13.93	-	-	-	-	-	-	-	-
		Rapamycin 1 mg/m^2/d	20	5.38	10.89	-1.88	-10.60	6.85	0.6685	-0.26	-9.69	9.16	0.9556
		Rapamycin 2 mg/m^2/d	20	1.75	16.02	-5.50	-14.22	3.22	0.2119	-6.16	-15.26	2.95	0.1809
		Rapamycin	40	3.56	13.64	-3.69	-11.22	3.84	0.3311	-3.42	-11.50	4.67	0.4007
	Week 18	Placebo	20	11.75	14.12	-	-	-	-	-	-	-	-
		Rapamycin 1 mg/m^2/d	18	9.17	11.08	-2.58	-11.86	6.69	0.5787	-1.53	-11.69	8.62	0.7629
		Rapamycin 2 mg/m^2/d	17	0.44	17.05	-11.31	-20.73	-1.89	0.0196	-12.37	-22.35	-2.39	0.0162
		Rapamycin	35	4.93	14.76	-6.82	-14.99	1.35	0.0999	-7.13	-16.08	1.82	0.1159
	Week 30	Placebo	16	15.47	9.84	-	-	-	-	-	-	-	-
		Rapamycin 1 mg/m^2/d	16	9.84	12.43	-5.63	-15.87	4.62	0.2747	-0.41	-11.61	10.80	0.9420
		Rapamycin 2 mg/m^2/d	15	-5.83	19.52	-21.30	-31.72	-10.88	0.0002	-20.03	-31.08	-8.98	0.0007
		Rapamycin	31	2.26	17.85	-13.21	-22.91	-3.51	0.0087	-10.50	-21.48	0.47	0.0602
	Week 54	Placebo	12	14.58	20.19	-	-	-	-	-	-	-	-
		Rapamycin 1 mg/m^2/d	12	16.04	13.63	1.46	-12.21	15.13	0.8296	11.22	-3.78	26.21	0.1370
		Rapamycin 2 mg/m^2/d	13	2.12	14.99	-12.47	-25.87	0.93	0.0672	-6.28	-19.54	6.97	0.3405
		Rapamycin	25	8.80	15.75	-5.78	-18.09	6.53	0.3468	-0.09	-13.75	13.58	0.9897

Mean absolute changes from baseline to week 8-18-30-54 are showed for each treatment group and comparison were performed using linear regression models that include indicator variables for treatment arms as the independent variables. For the comparison of Rapamycin and placebo arms, a P value of 0.05 or less was considered to indicate statistical significance. For the comparisons between Rapamycin 1 mg/m2/d or 2 mg/m2/d arms and the placebo arm, a P value of 0.025 or less was considered to indicate statistical significance to account for multiple arms comparison with the Bonferroni method. CIs were calculated based on the exact t distribution. All statistical tests were two-tailed.MD = mean difference; CI = confidence interval.

*Adjusted analyses for sex, ALSFRS-R slope at baseline, disease duration from onset to baseline and edaravone treatment.

Table S14. Changes in clinical outcome measures during and after treatment across treatment arms

Average monthly variations during and after treatment for the placebo group, as well as the comparisons between arms, are shown. Comparisons were performed using segmented repeated measures linear mixed models. Two segments of time were analyzed: during the treatment (after baseline and up to week 18), and after the treatment (after week 18). The dependent variables were the raw measurements of the outcomes, whereas the independent variables were: arm, time (months from baseline) x period (during or after treatment) interaction, and arm x time x period interaction. A random intercept term was also used to account for repeated measurements over the same individual, as well as a random slope term was used to account for individual linear variations over time. Random intercept and random slope terms were kept in the model if they improved the overall goodness-of-fit of the model. For the comparison of Rapamycin and placebo arms, a P value of 0.05 or less was considered to indicate statistical significance. For the comparisons between Rapamycin 1 mg/m2/d or 2 mg/m2/d arms and the placebo arm, a P value of 0.025 or less was considered to indicate statistical significance to account for multiple arms comparison with the Bonferroni method. CIs were calculated based on the exact t distribution using the Satterthwaite's method for degrees of freedom. All statistical tests were two-tailed.

Outcome	Period		Arm	MD	95% CI		p
FVC [1]	During treatment	Monthly variation	Placebo	-2.48	-4.13	-0.82	0.0036
	After treatment		Placebo	-2.98	-4.12	-1.85	0.0000
	During treatment	Comparison with placebo (monthly variation)	Rapamycin 1 mg	0.66	-2.08	3.40	1.0000
			Rapamycin 2 mg	-0.50	-3.23	2.24	1.0000
			Rapamycin	0.10	-1.96	2.16	0.9244
	After treatment		Rapamycin 1 mg	-0.01	-1.90	1.88	1.0000
			Rapamycin 2 mg	0.66	-1.22	2.55	0.8367
			Rapamycin	0.33	-1.07	1.74	0.6351
MRC Total % [1]	During treatment	Monthly variation	Placebo	-2.31	-3.30	-1.32	0.0000
	After treatment		Placebo	-2.74	-3.57	-1.90	0.0000
	During treatment	Comparison with placebo (monthly variation)	Rapamycin 1 mg	-0.09	-1.71	1.53	1.0000
			Rapamycin 2 mg	-0.19	-1.81	1.43	1.0000
			Rapamycin	-0.14	-1.35	1.07	0.8213
	After treatment		Rapamycin 1 mg	0.29	-1.09	1.67	1.0000
			Rapamycin 2 mg	0.61	-0.78	2.00	0.6247
			Rapamycin	0.44	-0.58	1.46	0.3868
ALSAQ-40 total % score [1]	During treatment	Monthly variation	Placebo	3.18	2.00	4.35	0.0000
	After treatment		Placebo	2.70	1.80	3.60	0.0000
	During treatment	Comparison with placebo (monthly variation)	Rapamycin 1 mg	-1.15	-3.08	0.79	0.3638
			Rapamycin 2 mg	-0.36	-2.32	1.60	1.0000
			Rapamycin	-0.77	-2.23	0.69	0.3001
	After treatment		Rapamycin 1 mg	-0.44	-1.91	1.03	0.9819
			Rapamycin 2 mg	-0.76	-2.25	0.74	0.4947
			Rapamycin	-0.59	-1.69	0.51	0.2847
ALSAQ-40 physical mobility % score [1]	During treatment	Monthly variation	Placebo	3.78	2.23	5.33	0.0000
	After treatment		Placebo	3.31	2.18	4.44	0.0000
	During treatment	Comparison with placebo (monthly variation)	Rapamycin 1 mg	-2.15	-4.70	0.40	0.1171
			Rapamycin 2 mg	0.37	-2.22	2.95	1.0000
			Rapamycin	-0.92	-2.86	1.01	0.3480
	After treatment		Rapamycin 1 mg	-1.27	-3.12	0.58	0.2371
			Rapamycin 2 mg	0.02	-1.85	1.89	1.0000
			Rapamycin	-0.64	-2.04	0.77	0.3659
ALSAQ-40 ADL and independence % score [1]	During treatment	Monthly variation	Placebo	4.22	2.61	5.84	0.0000
	After treatment		Placebo	3.44	2.26	4.62	0.0000
	During treatment	Comparison with placebo (monthly variation)	Rapamycin 1 mg	-0.59	-3.24	2.06	1.0000
			Rapamycin 2 mg	-0.55	-3.24	2.14	1.0000
			Rapamycin	-0.58	-2.57	1.42	0.5690
	After treatment		Rapamycin 1 mg	-0.28	-2.21	1.64	1.0000
			Rapamycin 2 mg	-0.73	-2.69	1.23	0.7806
			Rapamycin	-0.50	-1.94	0.94	0.4885
ALSAQ-40 eating and drinking % score [1]	During treatment	Monthly variation	Placebo	2.78	0.84	4.73	0.0052
	After treatment		Placebo	2.11	0.74	3.47	0.0032
	During treatment	Comparison with placebo (monthly variation)	Rapamycin 1 mg	-2.12	-5.31	1.08	0.2727
			Rapamycin 2 mg	0.44	-2.80	3.68	1.0000
			Rapamycin	-0.87	-3.28	1.54	0.4764
	After treatment		Rapamycin 1 mg	-0.57	-2.79	1.65	1.0000

Outcome	Period		Arm	MD	95% CI		p
			Rapamycin 2 mg	-0.48	-2.74	1.77	1.0000
			Rapamycin	-0.53	-2.19	1.12	0.5187
ALSAQ-40 communication % score (1)	During treatment	Monthly variation	Placebo	2.76	0.91	4.61	0.0037
	After treatment		Placebo	2.29	0.94	3.64	0.0013
	During treatment	Comparison with placebo (monthly variation)	Rapamycin 1 mg	-0.91	-3.95	2.13	1.0000
			Rapamycin 2 mg	-0.43	-3.51	2.65	1.0000
			Rapamycin	-0.68	-3.00	1.64	0.5636
	After treatment		Rapamycin 1 mg	0.28	-1.92	2.48	1.0000
			Rapamycin 2 mg	-1.16	-3.39	1.08	0.4738
			Rapamycin	-0.41	-2.10	1.27	0.6252
ALSAQ-40 emotional functioning % score (1)	During treatment	Monthly variation	Placebo	2.30	0.88	3.72	0.0016
	After treatment		Placebo	2.03	1.20	2.85	0.0000
	During treatment	Comparison with placebo (monthly variation)	Rapamycin 1 mg	-0.02	-2.35	2.31	1.0000
			Rapamycin 2 mg	-1.68	-4.05	0.68	0.2200
			Rapamycin	-0.84	-2.61	0.93	0.3511
	After treatment		Rapamycin 1 mg	-0.49	-1.83	0.86	0.8101
			Rapamycin 2 mg	-1.49	-2.85	-0.12	0.0300
			Rapamycin	-0.99	-2.02	0.05	0.0610

MD = mean difference; CI = confidence interval.(1) = model with random intercept and random slope.

Table S15: Correlations among clinical outcome measures.

Pearson’s linear correlation coefficients are reported. Confidence intervals and p-values were calculated based on the exact t distribution. All statistical tests were two-tailed.

Arm	Outcome measure		Pearson’s correlation	95% CI		p
Placebo	ALSFRS-R	FVC	0.52	0.07	0.79	0.0275
Rapamycin	ALSFRS-R	FVC	0.30	-0.05	0.59	0.0958
Rapamycin 1 mg	ALSFRS-R	FVC	0.21	-0.32	0.64	0.4283
Rapamycin 2 mg	ALSFRS-R	FVC	0.29	-0.24	0.69	0.2711
All patients	ALSFRS-R	FVC	0.38	0.11	0.59	0.0066
Placebo	ALSFRS-R	ALSAQ-40	-0.43	-0.73	0.02	0.0586
Rapamycin	ALSFRS-R	ALSAQ-40	-0.51	-0.72	-0.21	0.0018
Rapamycin 1 mg	ALSFRS-R	ALSAQ-40	-0.41	-0.74	0.07	0.0914
Rapamycin 2 mg	ALSFRS-R	ALSAQ-40	-0.55	-0.82	-0.09	0.0220
All patients	ALSFRS-R	ALSAQ-40	-0.49	-0.67	-0.26	0.0001
Placebo	FVC	ALSAQ-40	-0.33	-0.69	0.16	0.1787
Rapamycin	FVC	ALSAQ-40	-0.19	-0.50	0.17	0.3106
Rapamycin 1 mg	FVC	ALSAQ-40	-0.31	-0.70	0.22	0.2489
Rapamycin 2 mg	FVC	ALSAQ-40	-0.02	-0.51	0.48	0.9504
All patients	FVC	ALSAQ-40	-0.23	-0.48	0.05	0.1082

Table S16. Individuals with Adverse Events across different treatment arms

Time point	Treatment arm	AE		No AE		RR	95% CI	p
		n	%	n	%			
Week 18	Placebo	7	33.3%	14	66.7%	-	-	-
	Rapamycin 1 mg	8	38.1%	13	61.9%	1.14	0.51 - 2.58	0.7474
	Rapamycin 2 mg	9	42.9%	12	57.1%	1.29	0.59 - 2.81	0.5251
	Rapamycin	17	40.5%	25	59.5%	1.21	0.60 - 2.46	0.5821
Week 54	Placebo	11	52.4%	10	47.6%	-	-	-
	Rapamycin 1 mg	10	47.6%	11	52.4%	0.91	0.50 - 1.67	0.7576
	Rapamycin 2 mg	13	61.9%	8	38.1%	1.18	0.70 - 2.00	0.5329
	Rapamycin	23	54.8%	19	45.2%	1.05	0.64 - 1.71	0.8581

The unadjusted comparisons were carried out with a chi-square test without any correction. For the comparison of Rapamycin and placebo arms, a P value of 0.05 or less was considered to indicate statistical significance. For the comparisons between Rapamycin 1 mg/m2/d or 2 mg/m2/d arms and the placebo arm, a P value of 0.025 or less was considered to indicate statistical significance to account for multiple arms comparison with the Bonferroni method. All statistical tests were two-tailed.
RR = relative risk; CI = confidence interval
[&] The confidence intervals and p-values related to Rapamycin 1 mg/m2/d and 2 mg/m2/d arms take into account multiple arms comparison.

Table S17. Adverse Events across different treatment arms

		PLACEBO (n = 21)		Rapamycin 1 mg/m^2/d (n = 21)		Rapamycin 2 mg/m^2/d (n = 21)	
AE	n	23		19		27	
SAE	n % [a]	7	30.4%	2	10.5%	7	25.9%
Relationship with treatment (none/remote)	n % [a]	21	91.3%	17	81.0%	21	77.8%
AE leading to treatment discontinuation	n % [a]	1	4.3%	1	5.3%	2	7.4%

Notes: [a] = % of AEs

Table S18. Individuals with Serious Adverse Events across different treatment arms

Time point	Treatment arm	SAE		No SAE		RR	95% CI	p
		n	%	n	%			
Week 18	Placebo	1	4.8%	20	95.2%	-	-	-
	Rapamycin 1 mg	0	0.0%	21	100.0%	-	-	-
	Rapamycin 2 mg	2	9.5%	19	90.5%	2.00	0.20 - 20.41	0.5490
	Rapamycin	2	4.8%	40	95.2%	1.00	0.10 - 10.41	1.0000
Week 54	Placebo	4	19.0%	17	81.0%	-	-	-
	Rapamycin 1 mg	2	9.5%	19	90.5%	0.50	0.10 - 2.44	0.3778
	Rapamycin 2 mg	6	28.6%	15	71.4%	1.50	0.49 - 4.56	0.4687
	Rapamycin	8	19.0%	34	81.0%	1.00	0.34 - 2.94	1.0000

The unadjusted comparisons were carried out with a chi-square test without any correction. For the comparison of Rapamycin and placebo arms, a P value of 0.05 or less was considered to indicate statistical significance. For the comparisons between Rapamycin 1 mg/m2/d or 2 mg/m2/d arms and the placebo arm, a P value of 0.025 or less was considered to indicate statistical significance to account for multiple arms comparison with the Bonferroni method. All statistical tests were two-tailed. RR = relative risk; CI = confidence interval

Table S19. Analysis of drop out during the study treatment and follow up

		PLACEBO (n = 21)		Rapamycin 1 mg (n = 21)		Rapamycin 2 mg (n = 21)	
		n	%	n	%	n	%
Drop-out	Total	6	28.6%	8	38.1%	8	38.1%
	During treatment	1	4.8%	3	14.3%	3	14.3%
	During follow-up	5	23.8%	5	23.8%	5	23.8%
Reasons of drop-out							
AEs and SAEs		1	4.8%	1	4.8%	2	9.5%
Death		1	4.8%	2	9.5%	1	4.8%
Tracheostomy		0	0.0%	0	0.0%	0	0.0%
Consent withdrawal		4	19.1%	3	14.3%	3	14.3%
Other		2	9.5%	2	9.5%	2	9.5%
Protocol deviations	At week 18	1	4.8%	4	19.0%	4	19.0%
Drop-out	At week 18	1	4.8%	3	14.3%	3	14.3%
Compliance	< 80% of therapy at week 18	1	4.8%	3	14.3%	4	19.0%
Number of patients analysed in intention-to-treat analysis	week 8	20	95.2%	20	95.2%	20	95.2%
	week 18 (end of treatment)	20	95.2%	18	85.7%	18	85.7%
	week 24	20	95.2%	17	81.0%	17	81.0%
	week 30	18	85.7%	16	76.2%	15	71.4%
	week 42	16	76.2%	15	71.4%	13	61.9%
	week 54 (end of follow-up)	15	71.4%	13	61.9%	13	61.9%
Number of patients analysed in per-protocol analysis	week 8	20	95.2%	18	85.7%	17	81.0%
	week 18 (end of treatment)	20	95.2%	17	81.0%	17	81.0%
	week 24	20	95.2%	16	76.2%	17	81.0%
	week 30	18	85.7%	16	76.2%	15	71.4%
	week 42	16	76.2%	15	71.4%	13	61.9%
	week 54 (end of follow-up)	15	71.4%	13	61.9%	13	61.9%

Table S20. Trial Drug Adherence

Trial drug adherence was assessed by having participants return their empty and unused batch each clinic visit. Adherence was defined as taking more than 80% or less than 125% of anticipated trial drug as determined by tablets count.

			PLACEBO		Rapamycin 1 mg/m²/d		Rapamycin 2 mg/m²/d	
Compliance	% of therapy	mean sd	93.7	21.8	92.1	19.3	89.4	21.5
	< 80%	n %	0	0.0%	1	5.6%	1	5.6%
	≥ 80%	n %	20	100.0%	17	94.4%	17	94.4%
Rapamycin use								
Constant dosing	Week 0-18	n %	20	100.0%	10	55.6%	3	16.7%
At least one reduction*	Week 0-18	n %	0	0.0%	8	44.4%	15	83.3%

*Dose reduction might be due to plasma dosage overpassing therapeutic window (performed by a biologist not involved in patients care) or to possible intolerance/side effects (performed by clinicians who were blinded to treatment)

6. References

1. Zhao W, Beers DR, Appel SH. Immune-mediated mechanisms in the pathoprogression of amyotrophic lateral sclerosis. *J Neuroimmune Pharmacol* 2013;**8**:888–99.

2. Deora V, Lee JD, Albornoz EA et al. The microglial NLRP3 inflammasome is activated by amyotrophic lateral sclerosis proteins. *Glia* 2020, **68**,407–421.

3. Trias E, King PH, Si Y et al. Mast cells and neutrophils mediate peripheral motor pathway degeneration in ALS. *JCI Insight* 2018;**3**:e123249.

4. Pavlov VA, Chavan SS, Tracey KJ. Molecular and Functional Neuroscience in Immunity. *Annu Rev Immunol* 2018;**36**:783–812.

5. McCombe PA, Lee JD, Woodruff TM, Henderson RD. The peripheral immune system and amyotrophic lateral sclerosis. *Front Neurol* 2020;**11:** 279.

6. Henkel JS, Beers DR, Wen S et al. . Regulatory t-lymphocytes mediate amyotrophic lateral sclerosis progression and survival. *EMBO Mol Med* 2013;**5**:64–79.

7. Beers DR, Zhao W, Wang J et al. ALS patients' regulatory t-lymphocytes are dysfunctional, and correlate with disease progression rate and severity. *JCI Insight* 2017;**2**:e89530.

8. Sheean RK, McKay FC, Cretney E et al. Association of regulatory t-cell expansion with progression of amyotrophic lateral sclerosis: A study of humans and a transgenic mouse model. *JAMA Neurol* 2018;**75**:681–689.

9. Mandrioli J, Mediani L, Alberti S, Carra S. ALS and FTD: Where RNA metabolism meets protein quality control. *Semin Cell Dev Biol* 2020;**99**:183–192.

10. Ling SC, Polymenidou M, Cleveland DW. Converging mechanisms in ALS and FTD: disrupted RNA and protein homeostasis. *Neuron* 2013;**79**: 416–438.

11. Argueti-Ostrovsky S, Alfahel L, Kahn J, Israelson A. All Roads Lead to Rome: Different Molecular Players Converge to Common Toxic Pathways in Neurodegeneration. *Cells* 2021;**10**:2438.

12. Appel SH, Beers DR, Zhao W. Amyotrophic lateral sclerosis is a systemic disease: peripheral contributions to inflammation-mediated neurodegeneration. *Curr Opin Neurol* 2021;**34**:765–772.

13. Takahama M, Akira S, Saitoh T. Autophagy limits activation of the inflammasomes. *Immunol Rev* 2018;**281**:62–73.

14. Cadwell, K. Crosstalk between autophagy and inflammatory signalling pathways: Balancing defence and homeostasis. *Nat Rev Immunol* 2016;**16**:661–675.

15. Péladeau C, Sandhu JK. Aberrant NLRP3 Inflammasome Activation Ignites the Fire of Inflammation in Neuromuscular Diseases. *Int J Mol Sci* 2021;**22**:6068.

16. Querfurth H, Lee HK. Mammalian/mechanistic target of rapamycin (mTOR) complexes in neurodegeneration. *Mol Neurodegener* 2021;**16**:44.

17. Berger Z, Ravikumar B, Menzies FM, et al. Rapamycin alleviates toxicity of different aggregate-prone proteins. *Hum Mol Genet* 2006;**15**:433-42.

18. Barmada SJ, Serio A, Arjun A et al. Autophagy induction enhances TDP43 turnover and survival in neuronal ALS models. *Nat Chem Biol* 2014;**10**:677–85.

19. Lipton JO, Sahin M. The neurology of mTOR. *Neuron.* 2014;**84**:275-91.

20. Ravikumar B, Vacher C, Berger Z, et al. Inhibition of mTOR induces autophagy and reduces toxicity of polyglutamine expansions in fly and mouse models of Huntington disease. *Nat Genet* 2004;**36**:585-95.

21. Lin X, Han L, Weng J, Wang K, Chen T. Rapamycin inhibits proliferation and induces autophagy in human neuroblastoma cells. *Biosci Rep* 2018;**38**:BSR20181822.

22. Sotthibundhu A, McDonagh K, von Kriegsheim A et al. Rapamycin regulates autophagy and cell adhesion in induced pluripotent stem cells. *Stem Cell Res Ther* 2016;**7:**166.

23. Caccamo A, Majumder S, Deng JJ, Bai Y, Thornton FB, Oddo S. Rapamycin rescues TDP-43 mislocalization and the associated low molecular mass neurofilament instability. *J Biol Chem* 2009;**284**:27416-24.

24. Ryu HH, Jun MH, Min KJ, Jang DJ, Lee YS, Kim HK, Lee JA. Autophagy regulates amyotrophic lateral sclerosis-linked fused in sarcoma-positive stress granules in neurons. *Neurobiol Aging* 2014;**35**:2822-2831.

25. Madill M, McDonagh K, Ma J, et al. Amyotrophic lateral sclerosis patient iPSC-derived astrocytes impair autophagy via non-cell autonomous mechanisms. *Mol Brain* 2017;**10**:22.

26. Wang IF, Guo BS, Liu YC, et al. Autophagy activators rescue and alleviate pathogenesis of a mouse model with proteinopathies of the TAR DNA-binding protein 43. *Proc Natl Acad Sci USA* 2012;**109**:15024–9.

27. Lattante S, de Calbiac H, Le Ber I, et al. Sqstm1 knock-down causes a locomotor phenotype ameliorated by rapamycin in a zebrafish model of ALS/FTLD. *Hum Mol Genet* 2015;**24**:1682–90.

28. Cheng CW, Lin MJ, Shen CK. Rapamycin alleviates pathogenesis of a new Drosophila model of ALS-TDP. *J Neurogenet* 2015;**29**:59–68.

29. Deivasigamani S, Verma HK, Ueda R, et al. A genetic screen identifies Tor as an interactor of VAPB in a Drosophila model of amyotrophic lateral sclerosis. *Biol Open* 2014;**3**:1127–38.

30. Nalbandian A, Llewellyn KJ, Nguyen C, et al. Rapamycin and chloroquine: the in vitro and in vivo effects of autophagy-modifying drugs show promising results in valosin containing protein multisystem proteinopathy. *PLoS One* 2015;**10**:e0122888.

31. Sinclair LV, Finlay D, Feijoo C, et al. Phosphatidylinositol-3-OH kinase and nutrient-sensing mTOR pathways control T lymphocyte trafficking. *Nat Immunol* 2008;**9**:513-21.

32. Laberge RM, Sun Y, Orjalo AV, et al. MTOR regulates the pro-tumorigenic senescence-associated secretory phenotype by promoting IL1A translation. *Nat Cell Biol* 2015;**17**:1049-61.

33. Lau L, David G. Pro- and anti-tumorigenic functions of the senescence-associated secretory phenotype. *Expert Opin Ther Targets* 2019;**23**:1041-1051.

34. Orjalo AV, Bhaumik D, Gengler BK, Scott GK, Campisi J. Cell surface-bound IL-1alpha is an upstream regulator of the senescence-associated IL-6/IL-8 cytokine network. *Proc Natl Acad Sci U S A* 2009;**106**:17031-6.

35. Herranz N, Gallage S, Mellone M, et al. mTOR regulates MAPKAPK2 translation to control the senescence-associated secretory phenotype.*Nat Cell Biol*. 2015;17:1205-17.

36. Lamming DW, Mihaylova MM, Katajisto P, et al. Depletion of Rictor, an essential protein component of mTORC2, decreases male lifespan. *Aging Cell* 2014;**13**:911-7.

37. Thonhoff JR, Beers DR, Zhao W, et al. Expanded autologous regulatory T-lymphocyte infusions in ALS: A phase I, first-in-human study. *Neurol Neuroimmunol Neuroinflamm* 2018;**5**:e465.

38. Beers DR, Thonhoff JR, Faridar A, et al. Tregs Attenuate Peripheral Oxidative Stress and Acute Phase Proteins in ALS. *Ann Neurol* 2022;**92**:195-200.

39. Sheean RK, McKay FC, Cretney E, al. Association of Regulatory T-Cell Expansion With Progression of Amyotrophic Lateral Sclerosis: A Study of Humans and a Transgenic Mouse Model. *JAMA Neurol* 2018;**75**:681–689.

40. Cossarizza A, Chang HD, Radbruch A, et al. Guidelines for the use of flow cytometry and cell sorting in immunological studies (third edition). *Eur J Immunol* 2021;**51**:2708–3145.

41. De Biasi S, Lo Tartaro D, Meschiari M, et al. Expansion of plasmablasts and loss of memory B cells in peripheral blood from COVID-19 patients with pneumonia. *Eur J Immunol* 2020;**50**:1283–129442.

42. Zhang Q, Chikina M, Szymczak-Workman AL, et al. LAG3 limits regulatory T cell proliferation and function in autoimmune diabetes. *Sci Immunol* 2017;2:eaah456943.

43. Fan X, Moltedo B, Mendoza A, et al. CD49b defines functionally mature Treg cells that survey skin and vascular tissues. *J Exp Med* 2018;**215**:2796-2814. doi: 10.1084/jem.20181442

44. Chaudhry A, Samstein RM, Treuting P, et al. Interleukin-10 signaling in regulatory T cells is required for suppression of Th17 cell-mediated inflammation. *Immunity* 2011;**34**:566-78. doi: 10.1016/j.immuni.2011.03.018.

45. Cedarbaum JM, Stambler N, Malta E et al. The ALSFRS-R: a revised ALS functional rating scale that incorporates assessments of respiratory function. BDNF ALS Study Group (Phase III). *J Neurol Sci* 1999;**169**:13–21.

7. Abbreviations List

ADL	Activity Daily Living
AE	Adverse Event
AIM2	Absent in melanoma 2
ALS	Amyotrophic lateral sclerosis
ALSAQ40	The 40 item Amyotrophic Lateral Sclerosis Assessment Questionnaire
ALSFRS-R	Amyotrophic Lateral Sclerosis Functional Rating Scale Revised
APC	Antigen-presenting cell
BMI	Body mass index
BSA	Body surface area
CBC	Complete blood count
CCR	C-C chemokine receptor
CD	Cluster of differentiation
CK	Creatine kinase
CI	Confidence interval
CM	Central Memory
CNS	Central Nervous System
CSF	Cerebrospinal fluid
CXCR4	C-X-C chemokine receptor type 4
DMSO	Dimethylsulfoxide
ECG	Electrocardiogram
EDTA	Ethylenediamine tetraacetic acid
EM	Effector Memory
EMRA	Terminal differentiated Effector Memory

**FACS**	**Flow Cytometry Staining Buffer**
**FBS**	**Fetal bovine serum**
**FMO**	**Fluorencence Minus One**
**FoxP3**	**Forkhead box P3**
**FSC-A**	**Forward Scatter Area - Area**
**FSC-H**	**Forward Scatter Area - Heigh**
**FVC**	**Forced vital capacity**
**HLA**	**Human leukocyte antigen**
**IFN**	**Interferon**
**Ig**	**Immunoglobulin**
**IL**	**Interleukin**
**ITT**	**Intent-to-treat**
**IV**	**Invasive Ventilation**
**mAb**	**Monoclonal Antibody**
**MD**	**Mean Difference**
**MedDRA**	**Medical Dictionary for Regulatory Activities**
**MFI**	**Median Fluorencent Intensity**
**MN**	**Motorneuron**
**MRC**	**Medical Research Council**
**mRNA**	**Messenger RNA**
**mTOR**	**Mechanistic target of rapamycin**
**mTORC**	**Mechanistic target of rapamycin complex 1**
**NAIP**	**Neuronal apoptosis inhibitory protein**
**NK**	**Natural Killer**
**NF**	**Neurofilament**

**NLRP3**	**NLR family pyrin domain containing 3**
**NfL**	**Neurofilament light chain**
**OR**	**Odds ratio**
**PBMC**	**Peripheral blood mononuclear cell**
**PCR**	**Polymerase chain reaction**
**PD-1**	**Programmed cell death protein 1**
**pNfH**	**phosphorylated neurofilament heavy chain**
**PYCARD**	**Apoptosis-associated speck-like protein containing a CARD.**
**RR**	**Relative risk**
**SAE**	**Severe Adverse Event**
**SD**	**Standard Deviation**
**SOD1**	**Superoxide dismutase 1**
**SSC-H**	**Side Scatter- Heigh**
**TDP43**	**Transactive response DNA binding protein 43 kDa**
**TGF**	**Transforming growth factor**
**TH**	**T Hepler**
**TNF**	**Tumor necrosis factor**
**Tregs**	**Regulatory T Cells**

UNIMORE
UNIVERSITÀ DEGLI STUDI DI MODENA E REGGIO EMILIA

Dipartimento Integrato di Neuroscienze
Unità Operativa di Neurologia
Ospedale Civile Sant'Agostino Estense

SERVIZIO SANITARIO REGIONALE
EMILIA-ROMAGNA
Azienda Ospedaliero - Universitaria di Modena

Via Giardini, 1355
Baggiovara
41126 Modena
tel.: 059396111
fax.: 0593962409

Clinical Study Protocol

RAPAMYCIN (SIROLIMUS) TREATMENT FOR AMYOTROPHIC LATERAL SCLEROSIS

**Protocol Acronym:**	**RAP-ALS**
**Title:**	**Rapamycin (Sirolimus) treatment for amyotrophic lateral sclerosis**
**EUDRACT:**	**2016-002399-28**
**Phase of development:**	**Phase 2**
**Study design:**	**Multicenter, randomised, double-blind, placebo-controlled, phase 2 study to compare biological effects, safety and efficacy of Rapamycin in combination with riluzole versus placebo in combination with riluzole in the treatment of patients with Amyotrophic Lateral Sclerosis (ALS)**
**Diagnosis:**	**Patients with definite or probable ALS**
**Study treatment:**	**Rapamycin 1 mg tablets**
**Comparator product:**	**Placebo, matching 1 mg tablets**
**Associated product:**	**Riluzole 50 mg tablets**
**Duration of treatment:**	**18 weeks**
**Coordinating investigator:**	**Dr Jessica Mandrioli, Nuovo Ospedale Civile S. Agostino Estense, Via P. Giardini 1355, 41126 Modena, Italy**
**Sponsor:**	**Azienda Ospedaliero Universitaria di Modena**
**Pharmacovigilance:**	**Phast consulting s.r.l.**
**CRO**	**High Research s.r.l.**

Study Synopsis

**Study Title**	**RAPAMYCIN (SIROLIMUS) TREATMENT FOR AMYOTROPHIC LATERAL SCLEROSIS**
**Protocol acronym**	RAP-ALS
**PI Name**	JESSICA MANDRIOLI
**Principal Institution**	DEPARTMENT OF NEUROSCIENCE, NUOVO OSPEDALE CIVILE S. AGOSTINO-ESTENSE DI MODENA, AZIENDA OSPEALIERO UNIVERSITARIA – MODENA
**Partners and Participating Centres**	1) PROF. ANDREA COSSARIZZA, UNIVERSITY OF MODENA AND REGGIO EMILIA, MODENA<br>2) PROF. ROBERTO D'AMICO, UNIVERSITY OF MODENA AND REGGIO EMILIA, MODENA<br>3) DR. LETIZIA MAZZINI, A.O.U. MAGGIORE DELLA CARITA', NOVARA<br>4) DR. CLAUDIA CAPONNETTO, IRCCS A.O.U. S.MARTINO IST, GENOVA<br>5) PROF. ADRIANO CHIÒ, UNIVERSITY OF TORINO, TORINO<br>6) DR. ELEONORA DALLA BELLA, IRCCS ISTITUTO CARLO BESTA, MILANO<br>7) DR. CHRISTIAN LUNETTA, CENTRO CLINICO NEMO, FONDAZIONE SERENA ONLUS, MILANO<br>8) DR. KALLIOPI MARINOU, IRCCS FONDAZIONE SALVATORE MAUGERI, MILANO<br>9) DR. GIANNI SORARU', UNIVERSITY OF PADOVA, PADOVA
**Study Aims and Objectives**	**Objectives, primary aim:** to assess whether RAPAMYCIN administration increases Tregs number in treated patients compared to control arm; **secondary aims:** To assess safety and tolerability of RAPAMYCIN in ALS patients; to assess the minimum dosage to have RAPAMYCIN in CSF; to assess changes in immunological (activation and homing of T,B,NK cell subpopulations) and inflammatory markers, and on mTOR downstream pathway (S6RP phosphorylation); to assess clinical activity (ALSFRS-R, survival, FVC) and effect on quality of life (ALSAQ40).
**Study Rationale**	Misfolded aggregated proteins significantly contribute to ALS hence representing therapeutic targets to modify disease expression. Rapamycin (R) inhibits mechanistic target of R (mTOR) pathway and enhances autophagy with demonstrated beneficial effects in neurodegeneration. In two cell line models, R reduced TDP43 accumulation and restored TDP43 localization. R improved phenotype in SQSTM1 zebrafish model, and in the TDP43 mouse model R rescued memory and motor deficiencies, reducing neuronal loss and TDP43 inclusions by enhancing autophagy. Therefore, R accelerates the clearance of abnormally accumulated proteins, and may have beneficial effects on ALS.<br>R also expands regulatory T lymphocytes (Treg) that dampen immune responses: it has been shown that increased Treg levels are associated with slow ALS progression. Treg passive transfer into ALS mice prolonged survival, and FoxP3 (a specific Treg marker) mRNA in mSOD1 mice spinal cord decreased with disease progression. Based on these premises, we intend to perform the first human study with R in ALS carrying the double potential effect of enhancing TDP43 autophagy and expanding Tregs. R has never been used in ALS patients and its side effects in this fragile population are unknown. Moreover the best dosage, the capability of passing the blood brain barrier and the effects of the drug on available biomarkers have never been assessed in ALS patients.
**Study type**	X Interventional ☐ **Non-interventional**<br>☐ Laboratory ☐ Pre-clinical<br>☐ Other/Specifications: ______________________________
**Phase of Development**	Phase II
**Research Setting**	☐ Single site X **Multiple sites**<br>☐ Database-based ☐ Other______________
**Study design**	Phase II randomized, double-blind, placebo-controlled, multicenter (8 MND Centres in Italy: 3 centres in Milan, Novara, Genoa, Turin, Modena, Padua), clinical trial
**Sample size**	63
**Main inclusion**	-Patient diagnosed with a laboratory supported , clinically "probable" or "definite" amyotrophic

**criteria**	lateral sclerosis according to the Revised El Escorial criteria (Brooks, 2000)<br>-Familial or sporadic ALS<br>-Female or male patients aged between 18 and 75 years old<br>-Disease duration from symptoms onset no longer than 18 months at the screening visit<br>-Patient treated with a stable dose of Riluzole (100 mg/day) for at least 30 days prior to screening<br>-Patients with a weight > 50 kg and a BMI ≥18<br>-Patient with a FVC ≥ 70 % predicted normal value for gender, height, and age at the screening visit<br>-Patient able and willing to comply with study procedures as per protocol<br>-Patient able to understand, and capable of providing informed consent at screening visit prior to any protocol-specific procedures<br>-Use of effective contraception both for males and females
**Main exclusion criteria**	-Prior use of Sirolimus<br>-Prior allergy/sensitivity to Sirolimus or macrolides<br>-Any medical disorder that would make immunosuppression contraindicated, including but not limited to, acute infections requiring antibiotics, patients with known diagnosis of HIV, TBC, hepatitis B or C infection or history of malignancy<br>-Severe comorbidities (heart, renal, liver failure), autoimmune diseases or any type of interstitial lung disease<br>-White blood cells<4,000/mm³, platelets count<100,000/mm³, hematocrit<30%<br>-Patient who underwent non invasive ventilation, tracheotomy and /or gastrostomy<br>-Women who are pregnant or breastfeeding<br>-Participation in pharmacological studies within the last 30 days before screening<br>-Patients with known SOD1 mutation or with FALS and a family member carrying SOD1 mutation.
**Study methods incl. treatment/dosing regimen**	Subjects will be enrolled in 3 groups of 21 subjects; treatment will be double blinded to patients and physicians, and will last 18 weeks. Active treatment will include oral Rapamycin at different doses: Rapamycin 1mg/m2/day or Rapamycin 2mg/m2/day. Rapamycin will be administered at fast, in the morning, once a day. Rapamycin levels will be measured at week 1,2,4,8,12,18 (treatment end) to avoid toxicity (>15 ng/ml). Rapamycin dosage will be performed in the morning, before treatment assumption, with High Performance Liquid Chromatography. Treating neurologists will have no access to blood laboratory data. The Local Laboratory Unit, blind to treatment, will send laboratory values together with R values to an independent medical monitor, who will adjust dosages accordingly and who will perform sham adjustments in the placebo group. Caring neurologist will perform dosages adjustments as suggested, without knowing whether they are true or sham adjustments. Patients taking Riluzole will maintain treatment over the entire study duration. Verum and placebo will be made unrecognizable both to patients and physicians. Post-treatment follow up will be 36 weeks. Globally the study will lasts 24 months. To monitor adverse events (AE), examination and routine laboratory work (RL) (cell count, lipids and protein profile, kidney and liver function, C reactive protein) will be performed before taking Rapamycin/placebo, every 2 weeks until week 8, then at week 12, every 6 weeks until week 30 and every 12 weeks until week<br>54. A telephone call to assess safety will be performed every week until week 18.<br>*Non-routine laboratory studies* (NRL) include (baseline and week 8-18-30-54): quantification and characterization of Tregs, lymphocytes phenotype, mTOR downstream pathway activation in PBMC, inflammasome components in PBMC and proinflammatory cytokine production in monocytes, peripheral biomarkers (including albumin, creatinine, CK, vitamin D, plasma neurofilament heavy/light chain protein (NF)). CSF will be taken at baseline and at week 18 to measure NF and to dose Rapamycin (week 18-high-performance liquid chromatography (HPLC) with mass spectrometry (MS) (LC-MS/MS)) to understand whether sufficient levels of Rapamycin can be found in the CNS. A medical monitor will be established with the task of monitoring safety through: safety data downloads and review on monthly basis (adverse events), laboratory data downloads, including Rapamycin levels on fortnightly basis. A Contract Research Organization (CRO) will be in charge for centres monitoring, and an authorized company for pharmacovigilance.
**Primary Endpoint**	Proportion of patients exhibiting a positive response (considered as increase in Treg of at least 30%), comparing baseline and treatment end (WEEK 18) between Rapamycin and placebo arm, using mAbs anti-CD3,-4,-25,-127,-FoxP3 plus activation (HLA-DR,CD38) and homing (CXCR3) markersand flow cytometry (FCM)
**Safety Evaluation**	**Safety assessment**:<br>-<u>Screening visit:</u> informed consent, demographic and clinical data (medical history and ALS history;

Via Giardini, 1355
Baggiovara
41126 Modena
tel. : 059396111
fax.: 0593962409

onset, diagnosis; medication history), inclusion and exclusion criteria, vital signs, weight,height (BMI/BSA), general and neurological examination, check of the patient's currentpractice for effective contraception, FVC, ALSFRSR, MRC, urinalysis and blood sample for haematology and biochemistry, Serum pregnancy test in females of child-bearing potential, Screening for infectious diseases (TB test, HBV, HCV, HIV), ECG (of the last month), Chest X-ray (of the last month).

-Baseline visit: inclusion and exclusion criteria, check of the patient's current practice for effective contraception, menstrual cycle, riluzole and other treatments intake recording; vital signs,weight, height (BMI/BSA), general and neurological examination, FVC, ALSFRSR, MRC, ALSAQ40, urinalysis and blood sample for haematology and biochemistry, non-routine laboratory tests (for biological activity assessment), lumbar puncture, randomisation and treatment allocation

-Every week until week 18*: phone call with adverse events (AE) recording including: infections, symptoms possibly related to respiratory, hepatobiliar and cardiac toxicity, allergic reactions, edema, poor wound healing, increased blood pressure, pain (including stomach and joint pain), nausea, diarrhea, headache, fever, cancers (esp. lymphoma and skin cancer). Treatment and co-treatment recording (if withdrawal, reason recorded)

-Week 2-6: check of the patient's current practice for effective contraception, menstrual cycle, treatment and co-treatment recording (if withdrawal, reason recorded), vital signs, weight, height (BMI/BSA), AE recording, routine laboratory test and Rapamycin dosage. Dose adjustment if necessary.

-Week 8: check of the patient's current practice for effective contraception and menstrual cycle, AE recording, physical examination including vital signs, weight, height (BMI/BSA), neurological examination, MRC, ALSFRS-R and FVC, ALSAQ40, routine blood sample for haematology and blood chemistry, non routine blood sample for biological activity assessment (to be sent to the Laboratory of Immunology of Modena University within 24 hours), Rapamycin dosage, Dose adjustment if necessary. Dispensing of study treatment and study treatment administration.

-Week 4-12: check of the patient's current practice for effective contraception and menstrual cycle, AE recording, physical examination including vital signs, weight, height (BMI/BSA), neurological examination, MRC, ALSFRS-R and FVC, routine blood sample for haematology and blood chemistry, Rapamycin dosage, Dose adjustment if necessary. Dispensing of study treatment and study treatment administration.

-Week 18: check of the patient's current practice for effective contraception and menstrual cycle, AE recording, physical examination including vital signs, weight, height (BMI/BSA), neurological examination, MRC, ALSFRS-R and FVC, ALSAQ40, routine blood sample for haematology and blood chemistry, urinalysis, non routine blood sample for biological activity assessment (to be sent to the Laboratory of Immunology of modena University within 24 hours), Rapamycin dosage. Lumbar puncture with CSF Rapamycin dosage and neurofilaments. Return of study treatment. Tablet accountability. ECG and/or Chest X-ray on medical opinion.

-Week 24: check of the patient's current practice for effective contraception and menstrual cycle, AE recording, co-treatment recording, physical examination including vital signs, weight, height (BMI/BSA) neurological examination, MRC, ALSFRS-R routine laboratory test

-Week 30-42: check of the patient's current practice for effective contraception, menstrual cycles, AE recording, physical examination including vital signs, weight, height (BMI/BSA), neurological examination, MRC, ALSFRS-R and FVC measurement, ALSAQ40, blood sample for haematology and blood chemistry, blood sample will be collected for biological activity assessment (to be sent to the Laboratory of Immunology of Modena University within 24 hours; only at week 30).

-Week 54 (study end): check of the patient's practice for effective contraception, menstrual cycle, AE recording, physical examination including vital signs, weight, height (BMI/BSA), neurological examination, MRC, ALSFRS-R and FVC measurement, ALSAQ40, blood sample for haematology and blood chemistry, blood sample will be collected for biological activity assessment (to be sent to the Laboratory of Immunology of Modena University within 24 hours).

-*For the first ten patients enrolled: clinical and biological assessment for safety reasons will be performed (in addiction to what mentioned before) also at week 1,3,10,14,16 by checking of the patient's current practice for effective contraception, menstrual cycle, treatment and co- treatment recording (if withdrawal, reason recorded), vital signs, weight, height (BMI/BSA), neurological examination, MRC, ALSFRS-R, AE recording, routine laboratory test. All other visits will be done as reported before.

Patients undergoing investigational treatment discontinuation for any reason (except death or

Via Giardini, 1355
Baggiovara
41126 Modena
tel.: 059396111
fax.: 0593962409

	consent retirement) will be followed until the end of the study.<br>**Data recording and study monitoring:** All data will be recorded by an electronic CRF. The study will be monitored by a certified contract research organization (CRO).<br>A medical monitor will be in charge of safety data downloads and review on monthly basis (adverse events), laboratory data downloads, including Rapamycin levels on fortnightly basis. Data safety and monitoring board (DSMB) meetings will be scheduled (starting when the first 10 patients enrolled would have been treated for 3 months and then when 50% of patients would have have completed at least week 8 of treatment and subsequently after every 3 months). All SAE's will be reviewed by the DSMB. As for mild and moderate adverse events, the DSMB will be notified if 50% of all subjects report a given mild or moderate severity adverse effect.<br>**Stopping rules for administering the drug (single patient)**:<br>The following rules will apply regardless of the relationship with treatment:<br>-first occurrence of moderate adverse event: study treatment will be interrupted until adverse event has returned to baseline value or mild intensity, then resumed at the same dose level.<br>-If the same moderate adverse event re-occurs, study treatment will be interrupted until adverse event has returned to baseline or mild intensity, then resumed with a dose reduction, accordingly to the investigator opinion.<br>- In case of severe adverse event, study treatment will be interrupted until adverse event has returned to baseline level or mild intensity, then resumed with a dose reduction, accordingly to the investigator opinion.<br>-In case of life-threatening or disabling adverse event, study treatment will be definitely discontinued<br>**Stopping rules for safety reasons:**<br>Regular DSMB meetings will be scheduled to monitor the excess of AE recording the treating group in comparison with the placebo group. The study will be stopped by the DSMB in case of more than 30% of patients experience one of the following side effects: pneumonia, sepsis, venous thromboembolism, thrombotic thrombocytopenic purpura / hemolytic uremic syndrome, severe leucopenia or anemia or thrombocytopenia, bone necrosis, melanoma, skin cancer, lymphoma. In other words the trial will be stopped if any of the above mentioned SAEs occurs in at least 6 patients per arm.
**Sample size justification**	**Sample size justification**<br>ALS pts have a slight reduction of Treg% (mean±SD:2.1±0.7) with respect to healthy controls (2.6±0.6)(Treg% calculated on total lymphocytes; normal values of total lymphocytes: 1000-4500/mmc; normal values of total Treg: 71.5±17/mmc)(Mantovani,2009).<br>Slowly progressive ALS pts have a number of Tregs that is equal to healthy controls, whereas fast progressors have 31% fewer Tregs than slowly progressing pts, and Treg % is inversely correlated with the rate of disease progression(Beers,2011). These data indicates that ALS pts have 60±17 Treg/mmc (fast progressors: 49.3Treg/mmc; slow progressors: 71.5Treg/mmc). As a result, a "positive response" can be considered an increases of the proportion of Tregs by at least 30%. The null hypothesis is that Rapamycin does not increase significantly the proportion of positive responses in treated pts at 18 weeks compared to their baseline stage and to placebo group. The alternative hypothesis is that Rapamycin determines a proportion of positive responses in at least 50% of treated patients compared to a maximum 5% of patients in the placebo group. The study has been designed to reject the null hypothesis with an alpha error of 0.025 (in order to take into account a multiple comparison with a control arm) and a power of 0.80. For this purpose, a sample of 54 pts randomized in 3 treatment arms would be needed. Considering an average drop out of 15% then a recruitment of 63 patients will be necessary.
**Statistics**	**Statistical methods:**<br>**Separate analyses will be performed in**:<br>1. All randomized subjects receiving at least 1 dose of study medication (**Intention-to-treat population**);<br>2. All randomized subjects excluding protocol deviations (**Per protocol, PP population**).<br>Descriptive statistics will be performed comparing the 2 groups of Rapamycin treatment and placebo. Continuous variables will be described using mean and standard deviation or median and interquartile range; categorical variables will be described as counts and percentages.<br>**Rapamycin activity analysis:**<br>Immune response to Rapamycin (R)) will be analyzed as the difference in positive response to Rapamycin (mean Tregs increase >30%) between the placebo group and the Rapamycin groups. This will be calculated with the use of Treg data obtained at baseline and at week 18.<br>We will compare the mean values of S6RP phosphorylation, of different T, B, NK cell

	subpopulations, of biomarkers, inflammasome, cytokines, comparing baseline and treatment end (18 weeks) between Rapamycin and placebo arm. Mean differences in plasma concentrations from baseline to week 18 in the 2 treatment arms will be calculated and compared using t-test orWilcoxon-Mann-Whitney test.<br>The mean change over time for the same variables as above will be assessed using repeatedmeasures ANOVA, with treatment as between-subjects factor and time as within-subjects factor. Different models will be used, each with a different biomarker of activity as the dependent variable. Models will be adjusted for any unbalanced distribution of the main prognostic factors (e.g. age) between the two treatment arms.<br>**Safety analysis** will be performed in all subjects receiving at least one dose of the experimental drug. All AEs, SAEs and AEs leading to treatment discontinuation will be recorded according to ICH Guidelines, listed and compared in the treatment arms at any follow-up visit and at the end of the study.<br>Differences in tracheostomy-free survival (Kaplan-Meier method) between the treated groups and placebo group will be compared using the log-rank test. Cox's proportional hazard model would be used to adjust for any possible unbalanced prognostic factors. Statistical significance will be set at 0.05 level for a two-tailed test. Missing data will be handled using the last observation carried forward.
**Study Start date**	1st July 2017
**Study Completion date**	30st June 2019

PROTOCOL SIGNATURE PAGE

PROTOCOL TITLE: RAPAMYCIN (SIROLIMUS) TREATMENT FOR AMYOTROPHICLATERAL SCLEROSIS

PROTOCOL CODE: RAP-ALS

COORDINATING INVESTIGATOR AND SPONSOR SIGNATURE:

______________________________ ___**21/04/2017**________

Dr.ssa Jessica Mandrioli Date

(Azienda Ospedaliera-Universitaria di Modena)

Investigator Agreement: I have read the protocol and agree to

- Implement and conduct this study diligently and in strict compliance with the protocol, good clinical practices and all the applicable laws and regulations including the Declaration ofHelsinki and applicable National Laws.
- Maintain all information in confidence.
- Recognize that any substantial changes in Study Protocol must be approved in writing by the Coordinating Investigator, the Sponsor, the corresponding authorities, and the EthicsCommittee before implementation except when necessary to eliminate immediate hazards to subjects or when the changes involve only logistical or administrative aspects of the study.

I have read this protocol in its entirety and I agree to all parts.

______________________________ ______________________________

Investigator's Signature Date

______________________________ ______________________________

Investigator's Name Investigator's Address

____

TABLE OF CONTENT

1. GLOSSARY OF ABREVIATIONS AND DEFINITION OF TERM 12

2. GENERAL INFORMATION 14

3. BACKGROUND & RATIONALE 15

4. PRELIMINARY DATA 16

5. OBJECTIVES 19

5.1 PRIMARY OBJECTIVE 19

5.2 SECONDARY OBJECTIVEs 19

5.2.1 Rapamycin safety and tolerability in a cohort of ALS patients 19

5.2.2 Biological assessment 19

5.2.3 Clinical assessment 20

5.2.4 Quality of Life assessment 20

6. INVESTIGATIONAL PLAN 20

6.1 STUDY DESIGN 20

6.2 STUDY FLOW CHART 22

6.3 WORK PLAN 27

6.3.1 WP1: management and Project Coordination 28

6.3.2 WP2: RCT conduction 28

6.3.3 WP3: Laboratory Assessment (Drug targets and Biomarkers) 30

6.5.4 WP4: Analysis of data and results dissemination 31

7. STUDY POPULATION 32

7.1 INCLUSION CRITERIA 32

7.2 EXCLUSION CRITERIA 32

7.3 PATIENT IDENTIFICATION 33

8. STUDY TREATMENT 34

8.1. STUDY TREATMENT DEFINITION 34

8.1.1. Investigational Medicinal Product (IMP) 34

8.1.2. Concomitant Treatment 34

8.2. INVESTIGATIONAL MEDICINAL PRODUCT 34

8.2.1. Packaging and Labelling 34
8.2.2. Shipment, Storage Conditions and Accountability 35
8.2.3. Patient Compliance 35
8.2.4. Study Treatment Administration 36
8.3. CONCOMITANT MEDICATIONS 37
8.3.1. Mandatory Concomitant Treatment 38
8.3.2. Prohibited Concomitant Treatment 38
8.3.3. Treatment to be given with High Caution 40
8.4 RANDOMIZATION 40
8.5. BLINDING PROCEDURES 41
8.6. MANAGEMENT OF TOXICITY FOR RAPAMYCIN AND MATCHING PLACEBO 41
8.6.1. Call from site to patient once a week for the first 2 months of treatment 41
8.6.2. Procedures to manage adverse reaction potentially related to study treatment 42
9. EVALUATION CRITERIA 49
9.1. PRIMARY CRITERIA 49
9.2. SECONDARY CRITERIA 49
9.2.1. Safety Variables 49
9.2.2. Biological Activity Assessment 50
9.2.3. Clinical Assessment 51
9.2.4. Quality of Life 51
10. ASSESSMENT SCHEDULE 51
10.1. **SCREENING VISIT** 51
10.2. **BASELINE VISIT** 52
10.3.1 VISIT WEEK 1 (for the first 10 patients enrolled) 53
10.3.2 VISIT WEEK 1 (for the following 53 patients enrolled) 54
[illegible] VISITS WEEK 3, WEEK 10, WEEK 14, WEEK 16, TREATMENT PERIOD (for the first 10 patients enrolled) 54
[illegible] VISITS WEEK 5, WEEK 7, WEEK 9, WEEK 11, WEEK 13, WEEK 15, WEEK 17, TREATMENT PERIOD (for the first 10 patients enrolled) 54
[illegible] VISITS WEEK 3, WEEK 5, WEEK 7, WEEK 9, WEEK 11, WEEK 13, WEEK 15, WEEK 17, TREATMENT PERIOD (for the following 53 patients enrolled) 55

10.5. VISITS W2, W6 TREATMENT PERIOD 55
10.6. VISITS W4, W12 TREATMENT PERIOD 55
10.7. VISITS W8 TREATMENT PERIOD 56
10.8. END OF TREATMENT VISIT , WEEK 18 56
10.9. FOLLOW UP VISITS, WEEK 24 57
10.10. FOLLOW UP VISITS, WEEK 30, WEEK 42 57
10.11 END OF STUDY VISIT (WEEK 54) 58
10.12. WITHDRAWAL OF PATIENTS 58
11. STATISTICAL METHODS 60
11.1. SAMPLE SIZE JUSTIFICATION 60
11.2 STATISTICAL ANALYSIS 60
11.3 STOPPING RULES FOR SAFETY REASONS 61
12. DATA RECORDING AND STUDY MONITORING 62
13. DATA MANAGEMENT 62
14.ETHICS 62
14.1 ETHICAL CONSIDERATIONS 62
14.2 SUBJECT INFORMATION AND INFORMED CONSENT 62
14.3 PATIENT WITHDRAWALS AND DROPOUTS 63
14.4 ETHIC COMMITTEE APPROVAL 63
14.5 INDEPENDENT DATA MONITORING COMMITTEE 64
15. ADMINISTRATIVE CONSIDERATIONS 64
16. REFERENCES 67
17. APPENDIXES 69
17.1. ADVERSE EVENTS 69
17.1.1. Definition of Adverse Event 69
17.1.2. Definition of Serious Adverse Event 69
17.1.3. Reporting of Adverse Events 70
17.1.4. Reporting of Serious Adverse Events 70

17.1.5. Causality Assessment 71
17.1.6. Intensity of Adverse Events 71
17.1.7. Actions Taken in Response to an Adverse Event 71
17.1.8. Follow-Up on Ongoing Adverse Events 72
17.2 DECLARATION OF HELSINKI – ETHICAL PRINCIPLES FOR MEDICAL RESEARCH INVOLVING HUMAN SUBJECTS 72
17.3 ITALIAN VERSION OF THE ALSFRS-R 77
17.4 ITALIAN VERSION OF ALSAQ-40 80
17.5 ITALIAN VERSION OF MRC 83

1. GLOSSARY OF ABREVIATIONS AND DEFINITION OF TERM

AE	Adverse Event
ALS	Amyotrophic Lateral Sclerosis
ALSFRS-R	Amyotrophic Lateral Sclerosis Functional Rating Scores – Revised
ALSAQ-40	Amyotrophic Lateral Sclerosis Assessment Questionnaire
ALT	Alanine Aminotransferase
ANC	Absolute Neutrophil count
APTT	Activated Partial Thromboplastin Time
AST	Aspartate Aminotransferase
BBB	Blood Brain Barrier
BMI	Body Mass Index
BUN	Blood Urea Nitrogen
CI	Confidence Interval
CR	Complete Response
CRA	Clinical Research Assistant
CRF	Case Report Form
CSF	Cerebrospinal Fluid
CT Scan	Computed Tomography scan
CTACAE	Common Terminology Criteria for Coding Adverse Events
CYP	Cytochrome
C9ORF72	Chromosome 9 open reading frame 72
ΔFS	Disease progression rate
EC	Ethics Committee
ECG	Electrocardiogram
Ecrf	Electronic CRF
EDC	Electronic Data Capture
Eef2K	eukaryotic Elongation Factor 2 Kinase
EMEA	European Medicines Agency
FDA	Food and Drug Administration
FOXO1	Forkhead box O1
FOXO3	Forkhead box O3
FoxP3	Forkhead box P3
FTD	Fronto-Temporal Dementia
FTLD	Fronto-Temporal Lobar Degeneration
FUS	Fused in sarcoma
FVC	Forced Vital Capacity
Gamma GT	Gamma-Glutamyl Transferase
GCP	Good Clinical Practice
G-CSF	Granulocyte Colony-Stimulating Factor
GMP	Good Manufacturing Practice
GM-CSF	Granulocyte-Macrophage Colony-Stimulating Factor
HIV	Human Immunodeficiency Virus
HIF1a	Hypoxia Inducible Factor 1a
INR	International Normalised Ratio
IMP	Investigational Medicinal Product
ITT	Intent To Treat
IV	Intravenous
LDH	Lactate Dehydrogenase
LLN	Lower Limit of Normal
LO	Last Observation

Via Giardini, 1355
Baggiovara
41126 Modena
tel. : 059396111
fax.: 0593962409

MRC	Medical Research Council
mTOR	Mechanistic Target of Rapamycin
mTORC1	Mtor Complex 1
mTORC 2	Mtor Complex 2
PLT	Platelet
PP	Per Protocol
PR	Partial Response
PT	Prothrombin Time
PTT	Partial Thromboplastin Time
OPTN	Optineurin
QOL	Quality of Life
R-EEC	Revised El Escorial Criteria
RAPTOR	regulatory associated protein of mTOR
RICTOR	Rapamycin-insensitive companion of mTOR
SAE	Serious Adverse Event
SOD1	Superoxide dismutase 1
SOP	Standard Operating Procedure
SPC	Summary of Product Characteristics
SQSTM1	Sequestosome 1
SREBP	Sterol-Response Binding Protein
S6K1/2	S6 protein kinases 1 and 2
S6RP	S6 ribosomal protein
TBK1	TANK-Binding Kinase 1
TDP-43	TAR DNA-binding protein, 43-KD
TGF-β	Transforming Growth Factor β
T-reg	Regulatory T Cells
UBI	Ubiquinate Inclusions
UBQLN2	Ubiquilin 2
ULK1	Unc-51-like kinase 1 complex
ULN	Upper Limit of Normal
VAPB	Vesicle-associated membrane protein-associated protein B
VC	Vital capacity
VCP	Valosin containing protein
4E-BPs	Eukaryotic initiation factor 4E-binding proteins

2. GENERAL INFORMATION

**PROJECT TITLE**	**RAPAMYCIN (SIROLIMUS) TREATMENT FOR AMYOTROPHIC LATERAL SCLEROSIS**
**ACRONYM**	RAP-ALS
**PRINCIPAL INVESTIGATOR**	JESSICA MANDRIOLI
**HOST INSTITUTION**	DEPARTMENT OF NEUROSCIENCE, AZIENDA OSPEDALIERO UNIVERSITARIA – MODENA
**CONSORTIUM PARTNERS**	1) PROF. ANDREA COSSARIZZA, UNIVERSITY OF MODENA AND REGGIO EMILIA, MODENA<br>2) PROF. ROBERTO D'AMICO, UNIVERSITY OF MODENA AND REGGIO EMILIA, MODENA<br>3) DR. LETIZIA MAZZINI, A.O.U. MAGGIORE DELLA CARITA', NOVARA<br>4) DR. CLAUDIA CAPONNETTO, IRCCS A.O.U. S.MARTINO IST, GENOVA<br>5) PROF. ADRIANO CHIÒ, UNIVERSITY OF TORINO, TORINO<br>6) DR. ELEONORA DALLA BELLA, IRCCS ISTITUTO CARLO BESTA, MILANO<br>7) DR. CHRISTIAN LUNETTA, CENTRO CLINICO NEMO, FONDAZIONE SERENA ONLUS, MILANO<br>8) DR. KALLIOPI MARINOU, IRCCS FONDAZIONE SALVATORE MAUGERI, MILANO<br>9) DR. GIANNI SORARU', UNIVERSITY OF PADOVA, PADOVA
**PROJECT DURATION**	24 months

Via Giardini, 1355
Baggiovara
41126 Modena
tel.: 059396111
fax.: 0593962409

3. BACKGROUND & RATIONALE

Protein aggregates into neurons and glial cells are common features of ALS pathology both in familial and sporadic forms, and have a key role in ALS initiation and progression. Such aggregatesinclude proteins encoded by genes that cause ALS when mutated (SOD1,TDP43,FUS encoded by *SOD1*,*TARDBP*, and *FUS* respectively). Several genes (*C9ORF72*, *VCP*, *UBQLN2*, *OPTN*, *NIPA1*, *SQSTM1*, *TBK1)* acting on RNA processing and protein degradation pathways are involved in TDP43 proteinopathy (Cirulli, 2015), which is also a hallmark of >95% of sporadic/non mutated ALS and links a spectrum of neurodegenerative diseases, ranging from ALS to FTD (Thomas, 2013). Protein degradation machinery and autophagy have a crucial role in dealing with misfolded aggregated proteins. Genetic disruption of autophagy in the brain results in widespread inclusion bodies with ubiquitinated protein and early neuronal death (Komatsu, 2006). **In ALS at least five genes (*UBQLN2,SQSTM1,OPTN,VCP,TBK1*) have strong links with protein degradation pathways** as their products contribute to recruitment of ubiquitinated proteins to the autophagosome. Autophagy is also required for the removal of aberrant stress granules involved in ALS pathology (Buchan, 2013) and for downregulation of inflammasome activity, which is activated in response to cellular inclusions formation (Shi, 2012).***TBK1, OPTN, and SQSTM1* converge on autophagy and neuroinflammation**, suggesting that compounds which affect both pathways may be promising.

Mechanistic Target of Rapamycin (Mtor) integrates signals to elicit critical outputs includinggrowth control, protein synthesis, gene expression, and metabolic balance. Mtor importance to brainfunction is underlined by several disorders showing Mtor dysfunctions (Lipton, 2014). **The action of Rapamycin is based on Mtor Complex 1 (Mtorc1) inhibition** (Figure 1). Mtorc1 targets regulatory proteins in cell signaling and **regulates autophagy** by inhibiting the unc-51-like kinase 1complex. Inhibition of Mtorc1 by Rapamycin stimulates autophagy, through the formation ofautophagosome from the phagophore.

Mtor inhibitors maintain homeostasis of T cells by preventing them from engaging alternative paths. Indeed, naïve CD4+ Tcells can develop into TH1,TH2 or TH17 effectors using pathways promoted by Mtor. Conversely, Mtor inhibits the induction of Tregs, cells that downregulateimmune activation. **Inhibition of Mtorc1 by Rapamycin expands Tregs** and, in Msod1 mice increased Tregs and **induction of M2 microglia (with anti-inflammatory properties)** were associated with stable phase of disease. In ALS patients, blood percentage of Tregs inversely correlated with progression rate, and FoxP3 levels were early predictors of ALS progression and survival (Beers, 2011). Thus Tregs may be considered important therapeutic targets in ALS.

Based on these premises, we are going to perform the first in human clinical trial in ALS patients with Rapamycin, a drug that enhances autophagy, facilitates TDP43 clearance (Barmada, 2014) **and regulates immune responses.**

4. PRELIMINARY DATA

Rapamycin has been tested in several neurodegeneration models (including Huntington and Parkinson diseases), because it is an Mtor-dependent autophagy activator, and accelerates the removal of abnormal accumulation of aggregated proteins (Ravikumar, 2004) with beneficial effects (Lipton, 2014).

In two cell lines, inhibition of Mtor by Rapamycin reduced TDP43 fragments accumulation and restored TDP-43 nuclear localization (Caccamo, 2009). In murine and in human stem cell-derived neurons and astrocytes with mutant TDP43, autophagy enhancement improved TDP43 clearance and localization and enhanced survival, showing that autophagy induction mitigates neurodegeneration by TDP43 clearance (Barmada, 2014).

Early Rapamycin administration to a mouse model with FTLD and cytoplasmic TDP43 ubiquinate inclusions (UBIs) rescued learning/memory deficiencies and motor function disorders. This was associated to reduction of neuronal loss and of TDP43 UBIs (Wang, 2012).

Treatment of zebrafish embryos with Rapamycin yielded an amelioration of locomotor phenotype in a SQSTM1 knock-down model (Lattante, 2014).

Treatment with Rapamycin of larvae of a Drosophila model carrying VAPB (P58S) mutation determined reversal of VAP(P58S) bouton phenotypes (Deivasigamani, 2014).

However, the failure of Rapamycin with aggravation of neuronal death has also been reported in Msod1 mice. In this study Msod1 mice (which reflects the pathogenic mechanisms of SOD1 ALS, whereas the majority of ALS presents TDP43 UBIs) were treated with 2 mg/kg body weight/day, a dosage>50 times higher than that used in clinical practice (usually 2 mg/day), which gives blood concentrations that have well known toxic effects. A further study showed that this effect was due to excessive immunosuppression, as the treatment on ALS mice lacking mature lymphocytes increased ALS survival (Staats, 2013). However, being the evidences on Rapamycin action mainly on models linked to TDP43 pathology, we will exclude patients carrying SOD1 mutations.

As Mtor has a role in organisms longevity (Lipton 2014), Rapamycin has been tested to this extent and it resulted to extend the lifespan of various mouse strains (Harrison, 2009). Rapamycin, then, has been shown to protect against several types of neuronal injury (stroke, atherosclerosis, traumaticbrain and spine injury). Recently it resulted effective in improving respiratory function in lymphangioleiomyomatosis (McCormack,2011).

Rapamycin has a well known immunosuppressive action: it is used for the cure of transplanted patients, and in clinical trials to test efficacy in autoimmune disorders, CNS tumors, and other neurological disorders (such as tuberous sclerosis).

As for the role of immunity in ALS, recent studies suggest that innate and adaptive immune systems contribute to ALS progression: Msod1 Tregs co-cultured with activated Msod1 microglia attenuated the expression of microglial toxic factors by IL4 release, and promoted MN survival by suppressing M1 activation, inducing an M2 protective phenotype, and reducing the release of ROS. Tregs passive transfer into ALS mice prolonged survival, and FoxP3 Mrna in Msod1 mice spinal cord decreased with disease progression (Beers,2011). In patients, blood Tregs % inversely correlated with ALS progression rate, and FoxP3 levels were early predictors of ALS progression and survival. These data were confirmed in post-mortem studies (Beers,2011). Inhibiting the Mtor pathway, Rapamycin induces de novo FoxP3 expression and expands Treg.

The entity of the increase of Tregs induced by Rapamycin is completely unknown in ALS patients. For this reason we are planning this first trial focused on showing immunological targets

engagement by Rapamycin in ALS patients. Moreover, given its large natural structure, there was initial concern that Rapamycin might not be able to cross the blood brain barrier (BBB); however, in one study sufficient levels of Rapamycin could be found in brain tumors when administered at therapeutic doses (Cloughesy,2008). To further address this concern, we are planning to test Rapamycin titer in CSF at treatment end (using high-performance liquid chromatography (HPLC) with mass spectrometry (MS) (LC-MS/MS)). This procedure will also allow to understand if Rapamycin action depends on BBB crossing or on its action on T-reg cells.

Figure 1: Mtorc1 patway

From the top: regulation of Mtor signaling by a variety of intrinsic and extrinsic factors. Downstream of Mtor, several cellular processes are regulated. Mtor exists in 2 complexes: Mtor complex 1 (Mtorc1), containing the scaffolding protein RAPTOR is sensitive to Rapamycin, whereas Mtor complex 2 (Mtorc2) contains RICTOR. Different actions of Mtorc1:

I) Protein Synthesis: substrates of Mtorc1 include the p70 ribosomal S6 protein kinases 1 and 2 (S6K1/2) and the eukaryotic initiation factor 4E-binding proteins (4E-BPs). S6K1/2 both phosphorylate ribosomal protein S6. S6K1 also phosphorylates Eif4B and eukaryotic elongation factor 2 kinase (Eef2K),further stimulating translation initiation and elongation of nascent peptide chains.

II) Autophagy: Mtorc1 signaling regulates autophagy through inhibition of the unc-51-like kinase 1(ULK1) complex. When Mtorc1 kinase activity is inhibited, autophagosome can form from the phagophore. To allow autophagosome formation, Vps34 forms a complex with Beclin 1and ATG14L. Beclin 1 interacts with factors (Ambra, Bif1, and Bcl-2) that modulate its binding to Vps34. Then autophagosome formation requires Atg12 and LC3 (ubiquitin-like protein conjugation systems) that are essential for the formation of the phagophore; LC3 system is involved in autophagosome transport and maturation. Autophagosomes which reach maturation fuse with lysosomes to degrade their cargo and recycle essential biomolecules; this process is regulated by VCP and UBQLN2, which functions in both the ubiquitin-proteasome and autophagy pathways.

III) Immune response: In steady state, negative inhibitory molecules for Mtor actively maintain the homeostasis of T cells by preventing them from engaging alternative paths. Following antigen stimulation, naïve CD4+T cells develop into TH1, TH2 and TH17 effector cells; this pathway is promoted by Mtor, which instead inhibits the induction of Treg. Inhibition of Mtor induces de novo forkhead box P3 (FOXP3) expression. Two downstream pathways mediate the inhibitory effects of Mtor on induced Treg cell differentiation: SMAD3, a key transcription factor downstream of transforming growth factor β (TGF-β) iluzole for FOXP3 induction, is antagonized by Mtor iluzole in multiple cell types including T cells; forkhead box O1 (FOXO1) and FOXO3, which induce FOXP3 expression, are inactivated by AKT-dependent phosphorylation, although how this is controlled by Mtor complex 2 (Mtorc2) iluzole requires further studies

IV) Mitochondrial biogenesis: Mtorc1 stimulates the association of the YY1 transcription factor with the transcriptional coactivator PGC1a, with activation of a mitochondrial gene program.

V) Lipogenesis: Mtorc1 regulates the sterol-response binding proteins (SREBPs), which regulate lipogenesis.

VI) Hypoxia: Mtorc1 regulates the cellular response to hypoxia through regulation of the transcription and translation of the hypoxia inducible factor 1a (HIF1a).

Via Giardini, 1355
Baggiovara
41126 Modena
tel.: 059396111
fax.: 0593962409

Figure 1: Mtorc1 patway

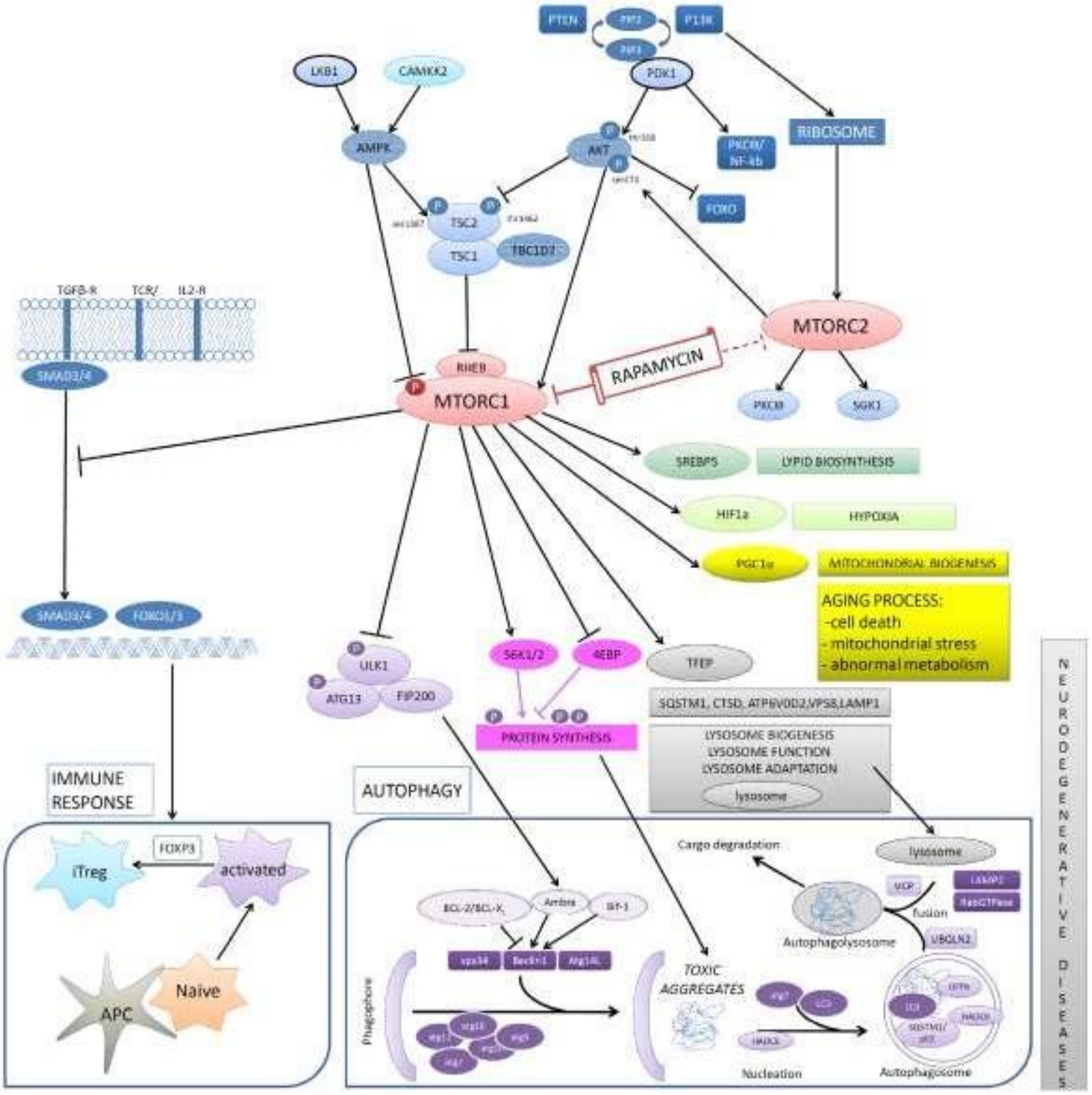

5. OBJECTIVES

5.1 PRIMARY OBJECTIVE

The **primary objective** is to assess whether different Rapamycin doses increase Tregs number in ALS patients compared to the control arm.

5.2 SECONDARY OBJECTIVES

5.2.1 Rapamycin safety and tolerability in a cohort of ALS patients

Occurrence of Adverse Events (AE), changes on clinical examination including vital signs and weight, and laboratory exams (biochemistry, hematology and urinalysis)
Safety and tolerability will be assessed by periodic monitoring of possible adverse events, including increased risk of infections, of allergic reactions, edema, poor wound healing, alteration of blood cells and platelets, increased serum levels of cholesterol andtriglycerides, increased urine protein levels, increased risk of cancers (especially lymphoma and skin cancer), respiratory, liver, hepatobiliar and cardiac taxicities. Common symptoms due to Rapamycin will be registered, including increased blood pressure, pain (including stomach and joint pain), nausea, Riluzole, headache, fever, urinary tract infection, anemia, low platelet count. Death from any cause or tracheotomy will be also considered.

5.2.2 Biological assessment

-To assess Rapamycin capacity to pass through blood brain barrier (BBB). Using different Rapamycin doses, and measuring Rapamycin levels in CSF at week 18, we will evaluate the minimum dose to have Rapamycin in CSF to address concerns on Rapamycin capability of passing BBB. The dosage will be performed in the laboratory that will measure Rapamycin valuedsin the blood, using high-performance liquid chromatography (HPLC) with mass spectrometry (MS) (LC-MS/MS).
-To analyze Rapamycin efficacy in inhibiting Mtor pathway, by quantifying the phosphorylation of the S6 ribosomal protein (S6RP) comparing baseline and week 8, 18 (treatment end), 30 and 54 between Rapamycin arms and placebo arm.
-To identify changes in activation and homing capabilities of different T, B, NK cell subpopulations comparing baseline and week 8, 18 (treatment end), 30 and 54 between Rapamycin arms and placebo arm.
-To study Rapamycin effects on biomarkers: we will measure changes in different biomarkers (including peripheral and CSF biomarkers i.e. creatinine and albumin, CK, vitamin D, plasma/CSF neurofilament heavy/light chain protein) comparing baseline and week 8, 18 (treatment end), 30 and 54 between Rapamycin arms and placebo arm
-To identify Rapamycin-induced changes in inflammatory status, by the molecular analysis of the inflammasome system baseline and week 8, 18 (treatment end), 30 and 54 between Rapamycin arms and placebo arm.

Via Giardini, 1355
Baggiovara
41126 Modena
tel.: 059396111
fax.: 0593962409

5.2.3 Clinical assessment

-Amyotrophic Lateral Sclerosis functional rating scale (ALSFRS)-Revised from baseline to week 4, 8, 12, 18, 30, 42 and week 54

-Overall survival from randomization to date of documented death or tracheostomy

-Survival rate at week 18, 30, 42 and week 54

-Forced vital capacity (FVC) score from baseline to week 4, 8, 12, 18, 30, 42, 54

5.2.4 Quality of Life assessment

-ALSAQ-40 from baseline to week 8, 18, 30 and week 54

6. INVESTIGATIONAL PLAN

6.1 STUDY DESIGN

We are going to perform the first study with Rapamycin in ALS patients, carrying the double potential effect of enhancing TDP43 autophagy and of targeting the immune system through Treg expansion. We are going to plan a phase II randomized, double-blind, placebo-controlled, multicenter, clinical trial (CT)

Patients affected by probable (clinically or laboratory supported) or definite ALS (Brooks, 2000) will undergo screening procedures that must be completed during the 14 day screening period. All patients must adhere to inclusion and exclusion criteria through clinical evaluation and laboratory and instrumental assessment. Screening assessments include blood sampling,biochemical and pregnancy evaluations (for fertile females) which will be performed in the site's local laboratory, General and neurological examinations, MRC, ALSFRS-R, Chest Radiography and ECG, spiroemtry. Laboratory evaluations will be repeated on the day of randomization (treatment day 1) and sent to the central laboratory.

Patients will be randomized to receive either Rapamycin 1 mg/m2/day or 2 mg/m2/day or placebo; randomization will be balanced, 1:1:1. Given the heterogeneity in ALS progression, patients will be stratified by ΔFS (progression rate). Progression rate will be calculated according to Kimura and colleagues as: ΔFS=48-ALSFRSR score at a given time/duration of disease from onset to that time (months) (18). Progression rate will be calculated at randomization.

Rapamycin or placebo will be administered together with Riluzole. Riluzole, an antiglutamatergic agent that inhibits the presynaptic release of glutamate, is the only drug for the treatment of ALS approved by the US Food and Drug Administration (FDA) and by the European Medicinal Agency (EMA). Riluzole is the only compound that demonstrated a beneficial effect on ALS patients, although with only modest increase in survival.

None of the several drugs resulted to be effective in animals models of ALS showed effective results in the clinical trials on people with ALS; these negative results may be explained by

Via Giardini, 1355
Baggiovara
41126 Modena
tel.: 059396111
fax.: 0593962409

methodological issues, and by problems in the use of animal-models largely based on the Msod1 mouse whose validity has been widely questioned. In particular it is questioned whether the SOD1 mouse is simply a model of motoneuron degeneration or specifically SOD1-ALS rather thansporadic ALS. In fact, research on this mutation showed that only 5-10% of the totality of ALS cases is caused by SOD1 mutation, whereas the vast majority of familial and sporadic ALS show TDP-43 inclusions.

Study treatment will be continued for 18 weeks. After treatment end patients will be followed up for further 36 weeks. This large period of follow up has the aim to assess potential late side effects of the treatment and to assess whether eventual biological or clinical effects of the treatment with respect to placebo, may last after drug discontinuation.
For the first 10 patients enrolled, clinical and biological safety assessments will be performed every week for the first month, and then every 2 weeks until the end of treatment. When the first 10 patients will reach 3 months of treatment and independent Data safety and Monitoring Board (DSMB) will evaluate clinical course in relation to safety; a report will be sent to the Ethic Committee of the coordinating centre before proceeding with the enrollment of the remaining 53 patients. For these remaining 53 patients clinical assessments will be performed every 2 weeks for the first month, and then every 4 weeks during treatment duration.
If at either the interim or the final analyses safety concerns will arise, the trial will be stopped and all available data will be reviewed by the Study Steering Committee.

Figure 2: Study design

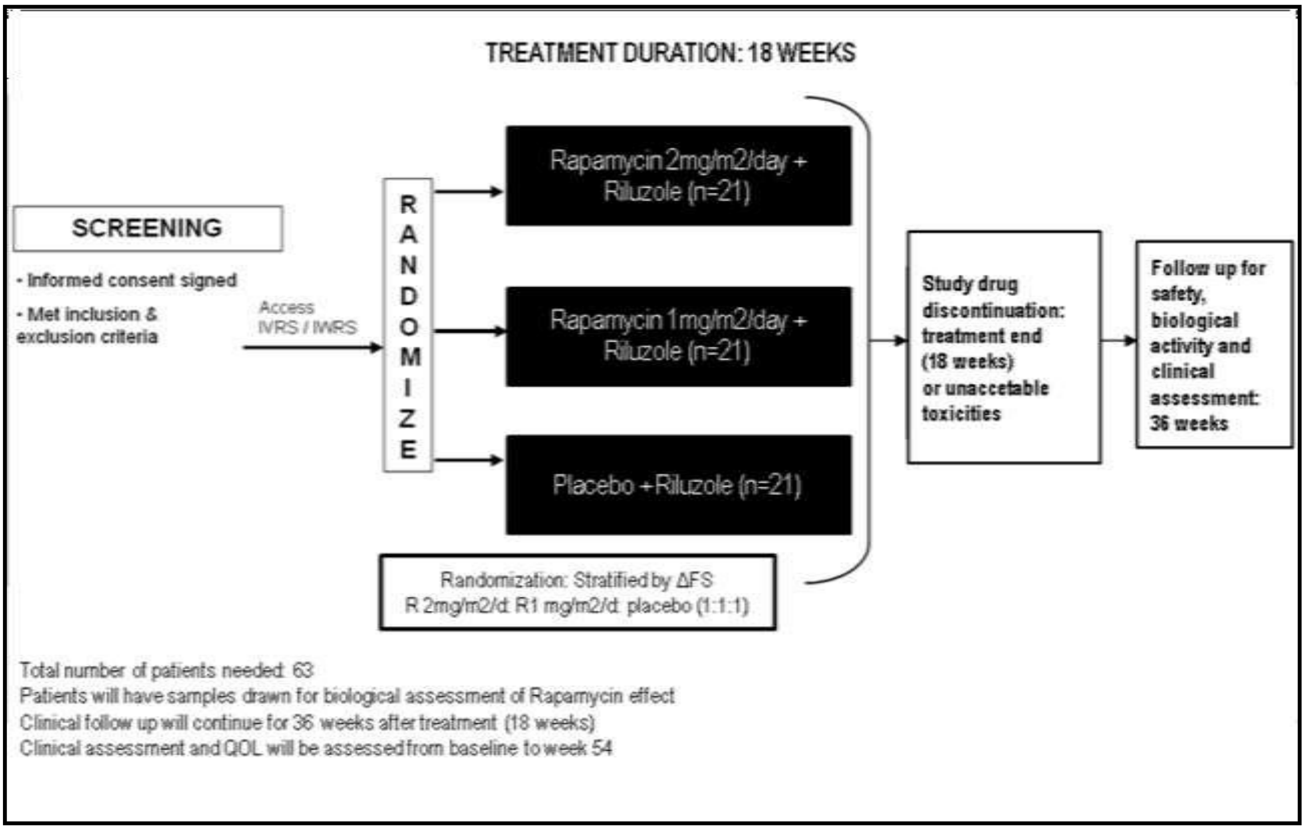

6.2 STUDY FLOW CHART

Each patient will be treated for 18 weeks, with a follow up period of 36 weeks. Patients must be followed at the study centre according to the flow charts.
Two different flow charts are presented: **tables 1a and 2a are exclusively related to the first ten patients enrolled in the study.**
Tables 1b and 2b have to be followed for the following 53 patients.

Table 1a: Study flow chart for the first 10 patients who will be enrolled

	Pre-treatment		Treatment				Treatment end	Follow up		Study end
**Examinations**	**Screening (VS)**	**Baseline (W0)**	**W1,W3, W10, W14, W16**	**W5,W7, W9, W11, W13, W15, W17**	**W2, W6**	**W4, W8, W12**	**W18**	**W24**	**W30, W42**	**W54**
**Time window**		**<2 weeks from screening**	**± 1 day**	**± 1 day**	**± 1 day**	**± 1 day**	**± 2 days**	**± 3 days**	**± 3 days**	**± 7 days**
Informed Consent	x									
Medical History	x									
Inclusion exclusion criteria	x									
Patient able to understand and follow the patient card procedures	x									
Phone call				x						
**Biological activity**										
T-regs		x				x(1)	x		x(2)	x
Lymphocytes phenotype		x				x(1)	x		x(2)	x
S6RP phosphorilation		x				x(1)	x		x(2)	x
Inflammasome		x				x(1)	x		x(2)	x
Peripheral biomarkers		x				x(1)	x		x(2)	x
CSF		x					x			
**Safety assessment**										
Adverse events		x	x	x	x	x	x	x	x	x
Vital signs	x	x	x		x	x	x	x	x	x
Physical examination	x	x	x		x	x	x	x	x	x
Chest X-ray	x						x(3)			
ECG	x						x(3)			
Hematology	x	x	x		x	x	x		x	x
Biochemistry	x	x	x		x	x	x		x	x
Urinalysis	x	x					x			x
Pregnancy test	x						x			
Infectious markers	x									
Rapamycin blood dosage			**Only at week 1**		x	x	x			
**Clinical assessment**										

Neurological examination	x	x	x			x	x	x	x	x
ALSFRS-R	x	x	x			x	x	x	x	x
MRC	x	x	x			x	x	x	x	x
FVC	x	x				x	x		x	x
BMI/BSA	x	x	x			x	x	x	x	x
**Quality of life assessment**										
ALSQ40		x				x	x		x	x
**Study treatment dispensation and compliance**										
Study treatment dispensation		x				x				
Study treatment compliance			x	x	x	x	x			
Concomitant medications	x	x	x	x	x	x	x	x	x	x

(1)Only at week 8
(2)Only at week 30
(3)Only if necessary on medical opinion

Table 2A: Blood test: time and location for the first 10 patients who will be enrolled

	**Pre-treatment**		**Treatment**				**Treatment end**	**Follow up**		**Study end**
**Examinations**	**Screening (VS)**	**Baseline (W0)**	**W1,W3, W10, W14, W16**	**W5, W7,W9, W11,W13, W15,W17**	**W2, W6**	**W4, W8, W12**	**W18**	**W24**	**W30, W42**	**W54**
**Time window**		**<2 weeks from screening**	**± 1 day**	**± 1 day**	**± 1 day**	**± 1 day**	**± 2 days**	**± 3 days**	**± 3 days**	**± 7 days**
**Routine laboratory tests**										
Cell count with formula(1)	**L(2)**	**L**	**L**		**L**	**L**	**L**	**L**	**L**	**L**
Creatinine	**L**	**L**	**L**		**L**	**L**	**L**	**L**	**L**	**L**
Albumin	**L**	**L**	**L**		**L**	**L**	**L**	**L**	**L**	**L**
Protein electrophoresis	**L**	**L**	**L**			**L**	**L**		**L**	**L**
AST	**L**	**L**	**L**		**L**	**L**	**L**	**L**	**L**	**L**
ALT	**L**	**L**	**L**		**L**	**L**	**L**	**L**	**L**	**L**
GammaGT	**L**	**L**	**L**		**L**	**L**	**L**	**L**	**L**	**L**
Glucose	**L**	**L**	**L**		**L**	**L**	**L**	**L**	**L**	**L**
PCR	**L**	**L**	**L**		**L**	**L**	**L**	**L**	**L**	**L**
Sodium	**L**	**L**	**L**		**L**	**L**	**L**	**L**	**L**	**L**
Potassium	**L**	**L**	**L**		**L**	**L**	**L**	**L**	**L**	**L**
Urinalysis	**L**	**L**	**L**		**L**	**L**	**L**	**L**	**L**	**L**
VES	**L**		**L**							
CK		**L**				**L**	**L**		**L**	**L**
Total cholesterol		**L**				**L**	**L**		**L**	**L**
LDL Cholesterol		**L**				**L**	**L**		**L**	**L**
Triglycerides		**L**				**L**	**L**		**L**	**L**
Uric acid		**L**				**L**	**L**		**L**	**L**
Urea		**L**				**L**	**L**		**L**	**L**
Ferritin		**L**				**L**	**L**		**L**	**L**

Pregnancy test	**L**									
HIV, Hepatitis B, Hepatitis C, TB	**L**									
**Biological activity tests**										
T-regs		**MO(3)**				**MO (4)**	**MO**		**MO(5)**	**MO**
Lymphocytes phenotype		**MO**				**MO (4)**	**MO**		**MO(5)**	**MO**
S6RP phosphorilation		**MO**				**MO (4)**	**MO**		**MO(5)**	**MO**
Inflammasome		**MO**				**MO (4)**	**MO**		**MO(5)**	**MO**
Rapamycin blood dosage				**Only at W1, L**	**L**	**L**	**L**			
**Peripheral biomarkers**										
Vitamin D		**L**				**L**	**L**		**L**	**L**
Folates		**L**				**L**	**L**		**L**	**L**
Neurophilaments		**MO**				**MO**	**MO**		**MO**	**MO**
**CSF tests**										
Rapamicyn dosage							**L**			
Neurophilaments		**MO**					**MO**			

(1) Erythrocytes, Hematocrit, Hemoglobin, Platelets, Leucocytes, Neutrophils, Eosinophils, Basophils, Lymphocytes, Monocytes,
(2) L= local laboratory of the centre
(3) MO= Laboratory of Immunology (Prof. Cossarizza) Of the University of Modena and Reggio Emilia
(4)Only at week 8
(5)Only at week 30

Table 1b: Study flow chart for the following 53 patients who will be enrolled

	**Pre-treatment**		**Treatment**			**Treatment end**	**Follow up**		**Study end**
**Examinations**	**Screening (VS)**	**Baseline (W0)**	**W1,W3, W5, W7,W9, W11,W13, W15,W17**	**W2, W6**	**W4, W8, W12**	**W18**	**W24**	**W30, W42**	**W54**
**Time window**		**<2 weeks from screening**	**± 1 day**	**± 1 day**	**± 1 day**	**± 2 days**	**± 3 days**	**± 3 days**	**± 7 days**
Informed Consent	**x**								
Medical History	**x**								
Inclusion exclusion criteria	**x**								
Patient able to understand and follow the patient card procedures	**x**								
Phone call			**x**						
**Biological activity**									
T-regs		**x**			**x(1)**	**x**		**x(2)**	**x**

Lymphocytes phenotype		x			x(1)	x		x(2)	x
S6RP phosphorilation		x			x(1)	x		x(2)	x
Inflammasome		x			x(1)	x		x(2)	x
Peripheral biomarkers		x			x(1)	x		x(2)	x
CSF		x				x			
**Safety assessment**									
Adverse events		x	x	x	x	x	x	x	x
Vital signs	x	x		x	x	x	x	x	x
Physical examination	x	x		x	x	x	x	x	x
Chest X-ray	x					x(3)			
ECG	x					x(3)			
Hematology	x	x		x	x	x		x	x
Biochemistry	x	x		x	x	x		x	x
Urinalysis	x	x				x			x
Pregnancy test	x					x			
Infectious markers	x								
Rapamycin blood dosage			**Only at week 1**	x	x	x			
**Clinical assessment**									
Neurological examination	x	x			x	x	x	x	x
ALSFRS-R	x	x			x	x	x	x	x
MRC	x	x			x	x	x	x	x
FVC	x	x			x	x		x	x
BMI/BSA	x	x			x	x	x	x	x
**Quality of life assessment**									
ALSQ40		x			x	x		x	x
**Study treatment dispensation and compliance**									
Study treatment dispensation		x			x				
Study treatment compliance			x	x	x	x			
Concomitant medications	x	x	x	x	x	x	x	x	x

(4)Only at week 8
(5)Only at week 30
(6)Only if necessary on medical opinion

Table 2b: Blood test: time and location for the following 53 patients who will be enrolled

	Pre-treatment		Treatment			Treatment end	Follow up		Study end
**Examinations**	**Screening (VS)**	**Baseline (W0)**	**W1,W3, W5, W7,W9, W11,W13, W15,W17**	**W2, W6**	**W4, W8, W12**	**W18**	**W24**	**W30, W42**	**W54**
**Time window**		<2 weeks from screening	± 1 day	± 1 day	± 1 day	± 2 days	± 3 days	± 3 days	± 7 days

**Routine laboratory tests**									
Cell count with formula(1)	**L(2)**	**L**		**L**	**L**	**L**	**L**	**L**	**L**
Creatinine	**L**	**L**		**L**	**L**	**L**	**L**	**L**	**L**
Albumin	**L**	**L**		**L**	**L**	**L**	**L**	**L**	**L**
Protein electrophoresis	**L**	**L**			**L**	**L**		**L**	**L**
AST	**L**	**L**		**L**	**L**	**L**	**L**	**L**	**L**
ALT	**L**	**L**		**L**	**L**	**L**	**L**	**L**	**L**
GammaGT	**L**	**L**		**L**	**L**	**L**	**L**	**L**	**L**
Glucose	**L**	**L**		**L**	**L**	**L**	**L**	**L**	**L**
PCR	**L**	**L**		**L**	**L**	**L**	**L**	**L**	**L**
Sodium	**L**	**L**		**L**	**L**	**L**	**L**	**L**	**L**
Potassium	**L**	**L**		**L**	**L**	**L**	**L**	**L**	**L**
Urinalysis	**L**	**L**		**L**	**L**	**L**	**L**	**L**	**L**
VES	**L**								
CK		**L**			**L**	**L**		**L**	**L**
Total cholesterol		**L**			**L**	**L**		**L**	**L**
LDL Cholesterol		**L**			**L**	**L**		**L**	**L**
Triglycerides		**L**			**L**	**L**		**L**	**L**
Uric acid		**L**			**L**	**L**		**L**	**L**
Urea		**L**			**L**	**L**		**L**	**L**
Ferritin		**L**			**L**	**L**		**L**	**L**
Pregnancy test	**L**								
HIV, Hepatitis B, Hepatitis C, TB	**L**								
**Biological activity tests**									
T-regs		**MO(3)**			**MO (4)**	**MO**		**MO (5)**	**MO**
Lymphocytes phenotype		**MO**			**MO (4)**	**MO**		**MO (5)**	**MO**
S6RP phosphorilation		**MO**			**MO (4)**	**MO**		**MO (5)**	**MO**
Inflammasome		**MO**			**MO (4)**	**MO**		**MO (5)**	**MO**
Rapamycin blood dosage			**Only at W1, L**	**L**	**L**	**L**			
**Peripheral biomarkers**									
Vitamin D		**L**			**L**	**L**		**L**	**L**
Folates		**L**			**L**	**L**		**L**	**L**
Neurophilaments		**MO**			**MO**	**MO**		**MO**	**MO**
**CSF tests**									
Rapamicyn dosage						**L**			
Neurophilaments		**MO**				**MO**			

(6) Erythrocytes, Hematocrit, Hemoglobin, Platelets, Leucocytes, Neutrophils, Eosinophils, Basophils, Lymphocytes, Monocytes,
(7) L= local laboratory of the centre
(8) MO= Laboratory of Immunology (Prof. Cossarizza) Of the University of Modena and Reggio Emilia
(9)Only at week 8
(10)Only at week 30

6.3 WORK PLAN

This multicentre study involves 10 centers:

1. ALS Center, Nuovo Ospedale Civile S. Agostino Estense, Azienda Ospedaliero Universitaria di Modena (Coordinating Centre)
2. Prof. Andrea Cossarizza, University of Modena and Reggio Emilia
3. Prof. Roberto D'Amico, University of Modena and Reggio Emilia
4. Dr. Letizia Mazzini, ALS Center, University of Novara
5. Dr. Claudia Caponnetto, ALS Center, University of Genova
6. Prof. Adriano Chiò, ALS Center, University of Torino
7. Dr. Eleonora Dalla Bella, IRCCS Foundation "Carlo Besta" Neurological Institute
8. Dr. Christian Lunetta, Centro NEMO, Milano
9. Dr. Kalliopi Marinou, ALS Center, "S. Maugeri" Foundation, Milano
10. Dr. Gianni Sorarù, ALS Center, University of Padova

ALS is a rare disease with fast progression and no effective treatment, which requires a solid clinical approach and a multicenter randomized double blind design for clinical trials conduction. Our Consortium includes centres that have a longstanding history on ALS management, and are referral Centres for ALS care following >150 patients/year each. They share an established collaboration in the field of ALS, in clinical management of patients, in the realization of clinical trials (among which EPOS trial, coordinated by Dr Lauria) and in clinical research through the ITALSGEN Consortium (coordinated by Prof Chiò). Dr Mandrioli is the chief a regional ALS reference centre for Emilia Romagna Region (ERR); she created and has been coordinating the ERR Registry for ALS (>4 million pop). Prof Chiò is an internationally recognized scientist in the field of ALS. He is the Chief of the Turin ALS centre, he created the Piedmont and Valle d'Aosta Registry (>4 million pop.), the ITALSGEN Consortium, and coordinates different research lines. DrCaponnetto is the chief of ALS reference centre for Liguria Region and has been coordinating the Liguria Registry for ALS (1.6 million pop) and the trial "Treatment of ALS with cyclophosphamide followed by autologous haematopoietic stem-cell transplantation: an open label phase I/IIa study". Dr Marinou works at IRCCS Fondazione S. Maugeri in Milan, a reference Centre for ALS clinical management and research, and has great experience in ALS rehabilitation, ALS care and clinical trials conduct. Dr Lunetta is the chief of ALS care and research of the Italian Centre for Neuromuscular diseases "NEMO" in Milan, a nationwide referral Centre for ALS management and research. Dr Dalla Bella works with Dr Lauria at ALS Centre and focus on MND clinical research and management of clinical trial. Dr Mazzini focused his research on stem cells; that centrecoordinates with Dr Sorarù the trial "Human Neural Stem Cell Transplantation in ALS".
The analysis of biomarkers will be centralized and performed in the laboratory of Prof Cossarizza, who has large experience in cell analysis and is internationally renowned for the use of flow cytometry for studies on several human diseases.
Prof D'Amico is a statistician with considerable experience on RCT project and management, and on data analysis. He has been involved in planning the study design and will be in charge of data management and analysis as well as dissemination of results

According to different roles of partners in the study, the work plan will be divided into 4 WPs: WP1 (study preparation); WP2 (RCT conduction); WP3 (laboratory studies on biomarkers), WP4 (data extraction, statistical analyses and result presentation).

6.3.1 WP1: Management and Project Coordination

WP1 has been designed to manage and coordinate the proposed research project, through monitoring activities of all centres, facilitating communication, promoting exchange of ideas and methodological approach, stimulating the analysis and the integration of results.

Kick off meeting and regular meetings with partners

The PI will organize the kick off meeting, regular meetings with partners, and on time correspondence of the trial activities: a first meeting will be organized to share the study protocol and rules, to discuss methodology and possible pitfalls of the study. Participating Centres will discuss and contribute to finalize the protocol, and will participate to investigator meetings.

Regular meetings will be organized, also using videoconferences to limit expenses for travels, and also to ensure the maximum participation of all partners at meetings.

During regular meetings exchange of ideas and methodological approach will be ensured as well as problem discussion and solving, and sharing of the work in progress.

The final meetings will be devoted to analysis and discussion of results.

Ethical Committee requirements

The approval is expected to be obtained by month 4. National Authorities requirements for pharmacological studies will be addressed together with submission to Ethical Committees of the Participating Centres. An independent Contract Research Organization (CRO) will be selected for study monitoring. A Trial Manager will oversee the conduct of the study alongside the PI from the beginning of the study.

CRFs creation

Partner 2 (Statistics Unit, Prof. D'Amico, together with the coordinating centre will create, the eCRF based on the format of recently used eCRF for other RCT. The eCRF will be released at month 5-6 and tested by the coordinating Centre at month 6, to be therefore provided to each participating centre before the beginning of the study (month 6).

Establishment of an independent Data and Safety Monitoring Board (DSMB)

An independent DSMB will be established, to address safety and efficacy concerns that could arise during the study. This will be notified to the Ethic Committee of the coordinating centre. Reports including all relevant clinical data of patients will be sent by partner 2 to an independent DSMB for scheduled DSMB meetings (also through Skype).

The DSMB will evaluate clinical course in relation to safety of the first 10 patients enrolled and treated for 3 months; a report on benefit-risk ratio will be sent to the Ethic Committee of the coordinating centre before proceeding with the enrollment of the remaining 53 patients.

Then Partner 2 will send reports to the DSMB for scheduled DSMB meetings when 50% of patients have completed at least week 8 of treatment and subsequently after every 3 months.

6.3.2 WP2: RCT conduction

This study is a phase II randomized, double-blind, placebo-controlled, multicenter, clinical trial (RCT) to test Rapamycin in ALS patients. The study will include 63 ALS patients (EL Escorial Revised Criteria, sporadic and familial) with 1:1 allocation in 3 groups of 21 subjects each;

treatment will be double blinded, and will last 18weeks. Active treatment will include oral Rapamycin (R) at 1 mg/m2/d or 2 mg/m2/d. R levels will be regularly measured and revealed only to an independent monitor, who will make dosing advices to blinded caring neurologists to keep R levels from 4 to 12 ng/ml as well as corresponding sham dose adjustments in placebo group. Computerized randomization will be stratified by ΔFS (</≥0.7). Randomization time: 10 months. Treatment: 18 weeks. Post-treatment follow up: 36 weeks. Overall study duration: 24 months. The PI together with clinical partners will enrol patients; they will administer treatments/placebo (in double blind setting) and will be responsible for patients follow up and AE recording. They will follow patients with a multidisciplinary approach; they will record data on disease progression, procedure, side effects. They will manage with great attention every possible side effect due to the ongoing treatment. The partners will organize sample collection and shipping together with Partner 1, who will be responsible for management of all samples and immunological assessments.
Partner 2 will be in charge of computerized randomization, will contribute to trial coordination and to send data reports to an independent DSMB after 3 months of treatment of the first 10 patients enrolled; Partner 2 will then send reports to the DSMB for scheduled DSMB meetings after the 31st subject (50%) have completed at least week 8 of treatment and subsequently after every 3 months.
The following aims will be pursued:
1) to assess immunological changes induced by Rapamycin in ALS patients: blood collection will be performed before starting treatment, then at week 8-18-30 and 54. Fresh blood samples will be sent to University of Modena and Reggio Emilia Laboratory of Immunology and examined within 24 hours. The “immunological response” is defined as the proportion of patients exhibiting a Treg number (%) increase of at least 30% comparing baseline and treatment end between Rapamycin and placebo arm. Treg will be defined as CD4+CD25++CD127-FoxP3+ T cells. Other immunological changes to be examined will be changes in the phenotype of peripheral blood cells (T, B and NK cell activation and differentiation), changes in S6RP phosphorylation, pro-inflammatory cytokines and cytokines linked to TL proliferation and differentiation, inflammatory markers (molecular analysis of the inflammasome components and cytokine production by peripheral blood monocytes) comparing baseline and treatment end between Rapamycin and placebo arm.
2) To assess safety and tolerability of Rapamycin in ALS patients: all patients will be accurately screened for any possible contraindication to active treatment. Safety and tolerability will beassessed as follows:

a) in the first 10 patients enrolled clinical and biological examination will be assessed every week for the first month and then every two weeks until week 18. At every scheduled visit (Week 0-1-2-3-4-6-8-10-12-14-16-18-24-30-42-54) patients will be clinically monitored with particular attention to all possible side effects (total and severe adverse events): respiratory, hepatobiliary, renal, cardiac toxicity, allergic reactions, edema, poor wound healing, increased blood pressure, pain (including stomach and joint pain), nausea, diarrhoea, headache, fever, urinary tract infection, increased serum levels of cholesterol and triglycerides, increased urine protein levels, anaemia, low platelet count. Patients will be monitored for increased risk of infections, and of skin cancer or lymphoma. Analysis will be performed considering treatment period and observational period for early and late AE. To monitor early and late AE clinical examinations and routine laboratory work will be performed before taking R/placebo, then at week 1,2,3,4,6,8,10,12,14,16,18,24,30,42,54, and will include blood cell count, liver and renal function, inflammation markers, urine examination (see flow chart 2a). Other routine laboratory tests (e.g. serum cholesterol and triglycerides, see flow chart 2a) will be done at week 2,4,6,8,12,18,24,30,42,54, and drug dosage (Rapamycin blood dosage) at week 1-2-4- 8-12-18. Moreover, a phone call will be done every week of treatment to assess safety.

Via Giardini, 1355
Baggiovara
41126 Modena
tel.: 059396111
fax.: 0593962409

b) For the following 53 patients: at every scheduled visit (Week 0-2-4-6-8-12-18-24-30-42- 54) patients will be clinically monitored with particular attention to all possible side effects (total and severe adverse events): respiratory, hepatobiliary, renal, cardiac toxicity, allergic reactions, edema, poor wound healing, increased blood pressure, pain (including stomach and joint pain), nausea, diarrhoea, headache, fever, urinary tract infection, increased serum levels of cholesterol and triglycerides, increased urine protein levels, anaemia, low platelet count. Patients will be monitored for increased risk of infections, and of skin cancer or lymphoma. Analysis will be performed considering treatment period and observational period for early and late AE. To monitor early and late AE clinical examinations and routine laboratory work will be performed before taking R/placebo, then at week 2,4,6,8,12,18,24,30,42,54, and will include blood cell count, serum cholesterol and triglycerides, liver and renal function, C reactive protein, urine examination, and drug dosage (Rapamycin blood dosage at week 1-2- 4-8-12-18). Moreover, a phone call will be done every week of treatment to assess safety.

AE reporting will be performed according to "ICH guidance for Clinical Safety Data Management: Definitions and Standards for Expedited Reporting"

Tracheostomy free survival in patients treated with active treatment and placebo will also be assessed at study end.

An independent DSMB will be established and notified to the Ethic Committee of the coordinating centre, to address safety or other concerns that may arise during the study, with frequent meetings.

3) To assess the best risk benefit ratio for Rapamycin doses: based on the occurrence of AEs in each group (1mg or 2mg) or other clinically significant safety evaluations, it will be decided which dose of Rapamycin has the best risk/benefit ratio to conclude for further studies.

4) To assess biomarkers changes (including CSF and peripheral biomarkers i.e. creatinine, albumin, vitamin D, plasma/CSF neurofilament heavy/light chain protein (NF)) comparing baseline and treatment end between Rapamycin and placebo arm. The non-routine laboratory studies will be performed at baseline and at week 8-18-30-54; CSF will be taken at baseline and at 18 weeks.

5) To assess the capability of R to cross the blood brain barrier: given the large natural structure of R, to understand whether sufficient levels of R can be found in the CNS, we will dose R in the CSF of patients at week 18 (in the toxicology laboratory that will dose R in the blood using high-performance liquid chromatography (HPLC) with mass spectrometry (MS) (LC-MS/MS)).

6.3.3 WP3: Laboratory Assessment (Drug targets and Biomarkers)

Immunological studies will be performed at the time-points indicated in WP2 (at baseline and week 8-18-30-54)

The following aims will be pursued:

1) To identify the changes in the absolute number, percentage and phenotype of Tregs, comparing baseline and study end between different treatment doses and placebo arm. Treg will be defined as CD4+CD25++CD127-FoxP3+ T cells. Method: polychromatic flow cytometry (PFCM), using a combination of mAbs anti-CD3,-4,-25,-127,-FoxP3 along with mAbs that recognize markers of activation (HLA-DR,CD38) and homing (CXCR3) expressed by Treg.

2) To identify changes in Treg activation and homing capabilities along with several sub-populations of CD4+ and CD8+ T cells, as well as various subsets of B and NK cells in the two groups. Methods: PFCM for the detection of differentiation and activation of different populations of T cells (naïve, central memory, effector memory, terminally differentiated) with specific mAbs (anti-CD3, CD4, CD8, CD45RA, CCR7, CD38, CD95, CD127), populations of B cells (B1 cells,

Via Giardini, 1355
Baggiovara
41126 Modena
tel.: 059396111
fax.: 0593962409

naïve, unswitched memory, switched memory B cells) by using mAbs anti-CD5, CD19, CD27, CD38, CD95, sIgD), and populations of NK cells (expressing different activating and inhibitory receptors) by using mAbs anti-CD3, CD16, CD56, CD57, CD62L, HLA-DR, KIR2DL1, KIR2DL2. 3)To identify changes in mTOR activity through the cytofluorimetric detection of the phosphorylated form of S6RP (a target of mTOR), in T, B and NK cells. Methods: by using a mAb, which is specific for the phosphorylated form of the protein, in T, B and NK cells, identified as in T3.2.

4)To identify changes in levels of pro-inflammatory cytokines and cytokines linked to T cell proliferation and differentiation. Methods: isolation of plasma from blood samples, and quantification of the levels of pro and anti inflammatory cytokines (TNF-alpha, IL-1, IL-6, IL-10, IL-12, IL-17, IL-18, IFN-gamma, TGF-beta) related to Treg activity, to the relative percentage and activation of T, B and NK T cell subsets, or to inflammasome activation in monocytes.

5) To identify changes in the activation of the inflammasome system in monocytes. Method: Real Time and Digital PCR assays for monitoring the expression levels of genes crucial for inflammasome assembly (such as NLRP3, AIM2 and several others) and for the expression of IL- 1beta.

6) To assess biomarker changes, namely plasma/CSF neurofilament heavy/light chain protein (NF). Methods: an aliquot of the withdrawn blood will be processed and stored at -80°C together with CSF samples until NF measurements; CSF sample collection and analysis to quantify NF will be performed before and after treatment.

The cytofluorimetric identification of the aforementioned cell populations will be performed with the most advanced polychromatic flow cytometry technologies, for the identification of up to 10 antigens of interest per cell. In particular, we will use a novel 16 parameter acoustic focusing AttuneNxT flow cytometer (Thermo Fisher) able to identify up to 35,000 cells/second. Staining of cells with different panels of monoclonal antibodies will be performed by using freshly collected whole blood. Panels are routinely used in this laboratory for the recognition of Tregs and activated Tregs, as well as populations of T, B and NK cells. These techniques and analytical approaches are well standardized and largely used in the laboratory of Immunology, as well evidenced by several publications in which these and other flow cytometry analyses have been employed.

Partner 3,4,5,6,7,8,9 and coordinating Unit will be responsible for sample collection before taking Rapamycin/placebo, then at scheduled visits. Partner 1 will receive all biological samples from partners 3,4,5,6,7,8,9, the day after (within 9 a.m.) the blood sample (which will be withdrawn in the afternoon); they will process blood and CSF, and perform all of the aforementioned analysis leading to assess immunological profile of treated and not-treated patients.

6.5.4 WP4: Analysis of data and results dissemination

Data extraction: The investigators will collect data by entering them directly into the trial database, through electronic case report forms ad hoc developed. At the end of the RCT, database will be locked and an expert Statistician (Prof. R. D'Amico) will check the database and extract all data for statistical analyses.

Statistical analysis: The analyses will be performed by intention-to-treat. Per protocol analysis will be carried out after excluding non-compliers (patients who will take <80% therapy) and drop-out for any reason. Statistics will be tabulated by treatment arm and time. Results with p-values less

than 0.05 will be considered as statistically significant. Statistical analyses will be carried out using Stata 12 software (Stata Corporation, College Station, Texas). Randomization and statistical analysis will be performed by independent persons who are not involved with patients.

Presentation and dissemination of results.

Results will be presented as appropriate effect estimates (mean differences, relative risks, hazard ratios) along with their relative 95% confidence intervals. The report on final results will be first communicated to AriSLA.
The PI and all the partners will guarantee the dissemination and exploitation of the scientific results within the consortium and externally (international conferences, publications, etc).
Dissemination of results will include national and international neurological and ALS meetings and workshops. We will organize the participation to conferences, where we will present our work, allowing to the units to achieve visibility.
We will schedule, plan and coordinate the writing of scientific papers (so that delays in manuscript preparation will be avoided). Results will be published in peer-reviewed international journals.
Any publication of results will be previously communicated to ARISLA and will report ARISLA contribution according to ARISLA Policy on Communication and Dissemination.

7. STUDY POPULATION

Probable laboratory-supported, probable, definite ALS according to revised El Escorial criteria (sporadic and familial).
The study will be performed on 63 patients enrolled in 3 groups of 21 subjects (Rapamycin 1 mg/m2/day, Rapamycin 2 mg/m2/day, placebo).

7.1 INCLUSION CRITERIA

-Patient diagnosed with a laboratory supported , clinically “probable” or “definite” amyotrophic lateral sclerosis according to the Revised El Escorial criteria (Brooks, 2000)
-Familial or sporadic ALS
-Female or male patients aged between 18 and 75 years old
-Disease duration from symptoms onset no longer than 18 months at the screening visit
-Patient treated with a stable dose of Riluzole (100 mg/day) for at least 30 days prior to screening
-Patients with a weight > 50 kg and a BMI ≥18
-Patient with a FVC (Forced Vital Capacity) equal or more than 70 % predicted normal value for gender, height, and age at the screening visit
-Patient able and willing to comply with study procedures as per protocol
-Patient able to understand, and capable of providing informed consent at screening visit prior to any protocol-specific procedures
-Use of effective contraception both for males and females

7.2 EXCLUSION CRITERIA

-Prior use of Sirolimus
-Prior allergy/sensitivity to Sirolimus or macrolides
-Any medical disorder that would make immunosuppression contraindicated, including but not limited to, acute infections requiring antibiotics, patients with known diagnosis of HIV, TBC, hepatitis B or C infection or history of malignancy
-Severe comorbidities (heart, renal, liver failure), autoimmune diseases or any type of interstitial lung disease
-White blood cells<4,000/mm³, platelets count<100,000/mm³, hematocrit<30%
-Patient who underwent non invasive ventilation, tracheotomy and /or gastrostomy
-Women who are pregnant or breastfeeding
-Participation in pharmacological studies within the last 30 days before screening
-Patients with known SOD1 mutation or with FALS and family members carrying SOD1 mutation

7.3 PATIENT IDENTIFICATION

All patients who have signed the informed consent document will receive a patient number that serves to identify the patient throughout the study.
Patients will be identified by a numeric code including the centre number, followed by a chronological inclusion number for that centre (XXX-YY), the first two letters of the last name and first name.
All subjects will receive a unique subject identification number at screening visit when signing the informed consent by the owner and before any study procedures are performed. This number will be used to identify the subject throughout the study and must be used on all study documentation related to that subject. The subject identification number must remain constant throughout the entirestudy; it must not be changed at the time of enrolment, or randomisation.

8. STUDY TREATMENT

8.1. STUDY TREATMENT DEFINITION

8.1.1. Investigational Medicinal Product (IMP)

The IMP is defined as "a pharmaceutical form of an active substance or placebo being tested or used as a reference in a clinical study, including products already with a marketing authorization but used or assembled (formulated or packaged) in a way different from the authorized form, or when used for an unauthorized indication, or when used to gain further information about the authorized form."
In this trial, the investigational product is rapamycin and its matching placebo.
Rapamycin is supplied free of charge to the study investigators by Pfizer. Rapamycin is supplied as 1 mg tablets packaged in polyethylene bottles.
Inactive ingredients are sucrose, lactose, polyethylen glycol 8000, calcium sulphate, microcrystalline cellulose, povidone, pharmaceutical glaze, talc, titanium dioxide, magnesium stearate, poloxamer 188, polyethylen glycol 20000, glyceryl monoleate, carnauba wax, dl-alpha- tocopherol.
Placebo is supplied in tablets identical to the rapamycin ones, with the same composition except for active ingredient
Riluzole:
Riluzole is not considered as IMPs in this study and as such will not be provided by the sponsor.
The product should be prepared, handled, used and stored according to standard practices and the Summary of Product Characteristics (SPC).

8.1.2. Concomitant Treatment

All medications taken by the patients at the onset of study and all medication given in addition to the IMP during the study are regarded as concomitant medications.

8.2. INVESTIGATIONAL MEDICINAL PRODUCT

8.2.1. Packaging and Labelling

All medications to be used in this study will have been manufactured, tested, and released according to current GMP guidelines.
Rapamycin is supplied as 1 mg non-divisible tablets triangle-shaped orange coated tablets. Inactive ingredients are sucrose, lactose, polyethylen glycol 8000, calcium sulphate, microcrystalline

cellulose, povidone, pharmaceutical glaze, talc, titanium dioxide, magnesium stearate, poloxamer 188, polyethylen glycol 20000, glyceryl monoleate, carnauba wax, dl-alpha-tocopherol.
The 1 mg tablets are packaged by 30 units in bottles closed with a childproof cap.
The IMP will be packaged and labelled according to current GMP guidelines, GCP guidelines, and national legal requirements. The package given to the patient will have a tear-off part. When the IMP is dispensed to the patient, the investigator or pharmacist (if applicable) will remove the tear- off part of the label and attach it to the respective study documents.

8.2.2. Shipment, Storage Conditions and Accountability

The investigator or pharmacist (if applicable) will receive numbered treatments. The investigator/pharmacist is responsible for a safe and proper handling and storage of the IMP at the investigational site. The IMP must be stored in a locked facility with restricted access to the investigator/pharmacist and authorized personnel. The investigator must ensure that the IMP is administered only to patients enrolled in this study. The IMP has to be stored at room temperature (between 15°C and 25°C). Temperature logs should be kept updated by the investigator or the pharmacist to document adequate storage during the course of the study.
The IMP must not be used outside the context of this study protocol. The investigator or authorized staff are obliged to document the receipt, dispensation, and return of all IMPs received during this study.
Records on receipt, use, return, loss, or other disposition of IMPs must be maintained. The investigator or, if applicable, the pharmacist must sign the receipt forms. Records on IMP delivery to the site, the inventory at the site, the use by each patient, and the return to the sponsor must be maintained by the investigator and/or another appropriately trained individual at the investigational site. These records will include dates, quantities, batch numbers, and the unique code numbers assigned to the IMP and the patients. The investigators must maintain records documenting that the patients were provided with their respective doses specified in the protocol. Furthermore, they should reconcile all IMPs received. It is the responsibility of the investigator to give reasons for any discrepancies in IMP accountability. This process will be monitored by a CRO (Clinical Research Organization) during the study.
All remaining IMPs, used and unused, shall be collected and returned for destruction at the end of the study. An authorized company (Euromed Clinical Supply Services srl (ECLISSE)) will be responsible for investigational medicinal product (IMP) manufacture and logistics services, including preparation of randomization list, envelopes, and code breaking cards, packaging and labelling, storage, shipment and destruction of the product.

8.2.3. Patient Compliance

Patients will be instructed to bring their used and unused IMP at each visit. Compliance will be assessed by the investigator through counting the remaining tablets returned by the patient. The compliance at each visit should not be lower than 80% and higher than 120%. The investigator will decide on a clinical basis whether or not to keep the patient in the study.
Compliance of riluzole will be assessed too.

8.2.4. Study Treatment Administration

Patients enrolled will be randomised in 3 groups:

- Group 1: patients will receive Rapamycin 2 mg/m2/day and riluzole (21 patients)
- Group 2: patients will receive Rapamycin 1 mg/m2/day and riluzole (21 patients)
- Group 3: patients will receive placebo and riluzole (21 patients)

Treatment will be double blinded to patients and physicians, and will last 18 weeks.

Subjects enrolled will receive a total daily dose of 2 or 1 mg/m2 Rapamycin, or a matching placebo, to be taken before breakfast as indicated in the tables below.

Study treatment administration

Study treatment daily dose of 2 mg/m2 will be administered as indicated in Table 1

Table 3: Dose of study treatment (mg) to be administered according to patient's body surface area (2 mg/m2/day)

	2 mg/m2	
Patient's body surface area (BSA*)	Daily dose (mg)	Morning
<1.5 m2	3 mg	3 tablets
1.51-1.75 m2	3 mg	3 tablets
1.76-2.0 m2	4 mg	4 tablets
>2.0 m2	4 mg	4 tablets

*BSA was calculated from the Mosteller formula: BSA=√(Height*Weight)/3600

Table 4: Dose of study treatment (mg) to be administered according to patient's body surface area (1 mg/m2/day)

	1 mg/m2	
Patient's body surface area (BSA*)	Daily dose (mg)	Morning
<1.5 m2	1 mg	1 tablets
1.51-1.75 m2	2 mg	2 tablets
1.76-2.0 m2	2 mg	2 tablets
>2.0 m2	2 mg	2 tablets

*BSA was calculated from the Mosteller formula: BSA=√(Height*Weight)/3600

Steps for dose reduction

According to the initial dose of Rapamycin or matching placebo, the steps for the dose reduction are as follows:

Starting dose	1st dose reduction	2nd dose reduction	3rd dose reduction
2 mg/m2/day	1 mg/m2/day	1 mg/day	STOP

1 mg/m2/day	1 mg/day	STOP	

Table 5: Dose of study treatment to be administered according to patient's BSA, after a dose reduction to 1 mg/m2/day (randomization dose: 2 mg/m2/day)

	1 mg/m2	
Patient's body surface area (BSA*)	Daily dose (mg)	Morning (fast)
<1.5 m2	1 mg	1 tablets
1.51-1.75 m2	2 mg	2 tablets
1.76-2.0 m2	2 mg	2 tablets
>2.0 m2	2 mg	2 tablets

Table 6: Dose of study treatment to be administered according to patient's BSA, after a dose reduction to 0.5 mg/m2/day (randomization dose: 1 mg/m2/day or second dose reduction)

	0.5 mg/m2	
Patient's body surface area (BSA*)	Daily dose (mg)	Morning (fast)
<1.5 m2	Not possible	
1.51-1.75 m2	1 mg	1 tablets
1.76-2.0 m2	1 mg	1 tablets
>2.0 m2	1 mg	1 tablets

Procedure in case of missed or vomited doses of study treatment tablets:

-In case the morning dose has been missed, it can be taken until 2 pm. on the same day. Should it be later than 2 pm, the missed dose will not be made up and study treatment will be resumed at the next morning.

-Should the patient vomit within 10 minutes after tablets intake, another dose should be taken.

-Should the patient vomit later than 10 minutes following the last study treatment dose intake, study treatment will be resumed at the next morning, and the last dose will not be replaced.

8.3. CONCOMITANT MEDICATIONS

All medications taken by the patients at the onset of study and all medication given in addition to the IMP during the study are regarded as concomitant medications.
Patients are not allowed to enter the study if they receive any prohibited concomitant medication or medication in a dosage not allowed and which cannot be discontinued or reduced.

Via Giardini, 1355
Baggiovara
41126 Modena
tel.: 059396111
fax.: 0593962409

8.3.1. Mandatory Concomitant Treatment

Patients must have been treated for a minimum of 1 month with a stable dose of riluzole (100 mg/day) at baseline. Safety issues related to riluzole should be managed according to usual practice.

8.3.2. Prohibited Concomitant Treatment

Rapamycin is known to be a substrate for both cytochrome P-450 3A4 (CYP3A4) and p- glycoprotein (P-gp). Inducers of CYP3A4 and P-gp may decrease Rapamycin concentrations whereas inhibitors of CYP3A4 and P-gp may increase Rapamycin concentrations.

Strong Inducers and Strong Inhibitors of CYP3A4 and P-gp
Concomitant use of strong inducers (e.g., rifampin, rifabutin) is not recommended, whereas it is forbidden concomitant use of strong inhibitors (e.g., ketoconazole, voriconazole, itraconazole, erythromycin, telithromycin, clarithromycin) of CYP3A4 and P-gp.
Alternative agents with lesser interaction potential with Rapamycin should be considered.

Table 7: Drugs that strongly inhibits CYP3A4

Amiodarone	Ketoconazole
Anastrozole	Metronidazole
Azithromzcin	Mibefradil
Cannabinoids	Miconazole
Cimetidine	Nefazodone
Clarithromycin	Nelfinavir
Clotrimazole	Nevirapine
Cyclosporine	Norfloxacin
Danazol	Norfluoxetine
Delavirdine	Omeprazole
Dexamethasone	Oxiconazole
Diethyldithiocarbamate	Paroxetine (weak)
Diltiazem	Propoxyphene
Dirithyromycin	Quinidine
Disulfiram	Quinine
Entacapone (high dose)	Quinupristine and dalfopristin
Erythromycin	Ranitidine
Ethinyl estradiol	Ritonavir
Fluconazole	Saquinavir
Fluoxetine	Sertindole

Fluvoxamine	Sertraline
Gestodene	Troglitazone
Grapefruit juice	Troleandomycin
Indinavir	Valproic acid
Isoniazid	

Table 8 Drugs that strongly induce CYP3A4

Carbamazepine
Dexamethasone
Ethosuximide
Glucocorticoids
Griseofulvin
Phenytoin
Primidone
Progesterone
Rifabutin
Rifampin
Nafcillin
Nelfinavir
Nevirapine
Oxcarbazepine
Phenobarbital
Phenylbutazone
Rofecoxib (mild)
St John's wort
Sulfadimidine
Sulfinpyrazone
Troglitazone

Vaccination

Immunosuppressants may affect response to vaccination. Therefore, during treatment with Rapamycin, vaccination may be less effective.
The use of live vaccines should be avoided; live vaccines may include, but are not limited to, the following: measles, mumps, rubella, oral polio, BCG, yellow fever, varicella, and TY21a typhoid.

Grapefruit Juice

Via Giardini, 1355
Baggiovara
41126 Modena
tel.: 059396111
fax.: 0593962409

Because grapefruit juice inhibits the CYP3A4-mediated metabolism of Rapamycin, it must not be taken with or be used for dilution of Rapamycin.

8.3.3. Treatment to be given with High Caution

Because of the inherent risk of either reduced activity or enhanced toxicity of the concomitant medication and/or Rapamycin, drugs known to interact with the same cytochrome P450 (CYP450) isoenzymes (3A4) as study treatment should be used with caution. Patients using concomitant medications known to be metabolized by these CYP450 enzymes will not be excluded from the study. However, the patients must be carefully monitored for potential of toxicity due to individual concomitant medication. Should an event occur, a blood sample should be obtained for analysis of this medication and/or Rapamycin whenever possible.
The dosage of Rapamycin and/or the co-administered drug may need to be adjusted.

Drugs that could increase Rapamycin blood concentrations:

Bromocriptione, cimetidine, cisapride, clotrimazole, danazol, diltiazem, fluconazole, HIV-protease inhibitors (e.g., ritonavir, indinavir), metoclopramide, nicardipine, troleandomycin, verapamil

Drugs and other agents that could decrease Rapamycin concentrations:

Carbamazepine, phenobarbital, phenytoin, rifapentine, St. John's Wort (*Hypericum perforatum*)

Drugs with concentrations that could increase when given with Rapamycin:

Verapamil

8.4 RANDOMIZATION

Eligible pts will be randomised strictly sequentially, according to a randomization list prepared by the Biostatistician of the University of Modena.
All patients will receive a unique patient identification number at screening visit when signing the informed consent and before any study procedures are performed. This number will be used to identify the patient throughout the study and must be used on all study documentation related to that patient. The patient identification number must remain constant throughout the entire study; it must

not be changed at the time of enrolment, or randomisation.

63 patients will be randomized in the following three groups:

- 21 patients will receive Rapamycin 2 mg/m2/day + riluzole
- 21 patients will receive Rapamycin 1 mg/m2/day + riluzole
- 21 patients will receive placebo + riluzole

Stratification
Randomization will be performed on line. Patients will be stratified according to ΔFS (</≥0.7)(Kimura,2006), with 1:1 allocation in 3 treatment arms (Rapamycin 1mg/m2/day; Rapamycin 2mg/m2/day, placebo). The investigator will randomize patients directly on line. Treatment must begin within 14 days from randomization. In case of discontinuation from the study, the randomization number will not be reused. Enrolment time: 10 months.

8.5. BLINDING PROCEDURES

Eligible patients will be randomized by means of a computerized central randomization system. The automated system will assign the appropriate IMP for each patient. The Statistics Unit based at the University of Modena (partner 2) will supply the investigators with user guides for the automated system.
This study is a double-blind study. The investigator will be provided with technical options and password information to selectively break the code for an individual patient by telephone, facsimile transmission, or through electronic message transfers.
The premature breaking of the code should be confined to emergency cases in which knowledge of the administered drug is necessary for adequate treatment. Whenever possible, the Statistics Unit based at the University of Modena should be contacted before breaking the blinded emergency code. Should any code be broken, the respective patient will be withdrawn from further participation in the study and a written explanation must be given by the investigator.

8.6. MANAGEMENT OF TOXICITY FOR RAPAMYCIN AND MATCHING PLACEBO

8.6.1. Call from site to patient once a week for the first 2 months of treatment

During the treatment, the center will call the patient every week to verify compliance with the drugs and ask questions to detect any signs which might be due to an underlying infection or other possible SAE.

Via Giardini, 1355
Baggiovara
41126 Modena
tel.: 059396111
fax.: 0593962409

8.6.2. Procedures to manage adverse reaction potentially related to study treatment

Study treatment refers to Rapamycin and matching placebo. Toxicity related to riluzole should be managed according to the usual clinical practice.

Adverse Events

Adverse Events (AE) (serious and non serious AE), Adverse Drug Reaction (ADR), Unexpected ADR, will be defined accordingly to "ICH guidance for Clinical Safety Data Management: Definitions and Standards for Expedited Reporting".

All AE occurring between the first study-related procedure and the last study-related procedure will be reported. Those meeting the definition of Serious AE (SAE) will be reported in an ad hoc SAE Form; they will be reported to coordinating centre within 24 hours. All AE will be recorded in the eCRF with a diagnosis (whenever possible), and together with investigator's opinion concerning the relationship of the AE to study treatment. The coordinating Centre will be responsible for appropriate AE reporting to the regulatory authorities; investigators will be responsible for reportingto appropriate Ethic Committee. In case of death a clinical report will be prepared by the caring investigator together with SAE form; in case of autopsy, autopsy report will be added to study documentation.

Safety Data Collection will include classification of the event (serious or non-serious AE), description of signs or symptoms, diagnosis (where possible), onset and stop (date and time), intensity, correlation between the study agent and the adverse event as follows (probable, possible, not related, unknown), action taken (none; change in treatment administration; drug treatment required; non-drug treatment required; hospitalisation or prolonged hospitalisation; diagnostic or clinical test(s); discontinuation from the study), subject outcome.

Adverse reactions reported with Rapamycin

The following adverse events have been reported during the use of Rapamycin, mainly in transplanted patients and in association with other drugs.

Table 9: adverse reactions reported with Rapamycin use

Systems involved	Very common (>10%)	Common (1-10%)	Uncommon (0.1-1%)	Rare (<0.1%)
**Respiratory**	Dyspnea, upper respiratory infection, pharyngitis	Pneumonia, epistaxis, pleural effusion, epistaxis	Pulmonary hemorrhage	Alveolar proteinosis
**Metabolic**	Hypertriglyceridemia, hypercholesterolemia, hypokalemia, hypophosphatemia, hyperglycemia	Abnormal healing, increased LDH, hypokalemia, diabetes mellitus		
**Cardiovascular**	Peripheral edema, hypertension, chest pain, edema, lymphocele	Venous thromboembolism (including pulmonary embolism, deep venous thrombosis), tachycardia	Pericardial effusion, lymphedema	Pericardial effusion
**Gastrointestinal**	Constipation,	Stomatitis		

	abdominal pain, diarrhea, nausea, vomiting, dyspepsia			
**Hematologic**	Anemia, thrombocytopenia, blood lactate dehydrogenase increased, blood creatinine increased	Thrombocytopenic purpura/hemolytic uremic syndrome, leukopenia, neutropenia, aspartate aminotransferase increased, alanine aminotransferase increased	Pancytopenia	Capillary leak syndrome
**Genitourinary**	Urinary tract infection	Pyelonephritis, decline in renal function (creatinine increased) in long-term combination of cyclosporine with this drug, ovarian cysts, menstrual disorders, proteinuria		Azoospermia
**Musculo-skeletal**	Arthralgia	Bone necrosis		
**Nervous system**	Headache	Tremor, insomnia		
**Dermatologic**	Acne, rash	Herpes zoster, herpes simplex	Thrombotic thrombocytopenic purpura/hemolytic uremic syndrome, leukopenia, melanoma, squamous cell carcinoma, basal cell carcinoma	
**Renal**	Creatinine increased	Nephrotic syndrome		Focal segmental glomerulo-sclerosis, BK virus associated nephropathy, nephrotic syndrome, higher serum creatinine levels, lower glomerular filtration rates
**Hepatic**		Liver function tests abnormal	Hepatic failure, hepatic artery thrombosis	
**Hypersensitivity**				Hypersensitivity reactions, including anaphylactic/ anaphylactoid

				reactions, angioedema, exfoliative dermatitis, and hypersensitivity vasculitis
**Oncologic**		Skin cancer, lymphoma/post-transplant lymphoproliferative disorder<br>Frequency not reported: Hepatocellular adenoma and carcinoma, testicular adenoma		
**Immunologic/Infections**		Sepsis, pneumonia, pyelonephritis, herpes simplex, fungal, viral, and bacterial infections (such as mycobacterial infections, including tuberculosis, Epstein-Barr virus, CMV, and Herpes zoster), mycobacterial infections (including M tuberculosis), cytomegalovirus (CMV), Epstein-Barr virus		
**Other**	Fever, pain	Impaired healing		

Safety rules
For the following toxicities investigators must adhere to the following safety rules:

1. Respiratory toxicity, regardless of the causal relationship to study treatment
Cases of interstitial lung disease (including pneumonitis, bronchiolitis obliterans organizing pneumonia, and pulmonary fibrosis), some fatal, with no identified infectious etiology have occurred in patients receiving immunosuppressive regimens including Rapamycin. In some cases, the interstitial lung disease has resolved upon discontinuation or dose reduction. The risk may be increased as the trough Rapamycin concentration increases.
At each visit, respiratory symptoms are carefully checked by medical interview and clinical examination. In the case of suspect of an interstitial lung disease (including pneumonitis, bronchiolitis obliterans organizing pneumonia, and pulmonary fibrosis, pulmonary hemorrhage;

Via Giardini, 1355
Baggiovara
41126 Modena
tel.: 059396111
fax.: 0593962409

pleural effusion; alveolar proteinosis), a chest radiography, emogasanalysis, and spirometry should be performed, together with pulmonologist examination.
These cases should be distinguished from chronic respiratory insufficiency related to ALS.
In case of suspect of respiratory toxicity as above mentioned treatment should be discontinued and appropriate treatment started.

2. Metabolic toxicity, regardless of the causal relationship to study treatment
Hypertriglyceridemia, hypercholesterolemia, hypokalemia, hypophosphatemia, hyperglycemia, abnormal healing, increased LDH, hypokalemia, diabetes mellitus have been described in association with Rapamycin treatment.
Any patient should be monitored for these events and if detected, standard interventions such as diet, exercise, and lipid-lowering agents and other corrections should be initiated..
In case of metabolism and nutrition disorders of grade 1 and 2 (CTC-AE classification version 4) appropriate interventions (ad indicated by clinical practice) are suggested.
In case of Grade 3 (CTC-AE classification, version 4) treatment with Rapamycin should be discontinued until values normalization. Treatment will be resumed at the local investigator's judgment.
In case of Grade 4 (CTC-AE classification, version 4) treatment with Rapamycin should be definitely stopped.

3. Cardiovacular toxicity, regardless of the causal relationship to study treatment
At each visit, cardiac symptoms are carefully checked by medical interview and clinical examination.
In the case of suspect of a cardiac event ECG and troponin dosage should be performed, together with cardiological examination.
Pericardial effusion (including hemodynamically significant effusions and tamponade) should be considered in the differential diagnosis and echocardiography may be necessary.
Rapamycin has been associated with the development of angioedema. The concomitant use of Rapamune with other drugs known to cause angioedema, such as ACE-inhibitors, may increase the risk of developing angioedema.
In case of cardiovascular toxicity disorders of grade 1 and 2 (CTC-AE classification version 4) appropriate interventions (ad indicated by clinical practice) are suggested and treatment discontinued until resolution. In case of duration of the event > 2 week treatment should be definitely discontinued.
In case of Grade 3 or 4 (CTC-AE classification, version 4) treatment with Rapamycin should be definitely stopped.

3. Gastrointestinal toxicity, regardless of the causal relationship to study treatment
Nausea - vomiting, regardless of the causal relationship to study treatment
- -In case of nausea or vomiting, anti-emetics are recommended according to the usual practice.
- -In case of severe nausea or vomiting, interrupt study treatment until return to baseline or mild intensity, then resume with a dose reduction (one step).

Diarrhea, regardless of the causal relationship to study treatment
- -In case of diarrhoea, anti-diarrhoeal are recommended according to usual practice.
- -In case of severe diarrhoea, interrupt study treatment until return to baseline or mild intensity, then resume with a dose reduction (one step).

Hepatobiliary Disorders – Hepatotoxicity, regardless of the causal relationship to study treatment

-In case of Grade 2 liver enzymes increase; i.e. transaminases (AST or ALT or both) increase ≤ 5 ULN, and/or in case of bilirubin increase ≤ 3 ULN, study treatment should be maintained (depending on local investigator's judgment).

-In case of Grade 3 liver enzymes increase; i.e. transaminases (AST or ALT or both) increase > 5 ULN and ≤ 20 ULN, and/or in case of bilirubin increase > 3 ULN and ≤ 10 ULN, study treatment should be interrupted until transaminases levels return to ≤3ULN and bilirubin level returns to ≤1.5 ULN. Hepatic surveillance tests will be performed every week. Then study treatment may be resumed with a dose reduction (one step) depending on local investigator's judgment.

-In case Grade 4 transaminases increase (AST or ALT or both >20 ULN or bilirubin >10 ULN), study treatment must be definitely discontinued.

4.Hematological toxicity, regardless of the causal relationship to study treatment

Neutropenia, regardless of the causal relationship to study treatment

-In case of absolute neutrophils count between 500 and 1000/mmc, study treatment will be interrupted until absolute neutrophils count has returned above 1.500/mmc, and then restarted at the same dose.

-If duration of neutropenia > 4 weeks, study treatment will be definitely discontinued

-In case of absolute neutrophils count < 500/mmc, study treatment will be definitely discontinued.

The investigator must inform the sponsor immediately (within 24 hours by fax using a Serious Adverse Event form) even if he/she considers the neutropenia as non-serious.

In case of associated fever, the patient must be hospitalized in a special unit.

In case of fever, oral ulceration, sore throat or infection, a complete blood count should be performed in order to check the neutrophil count. In case of neutropenia, the above mentioned rules should be applied.

In any case, all concomitant treatment potentially inducing neutropenia must be stopped

Anaemia, regardless of the causal relationship to study treatment

-In case of anaemia of grade 1 (CTC-AE classification version 4) observation and appropriate clinical procedures should be taken.

-In case of Haemoglobin between 8 and 10 g/dl (grade 2, CTC-AE classification version 4), study treatment will be interrupted until Haemoglobin level has returned above 10 g/dl, and then restarted at the same dose.

-If duration of anaemia > 8 weeks, study treatment will be definitely discontinued

-In case of Haemoglobin level < 8 g/dl (grade 3 or 4, CTC-AE classification version 4), study treatment will be definitely discontinued.

In any case, all concomitant treatment potentially inducing anaemia must be stopped

Thrombocytopenia, regardless of the causal relationship to study treatment

-In case of absolute platelets count between 30000 and 50000/mmc, study treatment will be interrupted until absolute platelets count has returned above 50000/mmc, and then restarted at the same dose.

-If duration of thrombocytopenia> 4 weeks, study treatment will be definitely discontinued

-In case of absolute platelets count < 30000/mmc, study treatment will be definitely discontinued.

In case of any bleeding (i.e. haematuria, epistaxis, etc) a complete blood count should be performed in order to check the platelets count. In case of thrombocytopenia, the above mentioned rules should be applied.

In any case, all concomitant treatment potentially inducing thrombocytopenia must be stopped

5. Genitourinary toxicity, regardless of the causal relationship to study treatment

In case of urinary infections appropriate treatment should be taken; in case of persistent or severe (grade 3 or 4, CTC-AE classification version 4) toxicity, treatment should be discontinued.

6. Renal toxicity, regardless of the causal relationship to study treatment

-In case of Grade 2 renal failure defined by the association of creatinine level > 1.5 and ≤ 3 ULN, and creatinine clearance > 30 mL/min, according to the CTC AE classificationversion 4.0, treatment should be stopped until return to normal or to baseline level, and then resume study treatment at the same dose accordingly to the local investigator's judgment.

-In case of Grade 3 renal failure defined by at least one of the following criteria: creatinine level > 3 and ≤6 ULN, or creatinine level >3 to ≤6 fold from baseline, or proteinuria > 3.5g/24hours, or creatinine clearance between 15 and 29 mL/min, interrupt study treatment until return to Grade 1 or to baseline, and then study treatment may be resumed with a dose reduction accordingly to the local investigator's judgment.

-In case of Grade 4 renal failure, study treatment must be definitely discontinued

7. Reproductive system and pregnancy

-If pregnancy is suspected during the study, study treatment must be immediately withheld until the result of a laboratory pregnancy test is available. Should pregnancy be confirmed, the patient must be withdrawn from study participation.

-Azoospermia has been reported with the use of Rapamune and has been reversible upon discontinuation of Rapamune in most cases.

8. Hypersensitivity Reactions

Hypersensitivity reactions, including anaphylactic/anaphylactoid reactions, angioedema, exfoliative dermatitis and hypersensitivity vasculitis, have been associated with the administration of Rapamycin.

In these cases treatment should be definitely discontinued.

9. Skin Toxicity, regardless of the causal relationship to study treatment

-In case of Grade 1 (CTC-AE classification) maculo-papular rash or desquamation, study treatment will be maintained and patient will be treated according to clinical practice.

-In case of Grade 2 (CTC-AE classification) maculo-papular rash or desquamation, study treatment will be interrupted, and patient will be treated according to clinical practice. After return to baseline or Grade ≤ 1, study treatment will be resumed at the same dose level as before interruption. In case of reoccurrence of a Grade 2 maculo-papular rash or desquamation, study treatment must be interrupted and symptomatic treatment should be initiated. After return to baseline or Grade ≤ 1, study treatment will be resumed according to local investigator’s judgement.

-In case of Grade 3 skin toxicity, study treatment should be interrupted and a dermatologist should be consulted, assess the risk and define the symptomatic treatment for the patient.

Via Giardini, 1355
Baggiovara
41126 Modena
tel.: 059396111
fax.: 0593962409

The dermatologist will give his/her opinion on whether patient could resume study treatment depending on skin lesions and patient safety.

-In case of Grade 4 skin toxicity, a dermatologist must be consulted and study treatment must be definitely discontinued.

There have been reports of impaired or delayed wound healing in patients receiving Rapamycin, including wound dehiscence; in these cases treatment should be discontinued until normalization of wound healing.

10. Carcinogenicity

Risk of Lymphoma/ lymphoproliferative disease/ Skin Carcinoma

Immunosuppression may increase the risk of skin or haematopoietic neoplasms. Patients should be carefully examined at each evaluation (until study end) and sun exposure should be limited. A relatively long period follow up has been chosen to evaluate this risk. In case of suspect of neoplasm, the study treatment will be interrupted, the patient should be referred to a specialist who will decide the complementary tests to confirm positive or suspicious results.

11. Increased Susceptibility to Infection

Oversuppression of the immune system can also increase susceptibility to infection, including opportunistic infections such as tuberculosis, fatal infections, and sepsis.

Immunosuppressed patients are at increased risk for opportunistic infections, including activation of latent viral infections. These include BK virus-associated nephropathy, which has been observed in patients receiving immunosuppressants. This infection may be associated with serious outcomes, including deteriorating renal function. Patient monitoring may help detect patients at risk for BK virus-associated nephropathy. Treatment discontinuation should be considered for patients who develop evidence of BK virus-associated nephropathy.

Infections from CMV, EBV, JCV have also been reported and in this cases treatment should be discontinued until recovery, and in case of duration >2 weeks, definitely stopped.

12. Risk management plan for adverse events not described above and suspected to be related to study treatment

Please note that, the previous rules apply regardless of the relationship with study medication, while this rule applies only for adverse events suspected to be related to study treatment

- At the first occurrence of moderate adverse event (grade 2): study treatment will be interrupted until adverse event has returned to baseline value or mild intensity, then resumed at the same dose level according to local investigator judgement.

- If the same moderate adverse event re-occurs, study treatment will be interrupted.

- In case of severe adverse event (grade 3), study treatment will be interrupted until adverse event has returned to baseline level or mild intensity, then resumed with a dose reduction (one step).

- In case of life-threatening or disabling adverse event (grade 4), study treatment must be definitely discontinued

9. EVALUATION CRITERIA

9.1. PRIMARY CRITERIA

Change from baseline to week 18 in Tregs number in ALS patients treated with Rapamycin compared to the control arm.

9.2. SECONDARY CRITERIA

9.2.1. Safety Variables

The Safety will be assessed on the following variables:
Occurrence of adverse events (AEs) and per-treatment arising changes in physical examination, vital signs (blood pressure, pulse rate and body temperature), body weight, and clinical laboratory tests (biochemistry, haematology). MEDRa dictionary is going to be used for reporting.

9.2.1.1. Adverse Events

An adverse event (AE) is defined as any modification of the clinical status of the patient, i.e. any emergence of a disease, sign or symptom, or modification of sign, symptom or concomitant disease, regardless of its relationship to study treatment.
All adverse events will be actively collected at each visit, from spontaneous declarations of the patient as well as from oral inquiry and clinical examination.
All AE should be noted in the case report form on the ad hoc "Adverse events" form. Theinvestigator should specify its nature, date of onset, duration, outcome, actions taken, date of disappearance or stabilization. The investigator should evaluate the event in terms of severity, relationship to study treatment and seriousness.
Definitions for adverse event (AE) and serious adverse event (SAE), as well as procedures to follow in case of SAE are presented in Appendix 17.1.

9.2.1.2. Concomitant Treatments

All concomitant medications and/or therapies should be documented in the patient file and reported in the electronic Case Report Form (eCRF).

9.2.1.3. Laboratory Tests

The following parameters will be assessed:
Haematology
Haematology includes count of red blood cells, hemoglobin, hematocrit, total white blood cells count, platelet count, and a differential count including neutrophils, lymphocytes, monocytes, eosinophils, and basophils, and will be performed according to the Visit Schedules (see study flowcharts).
Biochemistry

Biochemistry includes urea, creatinine, albumin, protein electrophoresis, AST (SGOT), ALT (SGPT), gamma GT, LDH, cholesterol, triglycerides, sodium, potassium, glucose, will be performed according to the Visit Schedules (see study flowcharts).

Screening for infectious diseases

Screening for infectious diseases includes HIV, Hepatitis B, Hepatitis C and tuberculosis (intra-dermal reaction test or Interferon Gamma Release Assay) screening will be done locally.

9.2.1.4. Physical Examination/Vital Signs

A physical examination including vital signs (systolic and diastolic arterial pressure, heart rate) will be performed according to the Visit Schedule. Information about the physical examination and vital signs must be present in the source documentation at the study site. Significant findings present prior to the start of study drug must be included in the Medical History and Current Conditions CRF page. Significant findings made after the start of study drug which meet the definition of an adverse event must be recorded on the Adverse Event CRF page.

9.2.1.5. Body Weight

Measurements of body weight will be performed according to the Visit Schedule. Body weight measurements performed during visits physical examination, will be captured on CRFs, which will calculate BMI and BSA.

9.2.1.6. Other Safety Parameters

Electrocardiogram and Chest X ray

An electrocardiogram and chest X-ray (only Posterior-Anterior view) will be performed at screening. At treatment end (week 18) these examex can be repeated based on clinical opinion of the caring neurologist.

Forced Vital capacity

The forced vital capacity (FVC) (percent of predicted normal) is the vital capacity (VC) measured when the patient is exhaling with maximal speed and effort. It will be determined, using the slow VC method. The VC can be measured using conventional spirometers that have had a calibration check prior to testing. A printout from the spirometer of all VC trials will be retained. Three VC trials are required for each testing session, and the highest VC recorded is utilized for eligibility. Vital capacity results at baseline, even those less than 70%, will not preclude enrolment.

Pregnancy test

For female of child bearing potential patients, pregnancy test will be performed by collecting serum at screening, baseline and final visit.

9.2.2. BIOLOGICAL ACTIVITY ASSESSMENT

The following secondary variables will be assessed:

- Rapamycin levels in CSF at week 18 (high-performance liquid chromatography (HPLC) with mass spectrometry (MS) (LC-MS/MS).
- Change from baseline to each time point (week 8, 18, 30, and 54) of the phosphorylation of the S6 ribosomal protein (S6RP) comparing Rapamycin arms and placebo arm.

- Change from baseline to each time point (week 8, 18, 30, and 54) of the activation and homing capabilities of different T, B, NK cell subpopulations comparing Rapamycin arms and placebo arm.
- Changes from baseline to each time point (week 8, 18, 30, and 54) in different biomarkers (including peripheral and CSF biomarkers i.e. creatinine and albumin, CK, vitamin D, plasma/CSF neurofilament heavy/light chain protein) comparing Rapamycin arms and placebo arm.
- Changes from baseline to each time point (week 8, 18, 30, and 54) in inflammatory status (molecular analysis of the inflammasome system) comparing Rapamycin arms and placebo arm.

-

9.2.3. CLINICAL ASSESSMENT

The following secondary variables will be assessed:

-Change from baseline to each time point (week 4, 8, 12, 18, 30, 42 and 54) of ALSFRS-R
-Survival defined as the time from randomisation to the date of documented death or tracheotomy
-Survival rate at week 18, 36 and week 54
-Change of Forced Vital Capacity (FVC) from baseline to each time point (week 4, 8, 12, 18, 30, 42 and 54)

9.2.4. QUALITY OF LIFE

Quality of life will be assessed on the change in absolute value and percentage of following variables:

-Absolute and relative change from baseline in ALSAQ-40 (Amyotrophic Lateral Sclerosis Specific Assessment Questionnnaire at week 4, 8, 12, 18, 30, 42 and 54)

10. ASSESSMENT SCHEDULE

10.1. SCREENING VISIT

Prior to any study activities, the patient will be asked to read and sign an informed consent form that has been approved by the Independent Ethics Committees and which complies with regulatory requirements. Patients will be given adequate time to consider any information concerning the study

given to them by the investigator. As part of the informed consent procedure, patients will be given the opportunity to ask the investigator any question regarding potential risks and benefits of a participation in the study.

The following assessments and investigations will be conducted during this visit:

-Written informed consent
-Give information form for general practitioner
-Document the relevant past medical history, any planned surgery and current medical conditions not related to the diagnosis of ALS
-ALS history and diagnosis (date of diagnosis, signs and symptoms…)
-Relevant medical history and procedure history (start and end dates…)
-ALS medication history
-Concomitant medications and/or non-drug therapies, including the reason for administration
-Check of the patient's current practice for effective contraception
-Physical examination including vital signs, weight, height (BMI/BSA)
-Neurological examination
-Perform MRC, ALSFRS-R and FVC measurement
-Urine will be collected for urinalysis
-Blood sample will be collected for haematology and biochemistry
-Serum pregnancy test in females of child-bearing potential
-Screening for infectious diseases: HIV, Hepatitis B, Hepatitis C, TB test
-12-lead ECG; in case a 12-lead ECG has been performed within 4 weeks prior to screening, it might be used as baseline ECG
-Chest X-Ray (only Posterior-Anterior view); in case a chest X-Ray has been performed within 1 month prior to screening, it might be used as baseline X-Ray
-Check of inclusion/exclusion criteria
-Recording of symptoms present at screening
-Appointment for the next visit

10.2. BASELINE VISIT

The baseline visit can occur from 1 day and up to 2 weeks after the screening visit when all results from the screening evaluation are available, and study treatment is available at the study site.

The following measures should be conducted before the randomisation:

-Check of the patient's current practice for effective contraception
-Check of inclusion/exclusion criteria
-Physical examination including vital signs, weight, height (BMI/BSA)
-Neurological examination
-Perform MRC, ALSFRS-R and FVC measurement
-ALSAQ – 40 (Amyotrophic Lateral Sclerosis Assessment Questionnaire)
-Urine will be collected for urinalysis (analysis will not be repeated if Visit 2 occurs less than 7 days after Visit 1)

-Blood sample will be collected for haematology and blood chemistry (analysis will not be repeated if Visit 2 occurs less than 7 days after Visit 1)
-Blood sample will be collected in the afternoon for biological activity assessment (to be sent to the Laboratory of Immunology of Modena University within 9 am of the next day)
-Perform lumbar puncture and store CSF
-Recording of adverse events
-Recording of menstrual cycles (non menopausal female patients)
-Query for medical procedures, hospitalizations, changes in concomitant medications and therapies
-Randomisation and treatment allocation

The following measures should be conducted after randomisation:
-Dispensing of study treatment and study treatment administration.
-Recording of dispensed treatment number(s) on CRF
-Instructions to the patient:
-Daily dose of treatment at approximately the same on time each day, before breakfast, except at subsequent visits when Rapamycin dosage will be performed, because morning dose of treatment will be taken after blood sample for Rapamycin dosage.
-Store treatment at room temperature, out of children's reach
-Both used and unused study treatment bottles must be returned at the next visit for treatment accountability
-Ask the patient to come back next week for Rapamycin dosage.

10.3.1 VISIT WEEK 1 (for the first 10 patients enrolled)

The following assessments and investigations will be conducted:
-Check with the patient any signs of underlying infection and the absence of any other adverse event, and compliance with the drug assumption.
-Check of the patient's current practice for effective contraception
-Physical examination including vital signs, weight, height (BMI/BSA), neurological examination, ALSFRS-R, MRC
-Blood sample will be collected for haematology and blood chemistry (flow charts 1a and 2a)
-Recording of adverse events
-Recording of menstrual cycles (non menopausal female patients)
-Query for medical procedures, hospitalizations, changes in concomitant medications and therapies
-Blood sample will be collected for Rapamycin dosage (the patient will take the tablets after blood sample, at fast; then he will do breakfast). This analysis will be performed locally, with HPLC method.
-One person of the centre, who will not visit the patients or communicate with the caring neurologist, will call the coordinator centre (Modena), to communicate Rapamycin dosage. The coordinator centre could confirm the dosage or reduce it to avoid toxicity. Shamreduction will be also performed to maintain study blindness.
-Dose adjustment if necessary

-Appointment for the next visit

10.3.2 VISIT WEEK 1 (for the following 53 patients enrolled)

The following assessments and investigations will be conducted:

-Blood sample will be collected for Rapamycin dosage (the patient will take the tablets after blood sample, at fast; then he will do breakfast). This analysis will be performed locally, with HPLC method.
-One person of the centre, who will not visit the patients or communicate with the caring neurologist, will call the coordinator centre (Modena), to communicate Rapamycin dosage. The coordinator centre could confirm the dosage or reduce it to avoid toxicity. Shamreduction will be also performed to maintain study blindness.
-Call by the center to the patient to check with the patient any signs of underlying infection and the absence of any other adverse event, and compliance with the drug assumption.
-Dose adjustment if necessary
-Appointment for the next visit

10.4.1 VISITS WEEK 3, WEEK 10, WEEK 14, WEEK 16, TREATMENTPERIOD (for the first 10 patients enrolled)

The following assessments and investigations will be done:
-Check with the patient any signs of underlying infection and the absence of any other adverse event, and compliance with the drug assumption.
-Check of the patient's current practice for effective contraception
-Physical examination including vital signs, weight, height (BMI/BSA), neurological examination, ALSFRS-R, MRC
-Blood sample will be collected for haematology and blood chemistry (flow charts 1a and 2a)
-Recording of adverse events
-Recording of menstrual cycles (non menopausal female patients)
-Query for medical procedures, hospitalizations, changes in concomitant medications and therapies
-Appointment for the next visit

10.4.2 VISITS WEEK 5, WEEK 7, WEEK 9, WEEK 11, WEEK 13, WEEK 15,WEEK 17, TREATMENT PERIOD (for the first 10 patients enrolled)

The following assessments and investigations will be conducted:

-Weekly call by the center to the patient. The purpose of the call is to check with the patient any adverse reaction and compliance with the drug.

10.4.3 VISITS WEEK 3, WEEK 5, WEEK 7, WEEK 9, WEEK 11, WEEK 13,WEEK 15, WEEK 17, TREATMENT PERIOD (for the following 53 patientsenrolled)

The following assessments and investigations will be conducted:

-Weekly call by the center to the patient up to week 17. The purpose of the call is to check with the patient any adverse reaction and compliance with the drug.

10.5. VISITS W2, W6 TREATMENT PERIOD

The following assessments and investigations will be conducted at each visit:

-Check of the patient's current practice for effective contraception
-Physical examination including vital signs, weight, height (BMI/BSA)
-Blood sample will be collected for haematology and blood chemistry
-Recording of adverse events
-Recording of menstrual cycles (non menopausal female patients)
-Query for medical procedures, hospitalizations, changes in concomitant medications and therapies
-Blood sample will be collected for Rapamycin dosage (the patient will take the tablets after blood sample, at fast; then he will do breakfast). This analysis will be performed locally, with HPLC method.
-One person of the centre, who will not visit the patients or communicate with the caring neurologist, will call the coordinator centre (Modena), to communicate Rapamycin dosage. The coordinator centre could confirm the dosage or reduce it to avoid toxicity. Shamreduction will be also performed to maintain study blindness.
-Dose adjustment if necessary
-Appointment for the next visit

10.6. VISITS W4, W12 TREATMENT PERIOD

The following assessments and investigations will be conducted at each visit:

-Check of the patient's current practice for effective contraception
-Recording of adverse events
-Recording of menstrual cycles (non menopausal female patients)
-Query for medical procedures, hospitalizations, changes in concomitant medications and therapies
-Physical examination including vital signs, weight, height (BMI/BSA)
-Neurological examination
-Perform MRC, ALSFRS-R and FVC measurement
-ALSAQ – 40 (Amyotrophic Lateral Sclerosis Assessment Questionnaire)
-Blood sample will be collected for haematology and blood chemistry

-Blood sample will be collected for Rapamycin dosage (the patient will take the tablets after blood sample, at fast; then he will do breakfast). This analysis will be performed locally, with HPLC method.
-One person of the centre, who will not visit the patients or communicate with the caring neurologist, will call the coordinator centre (Modena), to communicate Rapamycin dosage. The coordinator centre could confirm the dosage or reduce it to avoid toxicity. Shamreduction will be also performed to maintain study blindness.
-Dose adjustment if necessary
-Dispensing of study treatment and study treatment administration.
-Recording of dispensed treatment number(s) on CRF
-Appointment for the next visit

10.7. VISITS W8 TREATMENT PERIOD

The following assessments and investigations will be conducted at each visit:
-Check of the patient's current practice for effective contraception
-Recording of adverse events
-Recording of menstrual cycles (non menopausal female patients)
-Query for medical procedures, hospitalizations, changes in concomitant medications and therapies
-Physical examination including vital signs, weight, height (BMI/BSA)
-Neurological examination
-Perform MRC, ALSFRS-R and FVC measurement
-ALSAQ – 40 (Amyotrophic Lateral Sclerosis Assessment Questionnaire)
-Blood sample will be collected for haematology and blood chemistry
-Blood sample will be collected for Rapamycin dosage (the patient will take the tablets after blood sample, at fast; then he will do breakfast). This analysis will be performed locally, with HPLC method.
-One person of the centre, who will not visit the patients or communicate with the caring neurologist, will call the coordinator centre (Modena), to communicate Rapamycin dosage. The coordinator centre could confirm the dosage or reduce it to avoid toxicity. Shamreduction will be also performed to maintain study blindness.
-Dose adjustment if necessary
-Blood sample will be collected in the afternoon for biological activity assessment (to be sent to the Laboratory of Immunology of Modena University within 9 am of the next day)
-Dispensing of study treatment and study treatment administration.
-Recording of dispensed treatment number(s) on CRF
-Appointment for the next visit

10.8. END OF TREATMENT VISIT , WEEK 18

The following assessments and investigations will be conducted:
-Check of the patient's current practice for effective contraception
-Recording of adverse events
-Recording of menstrual cycles (non menopausal female patients)

-Query for medical procedures, hospitalizations, changes in concomitant medications and therapies
-Physical examination including vital signs, weight, height (BMI/BSA)
-Neurological examination
-Perform MRC, ALSFRS-R and FVC measurement
-ALSAQ – 40 (Amyotrophic Lateral Sclerosis Assessment Questionnaire)
-Urine will be collected for urinalysis
-Blood sample will be collected for haematology and blood chemistry
-Blood sample will be collected in the afternoon for biological activity assessment (to be sent to the Laboratory of Immunology of Modena University within 9 am of the next day)
-Blood sample will be collected for Rapamycin dosage (the patient will take the tablets after blood sample, at fast; then he will do breakfast). This analysis will be performed locally, with HPLC method.
-One person of the centre, who will not visit the patients or communicate with the caring neurologist, will call the coordinator centre (Modena), to communicate Rapamycin dosage. No treatment adjustment will be suggested (because of the end of treatment).
-12-lead ECG (if necessary, on medical opinion)
-Chest X ray (only Posterior-Anterior view) (if necessary, on medical opinion)
-Lumbar puncture: the patient will arrive at the centre and blood sample will be drawn. Patient will take the last assumption of Rapamycin and the n will go to have breakfast. He will also undergo other examinations as scheduled in this visit, and after 3 hours from Rapamycin assumption he will undergo lumbar puncture.
-Return of study treatment. Tablet accountability. Recording of the start and stop date of treatment taken between visits, of number of dose units returned as well as explanations of non-compliance in the CRF. Any deviation from the treatment administration schedule and discrepancies identified must be recorded in source documents and on the CRF
-Appointment for the next visit

10.9. FOLLOW UP VISITS, WEEK 24

The following assessments and investigations will be conducted at each visit:
-Check of the patient's current practice for effective contraception
-Recording of adverse events
-Recording of menstrual cycles (non menopausal female patients)
-Query for medical procedures, hospitalizations, changes in concomitant medications and therapies
-Physical examination including vital signs, weight, height (BMI/BSA)
-Neurological examination
-Perform MRC, ALSFRS-R measurement
-Appointment for the next visit

10.10. FOLLOW UP VISITS, WEEK 30, WEEK 42

The following assessments and investigations will be conducted at each visit:

-Check of the patient's current practice for effective contraception
-Recording of adverse events
-Recording of menstrual cycles (non menopausal female patients)
-Query for medical procedures, hospitalizations, changes in concomitant medications and therapies
-Physical examination including vital signs, weight, height (BMI/BSA)
-Neurological examination
-Perform MRC, ALSFRS-R and FVC measurement
-ALSAQ – 40 (Amyotrophic Lateral Sclerosis Assessment Questionnaire)
-Blood sample will be collected for haematology and blood chemistry
-Blood sample will be collected in the afternoon for biological activity assessment (to be sent to the Laboratory of Immunology of Modena University within 9 am of the next day) only at week 30.
-Appointment for the next visit

10.11 END OF STUDY VISIT (WEEK 54)

The following assessments and investigations will be conducted:
-Check of the patient's current practice for effective contraception
-Recording of adverse events
-Recording of menstrual cycles (non menopausal female patients)
-Query for medical procedures, hospitalizations, changes in concomitant medications and therapies
-Physical examination including vital signs, weight, height (BMI/BSA)
-Neurological examination
-Perform MRC, ALSFRS-R and FVC measurement
-ALSAQ – 40 (Amyotrophic Lateral Sclerosis Assessment Questionnaire)
-Blood sample will be collected for haematology and blood chemistry
-Blood sample will be collected in the afternoon for biological activity assessment (to be sent to the Laboratory of Immunology of Modena University within 9 am of the next day)

10.12. WITHDRAWAL OF PATIENTS

All interruptions or any changes in study treatment administration must be documented in patient file and reported in the Trial Medication Compliance part of the Case Report Form (CRF).
Patient may withdraw from the study for any of the following reasons:
-Withdrawal of consent
-Adverse or intercurrent event considered intolerable by the patient or incompatible with continuation of the study according to the investigator
-Protocol violation (e.g., noncompliance with treatment administration, prohibited treatment needed)
-Worsening of the patient's disease status requiring a change in treatment, as determined by the

investigator

-Suspected pregnancy or positive pregnancy test result. Any suspected pregnancy must be immediately confirmed by a serum pregnancy test

If the patient discontinues study treatment during the study, the reason must be given on the study completion form as one of the following:

-Adverse Event
-Patient's request / Unwillingness to continue
-Lost to follow-up
-Documented progressive disease
-Protocol Deviation
-Death
-Other (to be specified by the investigator)

The investigator should record the main reason for discontinuation of treatment in the eCRF and single out the primary reason if more than one applies.
If a patient discontinues the study for an adverse event, s/he must be followed up weekly for 4 weeks, or until resolution or stabilization of the event, whichever comes first.
In case of premature withdrawal, the patient will undergo all examinations scheduled for the last trial visit.
A final visit will be performed within 2 weeks of last study treatment intake on patients who are withdrawn from the study prematurely, at any time after receiving the first dose of study medication.

11. STATISTICAL METHODS

11.1. SAMPLE SIZE JUSTIFICATION

The sample size has been estimated considering as the primary outcome measure the proportion of positive response (Tregs number increase) at treatment end (18 weeks) in pts treated with Rapamycin vs placebo.

ALS pts have a slight reduction of Treg% (mean±SD:2.1±0.7) with respect to healthy controls (2.6±0.6)(Treg% calculated on total lymphocytes; normal values of total lymphocytes: 1000-4500/mmc; normal values of total Treg: 71.5±17/mmc)(Mantovani,2009).
Slowly progressive ALS pts have a number of Tregs that is equal to healthy controls, whereas fast progressors have 31% fewer Tregs than slowly progressing pts, and Treg % is inversely correlated with the rate of disease progression (Beers,2011). These data indicates that ALS pts have 60±17 Treg/mmc (fast progressors: 49.3Treg/mmc; slow progressors: 71.5Treg/mmc). As a result, a "positive response" can be considered an increase of the proportion of Tregs by at least 30%. The null hypothesis is that Rapamycin does not increase significantly the proportion of positive responses in treated pts at 18 weeks compared to their baseline stage and to placebo group. The alternative hypothesis is that Rapamycin determines a proportion of positive responses in at least 50% of treated patients compared to a maximum 5% of patients in the placebo group. The study hasbeen designed to reject the null hypothesis with an alpha error of 0.025 (in order to take into account a multiple comparison with a control arm) and a power of 0.80. For this purpose, a sample of 54 pts randomized in 3 treatment arms would be needed. Considering an average drop out of15% then a recruitment of 63 patients will be necessary.

11.2 STATISTICAL ANALYSIS

Data management and statistical plan: Partner 2 will be in charge for this work (details in WP4). An independent Data and Safety Monitoring Committee will be established with several meetings aimed at preserving safety (see safety evaluation box).

Statistical methods:

Separate analyses will be performed in:
1. All randomized subjects receiving at least 1 dose of study medication (**Intention-to-treat population**);
2. All randomized subjects excluding protocol deviations (**Per protocol, PP population**).
Descriptive statistics will be performed comparing the 2 groups of Rapamycin treatment and placebo. Continuous variables will be described using mean and standard deviation or median and interquartile range; categorical variables will be described as counts and percentages.

Via Giardini, 1355
Baggiovara
41126 Modena
tel.: 059396111
fax.: 0593962409

Safety analysis will be performed in all subjects receiving at least one dose of the experimental drug. All AEs, SAEs and AEs leading to treatment discontinuation will be recorded according to ICH Guidelines, listed and compared in the treatment arms at any follow-up visit and at the end of the study.

Differences in tracheostomy-free survival (Kaplan-Meier method) between the treated groups and placebo group will be compared using the log-rank test. Cox's proportional hazard model would be used to adjust for any possible unbalanced prognostic factors. Statistical significance will be set at 0.05 level for a two-tailed test. Missing data will be handled using the last observation carried forward.

Rapamycin activity analysis:

Immune response to Rapamycin (R)) will be analyzed as the difference in positive response to Rapamycin (mean Tregs increase >30%) between the placebo group and the Rapamycin groups. This will be calculated with the use of Treg data obtained at baseline and at week 18.

We will compare the mean values of S6RP phosphorylation, of different T, B, NK cell subpopulations, of biomarkers, inflammasome, cytokines, comparing baseline and treatment end(18 weeks) between Rapamycin and placebo arm. Mean differences in plasma concentrations from baseline to week 18 in the 2 treatment arms will be calculated and compared using t-test or Wilcoxon-Mann-Whitney test.

The mean change over time for the same variables as above will be assessed using repeated measures ANOVA, with treatment as between-subjects factor and time as within-subjects factor. Different models will be used, each with a different biomarker of activity as the dependent variable.Models will be adjusted for any unbalanced distribution of the main prognostic factors (e.g. age) between the two treatment arms.

11.3 STOPPING RULES FOR SAFETY REASONS

We will consider the following list of toxicities as acceptable:

Peripheral edema, hypertriglyceridemia, hypercholesterolemia, constipation, arthralgia, thrombocytopenia (not severe), anemia (not severe), leukopenia (not severe), rash, hypertension, increased creatinine, abdominal pain, diarrhoea, headache, fever, urinary tract infection, nausea, lymphocele, tachycardia, stomatitis, abnormal healing, increased lactic dehydrogenase,hypokalemia (not severe), diabetes mellitus, epistaxis, ovarian cysts and menstrual disorders(amenorrhea and menorrhagia) .

The study will be stopped by the DSMB in case of more than 30% of patients experience one of the following side effects: pneumonia, sepsis, venous thromboembolism, thrombotic thrombocytopenic purpura / hemolytic uremic syndrome, severe leucopenia or anemia or thrombocytopenia, bone necrosis, melanoma, skin cancer. In other words the trial will be stopped if any of the above mentioned SAEs occurs in at least 6 patients

As for mild and moderate adverse events, the DSMB will be notified if 50% of all subjects report a given mild or moderate severity adverse effect.

12. DATA RECORDING AND STUDY MONITORING

All data will be recorded by an electronic CRF.
The study will be monitored by a certified contract research organization (CRO).
A medical monitor will be in charge of safety data downloads and review on monthly basis (adverse events), laboratory data downloads, including Rapamycin levels on fortnightly basis.
DSMB meetings will be scheduled when the first 10 patients would have been treated for 3 months, and when 50% of pts would have completed week 8, 18, 36, and 54

13. DATA MANAGEMENT

Data from the CRFs are entered into the study database using EDC (Electronic Data Capture).
Subsequently, the information entered into the database is systematically checked:
-On line with automatic checks
-Off line by Data Management staff (Statistics Unit based at University of Modena)
using error messages from validation programs or database listings.
Error message will be entered on Data Clarification Forms and entered into the data-base by the investigator using EDC. Investigator will sign the final eCRF with electronic signature. Thisprocess follows until the lock of the data-base. Quality control audits of all key safety and efficacy data in the database will be made by the data-manager before locking the data-base.
Concomitant medications entered into the database will be coded using the WHO Drug Reference List which employs the Anatomical Therapeutic Chemical classification system. Coexistent diseases and adverse events will be coded using MedDRA.
When the database has been declared to be complete and accurate, the database will be locked.

14. ETHICS

14.1 ETHICAL CONSIDERATIONS

The study will be carried out in accordance with the Declaration of Helsinki (Appendix 17.2), as amended by the 64th WMA General Assembly, Fortaleza, Brazil, October 2013.

14.2 SUBJECT INFORMATION AND INFORMED CONSENT

All subjects must sign and personally date an approved Informed Consent Form after receiving detailed written and verbal information about the reason, the nature, the required procedures, the

intended duration and the possible risks and benefits and any discomfort associated with the study. He/She should be informed that the subject's participation in the study is voluntary and that he/she may refuse to participate or withdraw from the trial, at any time, without penalty or loss of benefits to which the subject is otherwise entitled. The language used in the oral and written information about the trial, including the written Informed Consent Form, should be as non technical as practicaland should be understandable to the subject or the subject's legally acceptable representative andthe impartial witness, where applicable. The subject must be given ample time to read and to understand the Patient Information Sheet and opportunity to inquire and ask any clarification about the trial before signing the Informed Consent Form.
Prior to a subject's participation in the trial, the written Informed Consent Form should be signed and personally dated by the subject or by the subject's legally acceptable representative, and by the physician who conducted the informed consent discussion. When applicable the Investigator may conduct the informed consent discussion in presence of an impartial witness, who should sign and personally date the Informed Consent Form.
No study procedure can be performed before the written informed consent has been provided.
The informed consent procedure must be done according to the guidelines provided in the Declaration of Helsinki and the ICH E6 Guideline for Good Clinical Practice.
The subject must be made aware and agree that personal information may be scrutinized during audit by competent authorities and properly authorized persons. However, personal information willbe treated as strictly confidential and will not be publicly available.
By signing the Investigator Statement (Appendix) the Investigator assures the Sponsor that Informed Consent will be obtained.
Both the Patient Information Sheet and the Informed Consent Form must be approved by the Ethics Committee with the study protocol.

14.3 PATIENT WITHDRAWALS AND DROPOUTS

Any study subject may be withdrawn from the study at any time either at the discretion of the investigator or at the request of the study subject.
The reason for doing so should be clearly documented in the CRF.
However, the study subject is not under any obligation to provide a reason for withdrawal.

14.4 ETHIC COMMITTEE APPROVAL

The protocol, Subject Information Sheet, Informed Consent Form and any advertisement for the recruitment of subjects must be reviewed and approved by an appropriately constituted Ethic Committee, as required in chapter 3 of the ICH E6 Guideline.
Written EC approval must be obtained by the Sponsor prior to shipment of study agent or subject enrollment.
The Investigator is committed in accordance with local requirements to inform the IRB/IEC of any emergent problem, serious adverse events, and/or protocol amendments.

14.5 INDEPENDENT DATA MONITORING COMMITTEE

An Independent Data and Safety Monitoring Board (DSMB) without direct involvement in the conduct of the study, will be set up and notified to the Ethical Committee of the coordinating centre specifically to monitor safety data throughout the duration of the study to determine if continuation is appropriate both scientifically and ethically. All adverse events occurring during the trial will be forwarded to the Committee.

15. ADMINISTRATIVE CONSIDERATIONS

Regulatory Requirements–Sponsor/Investigator Obligations

This study will be conducted in accordance with the Declaration of Helsinki and the ICH E6 Guideline (Good Clinical Practice). To ensure compliance the Investigator agrees, by written consent to this protocol, to fully cooperate with compliance checks by allowing access to all documentation, including patient hospital files (the source documents), by authorised individuals.

Protocol Amendments

Protocol directions must be strictly adhered to. In case of need a written Protocol Amendment will be prepared. The Amendment will be approved and signed by the involved parties (Investigator and Sponsor) according to the same procedure followed for the study protocol.
The Amendment will not be implemented before the approval of the Ethics Committee.

Curriculum Vitae

The Investigator and any co-Investigator(s) must provide the coordinating centre with currentcopies of their own curriculum vitae.

Investigator's Statement

This document, signed and dated by the Principal Investigator, describes the Investigator's obligations.

Role of participating centres

Each centre is expected:
1.to randomize at least 7 patients fulfilling including and excluding criteria in a period of 12 months and to administer the treatment for 18 weeks;
2.to provide one principal investigator and one neurologist to evaluate including and excluding criteria, administer treatment, and assess primary and secondary outcome.
3. to provide a person who will send Rapamycin dosage to the coordinating centre and who will not see patients
3.to formally adhere to the practice parameters of the European Federation of Neurological Societes concerning the standardization of the management of the patient in terms of ventilatory support and nutrition.

Monitoring procedures

Study monitoring

The study monitor indicated by the coordinating centre will be in contact with the investigator and will conduct a visit to the Centre to discuss and/or collect data. The Monitor will conduct a visit before the start of the study to discuss the protocol and obligations of the investigator and sponsor. The investigator is required to allow the Monitor to conduct the site visit, the study-end visit and thesite closure visit.

The purposes of these visits are:

- Make sure that the written informed consent was obtained for each subject before participation in the study
- Assess the progress of the study.
- Ensure compliance with the study protocol.
- Check whether all adverse events were correct relationship.
- Make sure that the investigator retains the essential documents of the study.
- To discuss any problem that arises.
- Review the CRF for readability, accuracy and completeness of the compilation.
- Validating Data entered in the CRF versus the source files.
- Check the correctness of storage, distribution and recovery of the drug experimentation.

The investigator is required to make available for inspection the documents source. Information contained therein will be treated as confidential.

The Monitor will conduct a final inspection to close the center to the end of the study.

Electronic Case Report Form (eCRF)

All information about the study will be collected in the eCRF.

If requested, copies of the eCRF are to be made available to the appropriate regulatory agencies.

Auditing

The Investigator will make all pertinent records available including source documentation for inspection by regulatory authorities. This information will be considered as confidential.

Archiving of Records

Copies of the protocol, subject identification codes, electronic Case Report Form, source data, Informed Consent Form and other documents pertaining to the study conduction and support the data collected from each subject must be kept for the maximum period of time as required by the study centre. In compliance with the ICH/GCP guidelines, this time period must be at least two years after the last approval of the marketing application of the study agent in an ICH region and until there is no pending or contemplated marketing applications in an ICH region or at least two years have elapsed since the formal discontinuation of clinical development of the study agent.

No study document should be destroyed.

Originals of all documentation and copies of outgoing correspondence concerning the study will be stored and retained by the Sponsor in a safe area in the Trial Master File for the lifetime of the product. In particular, the final report must be retained by the Sponsor, or the subsequent owner, for five years beyond the lifetime of the study agent.

Role of funding

This is an academic, independent clinical research project funded by the Italian Agency for the Research on Amytrophic Lateral Sclerosis (AriSLA). The pharma company (Pfizer) that will give RAPAMYCIN for free will not be involved at any level in the study.
Participating units and patients will not be paid.

Use and Publication of Study Results

The results of the study may be presented during scientific symposia or published in scientific journals only after review and written approval by the involved parties in full respect of the privacy of the participating subjects. None of the investigators at the participating centres can make use of any information or data before, during and after the study without the written approval from the principal investigator (Dr. Jessica Mandrioli).

Insurance Policy

An insurance company will provide insurance coverage for damages emerging from the trial and involving test subjects treated with the test compound. The principal Investigator will be supplied with all data concerning the insurance company and policy number.

16. REFERENCES

1. Barmada SJ, Serio A, Arjun A, et al. Autophagy induction enhances TDP43 turnover and survival in neuronal ALS models. Nat Chem Biol. 2014 Aug;10(8):677-85. doi: 10.1038/nchembio.1563.
2. Beers DR, Henkel JS, Zhao W, et al. Endogenous regulatory T lymphocytes ameliorate amyotrophic lateral sclerosis in mice and correlate with disease progression in patients with amyotrophic lateral sclerosis. Brain. 2011 May;134(Pt 5):1293-314. doi: 10.1093/brain/awr074.
3. Buchan JR, Kolaitis RM, Taylor JP, Parker R. Eukaryotic stress granules are cleared by autophagy and Cdc48/VCP function. Cell. 2013;153:1461-74. doi: 10.1016/j.cell.2013.05.037.
4. Caccamo A, Majumder S, Deng JJ, Bai Y, Thornton FB, Oddo S. Rapamycin rescues TDP-43 mislocalization and the associated low molecular mass neurofilament instability. J Biol Chem. 2009 Oct 2;284(40):27416-24. doi: 10.1074/jbc.M109.031278.
5. Chiò A, Hammond ER, Mora G, Bonito V, Filippini G. Development and evaluation of a clinical staging system for amyotrophic lateral sclerosis. J Neurol Neurosurg Psychiatry. 2015 Jan;86(1):38-44. doi: 10.1136/jnnp-2013-306589.
6. Cirulli ET, Lasseigne BN, Petrovski S, et al. Exome sequencing in amyotrophic lateral sclerosis identifies risk genes and pathways. Science 2015;347:1436-41. doi: 10.1126/science.aaa3650.
7. Cloughesy TF1, Yoshimoto K, Nghiemphu P et al. Antitumor activity of rapamycin in a Phase I trial for patients with recurrent PTEN-deficient glioblastoma. PLoS Med. 2008 Jan 22;5(1):e8. doi: 10.1371/journal.pmed.0050008.
8. Deivasigamani S, Verma HK, Ueda R, et al. A genetic screen identifies Tor as an interactor of VAPB in a Drosophila model of amyotrophic lateral sclerosis. Biol Open 2014;3:1127-38. doi: 10.1242/bio.201410066.).
9. Harrison DE, Strong R, Sharp ZD, et al. Rapamycin fed late in life extends lifespan in genetically heterogeneous mice. Nature. 2009;460:392-5. doi: 10.1038/nature08221.
10. Kimura F, Fujimura C, Ishida S, et al. Progression rate of ALSFRS-R at time of diagnosis predicts survival time in ALS. Neurology 2006;66:265-7.
11. Komatsu M, Waguri S, Chiba T, et al. Loss of autophagy in the central nervous system causes neurodegeneration in mice. Nature 2006; 441: 880–4.
12. Lattante S, de Calbiac H, Le Ber I, et al. Sqstm1 knock-down causes a locomotor phenotype ameliorated by rapamycin in a zebrafish model of ALS/FTLD. Hum Mol Genet. 2015;24:1682-90. doi: 10.1093/hmg/ddu580.
13. Lipton JO, Sahin M. The neurology of mTOR. Neuron 2014;84:275-91. doi: 10.1016/ j.neuron.2014.09.034.
14. Mantovani S, Garbelli S, Pasini A, Alimonti D, Perotti C, Melazzini M, Bendotti C, Mora G. Immune system alterations in sporadic amyotrophic lateral sclerosis patients suggest an ongoing neuroinflammatory process. J Neuroimmunol 2009;210:73-9. doi: 10.1016/j.jneuroim.2009.02.012.
15. Ravikumar B, Vacher C, Berger Z, et al. Inhibition of mTOR induces autophagy and reduces toxicity of polyglutamine expansions in fly and mouse models of Huntington disease. Nat Genet. 2004;36:585-95.
16. Shi CS, Shenderov K, Huang NN, Kabat J, et al. Activation of autophagy by inflammatory signals limits IL-1β production by targeting ubiquitinated inflammasomes for destruction. Nat Immunol 2012;13:255-63. doi: 10.1038/ni.2215.
17. Staats KA, Hernandez S, Schönefeldt S, et al. Rapamycin increases survival in ALS mice lacking mature lymphocytes. Mol Neurodegener. 2013 Sep 11;8:31. doi: 10.1186/1750-1326-8-31.

18. Thomas M, Alegre-Abarrategui J, Wade-Martins R. RNA dysfunction and aggrephagy at the centre of an amyotrophic lateral sclerosis/frontotemporal dementia disease continuum. Brain 2013;136:1345-60. doi: 10.1093/brain/awt030.
19. Wang IF, Guo BS, Liu YC, et al. Autophagy activators rescue and alleviate pathogenesis of a mouse model with proteinopathies of the TAR DNA-binding protein 43. Proc Natl Acad Sci U S A. 2012 Sep 11;109(37):15024-9. doi: 10.1073/pnas.1206362109.
20. Zhang X, Li L, Chen S, Yang D, et al. Rapamycin treatment augments motor neuron degeneration in SOD1(G93A) mouse model of amyotrophic lateral sclerosis. Autophagy2011;7:412-25.

17. APPENDIXES

17.1. ADVERSE EVENTS

17.1.1. DEFINITION OF ADVERSE EVENT

An adverse event (AE) is defined as any untoward medical occurrence in a patient or subject which does not necessarily have a causal relationship with any medical treatment. An AE can therefore be any unfavourable and unintended sign (e.g., an abnormal laboratory finding), symptom or disease temporally associated with the use of an IMP, whether or not considered causally related to the IMP. This includes all intercurrent diseases (newly diagnosed concomitant diseases or symptoms), accidents, clinically relevant deteriorations of pre-existing diseases or clinically relevant deteriorations in clinically evaluated variables (e.g., laboratory, ECG, or physical examination). An event does not have to be documented as an AE in the CRF if the following holds true:

- Untoward medical findings that occur prior to the administration of any IMP are not considered to be AEs if they occur in the scope of investigations that are performed to check the inclusion or exclusion criteria
- Deviations in clinically evaluated variables are not considered to be AEs if similar deviations were already present prior to or at the baseline visit. These values should be reported as baseline conditions, if clinically relevant, and exclusion criteria must be obeyed
- Surgeries and other invasive procedures that are planned prior to the start of the study do not have to be documented as AEs. Planned procedures will be recorded in the CRF by the investigator at the baseline visit

17.1.2. DEFINITION OF SERIOUS ADVERSE EVENT

Serious adverse events (SAEs) are a subgroup of all AEs which fulfil internationally agreed upon definitions. They require special attention by the investigator and sponsor.
A SAE is defined as any untoward medical occurrence that at any dose:
Results in death. It should be kept in mind that death itself is not an AE but rather the outcome of an event which should be described using medical terminology. Death as the description for an AE is only acceptable in the case of a sudden death when no diagnosis can be found
Is life-threatening. This refers to an event in which the patient was at risk of death at the time of the event. It does not refer to an event which hypothetically might have caused death if it had been more severe
Requires inpatient hospitalization or prolongation of existing hospitalization. This is defined as inpatient care that covers more than one calendar day, even if the duration of hospitalization is shorter than 24 hours
Results in persistent or significant disability/incapacity
Is a congenital anomaly/birth defect

Is another important medical condition. This refers to an AE that may not be immediately life-threatening or results in death or hospitalization but may jeopardize the patient's health or may require intervention to prevent one of the outcomes listed above. Based on medical and scientific judgment this should usually be considered as serious
If there is any doubt about the seriousness of an AE, the investigator should contact the study coordinator.

17.1.3. REPORTING OF ADVERSE EVENTS

Information about all adverse events, whether volunteered by the patient, discovered through questioning by the investigator, or detected through physical examination, laboratory test or other means, will be collected and recorded on the "Adverse Event Case Report Form" and followed as appropriate.
As far as possible, each AE will also be described by:
Its duration (start and end dates)
Its severity
Its relationship to the study treatment (suspected/not suspected/not assessable)
The action(s) taken
Any AE occurring by the time of study completion (within two weeks of last drug intake) must be recorded on the AE CRF page.

17.1.4. REPORTING OF SERIOUS ADVERSE EVENTS

Any SAE, whether or not considered as related to study treatment, will be reported on a SAE form and faxed to the Pharmacovigilance Unit within 24hours following the occurrence or Investigator's knowledge of this event, even if it does not appear to be treatment-related.
The investigator is also responsible for complying with the applicable requirements related to adverse events reporting.
Any SAE, including a serious clinical laboratory abnormality occurring in a patient after providing informed consent, whilst receiving study treatment and until 28 days (4 weeks) after stopping it must be reported. All serious adverse events must also be reported for the period in which the study protocol interferes with the standard medical treatment given to a patient (e.g., treatment withdrawalduring a washout period, or change in treatment to a fixed dose of concomitant medication).
Follow-up information about a previously reported SAE must also be reported within 24hours following its receipt. If the serious adverse event has not been previously documented (new occurrence) and it is thought to be related to study treatment, the pharmacovigilance unit or the coordinator of the study may contact the investigator to obtain further information. If warranted, an investigator alert may be issued, to inform all investigators involved in any study with the same drug that this serious adverse event has been reported.
The investigator must complete the "Serious Adverse Event Report Form", assess the relationship to Rapamycin and to comparator, if applicable, and fax the completed form within 24 hours to the Pharmacovigilance Unit and to the coordinator of the study. The original and the duplicate copies of the "Serious Adverse Event Report Form", and the fax confirmation sheet must be kept with the case report forms at the study site. The monitor will review and collect a copy of the "Serious Adverse Event Report Form".
Follow-up information is also to be sent, restating the date of the original report. Either a new "Serious Adverse Event Report Form" is sent (stating that this is a follow-up), or the original one

resent (with the new information highlighted and a new date provided). The follow-up should describe whether the event has resolved or continues, if and how it was treated, and whether the patient continued or discontinued his study participation. The form and fax confirmation sheet must be retained.

The telephone and telefax numbers of the contact person for pharmacovigilance will be provided to each site and a copy will be kept in the Investigator file.

According to local requirements, reporting the SAEs to the regulatory authorities, ethic committees and other investigators will be done.

17.1.5. CAUSALITY ASSESSMENT

The investigator has to assess the causal relation of the AE to the IMP. The investigator should base the assessment of a causal relationship on the following scale:

Not suspected

-There is evidently another explanation for the AE:
-The AE is obviously explained by the patient's disease(s) or
-The AE is in accordance with the effect or adverse effect of a concomitant medication or
-The AE had occurred already prior to administration of the IMP in comparable intensity and/or frequency or
-The AE started before the first intake of IMP

Suspected

-If there is a reasonable temporal relationship between the AE and the intake of the IMP, there are plausible reasons that point to a causal relationship with the IMP.
-Not assessable
-Due to conflicting medical information and/or patient status, no causal relationship can be stated.

17.1.6. INTENSITY OF ADVERSE EVENTS

Grade refers to the severity of the AE.

For oncology studies, The NCI CTCAE v4.0 (published May 2009) displays Grades 1 through 5 with clinical description of severity for each AE.

For non-oncology study, the severity of adverse event is described as follows:

-Mild adverse event: awareness of event but easily tolerated
-Moderate adverse event: discomfort enough to cause interference with usual activity
-Severe adverse event: inability to carry out usual activity.

17.1.7. ACTIONS TAKEN IN RESPONSE TO AN ADVERSE EVENT

The actions taken in response to an AE are described on a numerical scale, from 1 to 5 that covers the various possibilities. One of these has to be selected:

1 = No action taken
2 = Study treatment dosage reduced
3 = Study treatment temporary interrupted
4 = Study treatment permanently discontinued due to this adverse event

5 = Study treatment temporary interrupted and dosage reduced

17.1.8. FOLLOW-UP ON ONGOING ADVERSE EVENTS

Patients discontinuing the study with reported AEs that have not yet completely resolved must return for one or more follow-up visit(s).

-Adverse events will be followed-up until their resolution or until stabilization stated by the investigator.
-Particular attention should be given to:
-SAEs
-Ongoing non-serious AEs likely or definitely related to the IMP according to the investigator's causality assessment
-Ongoing AEs leading to the patient's premature discontinuation
-Any laboratory value or vital signs being beyond the sponsor-defined alert limit

The investigator should perform one or more follow-up visits during the first 28 days after the patient's treatment phase to examine whether the AE resolved. The AE is monitored until either normalization, return to the baseline value or identification of a permanent change. In case of minor AEs a phone call to the patient may be acceptable. If necessary, additional safety investigations and queries will be done during and after this time period.
Follow-up information on the outcome must be recorded on the respective AE page in the CRF or in the data clarification form. All efforts to achieve follow-up information must be documented in the source data. Source data information has to be available upon request.
Follow-up investigations may also be necessary according to the investigator's medical judgment even if the patient has no AE at the end of the study. However, information related to these investigations does not have to be documented in the CRF but must be recorded in the source documentation.

17.2 DECLARATION OF HELSINKI – ETHICAL PRINCIPLES FOR MEDICALRESEARCH INVOLVING HUMAN SUBJECTS

World Medical Association

Adopted by the 18th World Medical Assembly, Helsinki, Finland, June 1964
and amended by the
29th World Medical Assembly, Tokyo, Japan, October 1975,
35th World Medical Assembly, Venice, Italy, October 1983,
41st World Medical Assembly, Hong Kong, September 1989
and the
48th General Assembly, Somerset West, Republic of South Africa, October 1996
52nd WMA General Assembly, Edinburgh, Scotland, October 2000
53rdWMA General Assembly,Washington, DC, USA, October 2002 (Note of Clarification added)
55th WMA General Assembly, Tokyo, Japan, October 2004 (Note of Clarification added)

Via Giardini, 1355
Baggiovara
41126 Modena
tel.: 059396111
fax.: 0593962409

59thWMA General Assembly, Seoul, Republic of Korea, October 2008
64th WMA General Assembly, Fortaleza, Brazil, October 2013

Preamble

1. TheWorldMedical Association (WMA) has developed the Declaration of Helsinki as a statement of ethical principles for medical research involving human subjects, including research on identifiable human material and data. The Declaration is intended to be read as a whole and each of its constituent paragraphs should be applied with consideration of all other relevant paragraphs.
2. Consistent with the mandate of theWMA, the Declaration is addressed primarily to physicians. The WMA encourages others who are involved in medical research involving human subjects to adopt these principles.

General Principles

3. The Declaration of Geneva of the WMA binds the physician with the words, "The health of my patient will be my first consideration," and the International Code of Medical Ethics declares that, "A physician shall act in the patient's best interest when providing medical care."
4. It is the duty of the physician to promote and safeguard the health, well-being and rights of patients, including those who are involved in medical research. The physician's knowledge and conscience are dedicated to the fulfilment of this duty.
5. Medical progress is based on research that ultimately must include studies involving human subjects.
6. The primary purpose of medical research involving human subjects is to understand the causes, development and effects of diseases and improve preventive, diagnostic and therapeuticinterventions (methods, procedures and treatments). Even the best proven interventions must be evaluated continually through research for their safety, effectiveness, efficiency, accessibility and quality.
7. Medical research is subject to ethical standards that promote and ensure respect for all human subjects and protect their health and rights.
8. While the primary purpose of medical research is togenerate new knowledge, this goal can never take precedence over the rights and interests of individual research subjects.
9. It is the duty of physicians who are involved in medical research to protect the life, health, dignity, integrity, right to self determination, privacy, and confidentiality of personal information ofresearch subjects. The responsibility for the protection of research subjects must always rest withthe physician or other health care professionals and never with the research subjects, even though they have given consent.
10. Physicians must consider the ethical, legal and regulatory norms and standards for research involving human subjects in their own countries as well as applicable international norms and standards. No national or international ethical, legal or regulatory requirement should reduce or eliminate any of the protections for research subjects set forth in this Declaration.
11. Medical research should be conducted in a manner that minimizes possible harm to the environment.
12. Medical research involving human subjects must be conducted only by individuals with the appropriate ethics and scientific education, training and qualifications. Research on patients or healthy volunteers requires the supervision of a competent and appropriately qualified physician or other health care professional.
13. Groups that are underrepresented in medical research should be provided appropriate access to participation in research.

14. Physicians who combine medical research with medical care should involve their patients in research only to the extent that this is justified by its potential preventive, diagnostic or therapeutic value and if the physician has good reason to believe that participation in the research study will not adversely affect the health of the patients who serve as research subjects.
15. Appropriate compensation and treatment for subjects who are harmed as a result of participating in research must be ensured.

Risks, Burdens and Benefits

16. In medical practice and in medical research, most interventions involve risks and burdens. Medical research involving human subjects may only be conducted if the importance of the objective outweighs the risks and burdens to the research subjects.
17. All medical research involving human subjects must be preceded by careful assessment of predictable risks and burdens to the individuals and groups involved in the research in comparison with foreseeable benefits to them and to other individuals or groups affected by the condition under investigation. Measures to minimise the risks must be implemented. The risks must be continuously monitored, assessed and documented by the researcher.
18. Physicians may not be involved in a research study involving human subjects unless they are confident that the risks have been adequately assessed and can be satisfactorily managed. When the risks are found to outweigh the potential benefits or when there is conclusive proof of definitive outcomes, physicians must assess whether to continue, modify or immediately stop the study.

Vulnerable Groups and Individuals

19. Some groups and individuals are particularly vulnerable and may have an increased likelihood of being wronged or of incurring additional harm. All vulnerable groups and individuals should receive specifically considered protection.
20. Medical research with a vulnerable group is only justified if the research is responsive to the health needs or priorities of this group and the research cannot be carried out in a non vulnerable group. In addition, this group should stand to benefit from the knowledge, practices or interventions that result from the research.

Scientific Requirements and Research Protocols

21. Medical research involving human subjects must conform to generally accepted scientific principles, be based on a thorough knowledge of the scientific literature, other relevant sources of information, and adequate laboratory and, as appropriate, animal experimentation. The welfare of animals used for research must be respected.
22. The design and performance of each research study involving human subjects must be clearly described and justified in a research protocol. The protocol should contain a statement of the ethical considerations involved and should indicate how the principles in this Declaration have been addressed. The protocol should include information regarding funding, sponsors, institutional affiliations, potential conflicts of interest, incentives for subjects and information regarding provisions for treating and/or compensating subjects who are harmed as a consequence of participation in the research study.
In clinical trials, the protocol must also describe appropriate arrangements for post-trial provisions.

Research Ethics Committees

23. The research protocol must be submitted for consideration, comment, guidance and approval to

Via Giardini, 1355
Baggiovara
41126 Modena
tel.: 059396111
fax.: 0593962409

the concerned research ethics committee before the study begins. This committee must be

Via Giardini, 1355
Baggiovara
41126 Modena
tel.: 059396111
fax.: 0593962409

transparent in its functioning, must be independent of the researcher, the sponsor and any other undue influence and must be duly qualified. It must take into consideration the laws and regulations of the country or countries in which the research is to be performed as well as applicable international norms and standards but these must not be allowed to reduce or eliminate any of the protections for research subjects set forth in this Declaration. The committee must have the right to monitor ongoing studies. The researcher must provide monitoring information to the committee, especially information about any serious adverse events. No amendment to the protocol may be made without consideration and approval by the committee. After the end of the study, the researchers must submit a final report to the committee containing a summary of the study's findings and conclusions.

Privacy and Confidentiality

24. Every precaution must be taken to protect the privacy of research subjects and the confidentiality of their personal information.

Informed Consent

25. Participation by individuals capable of giving informed consent as subjects in medical research must be voluntary. Although it may be appropriate to consult family members or community leaders, no individual capable of giving informed consent may be enrolled in a research study unless he or she freely agrees.

26. In medical research involving human subjects capable of giving informed consent, each potential subject must be adequately informed of the aims, methods, sources of funding, any possible conflicts of interest, institutional affiliations of the researcher, the anticipated benefits and potential risks of the study and the discomfort it may entail, post-study provisions and any other relevant aspects of the study. The potential subject must be informed of the right to refuse to participate in the study or to withdraw consent to participate at any time without reprisal. Special attention should be given to the specific information needs of individual potential subjects as wellas to the methods used to deliver the information. After ensuring that the potential subject has understood the information, the physician or another appropriately qualified individual must then seek the potential subject's freely-given informed consent, preferably in writing. If the consent cannot be expressed in writing, the non-written consent must be formally documented and witnessed. All medical research subjects should be given the option of being informed about the general outcome and results of the study.

27. When seeking informed consent for participation in a research study the physician must be particularly cautious if the potential subject is in a dependent relationship with the physician or may consent under duress. In such situations the informed consent must be sought by an appropriately qualified individual who is completely independent of this relationship.

28. For a potential research subject who is incapable of giving informed consent, the physician must seek informed consent from the legally authorised representative. These individuals must not be included in a research study that has no likelihood of benefit for them unless it is intended to promote the health of the group represented by the potential subject, the research cannot instead be performed with persons capable of providing informed consent, and the research entails onlyminimal risk and minimal burden.

29. When a potential research subject who is deemed incapable of giving informed consent is able to give assent to decisions about participation in research, the physician must seek that assent in

addition to the consent of the legally authorised representative. The potential subject's dissent should be respected.

30. Research involving subjects who are physically or mentally incapable of giving consent, for example, unconscious patients, may be done only if the physical or mental condition that prevents giving informed consent is a necessary characteristic of the research group. In such circumstances the physician must seek informed consent from the legally authorised representative. If no such representative is available and if the research cannot be delayed, the study may proceed without informed consent provided that the specific reasons for involving subjects with a condition that renders them unable to give informed consent have been stated in the research protocol and the study has been approved by a research ethics committee. Consent to remain in the research must be obtained as soon as possible from the subject or a legally authorised representative.

31. The physician must fully inform the patient which aspects of their care are related to the research. The refusal of a patient to participate in a study or the patient's decision to withdraw from the study must never adversely affect the patient-physician relationship.

32. For medical research using identifiable human material or data, such as research on material or data contained in biobanks or similar repositories, physicians must seek informed consent for its collection, storage and/or reuse. There may be exceptional situations where consent would be impossible or impracticable to obtain for such research. In such situations the research may be done only after consideration and approval of a research ethics committee.

Use of Placebo

33. The benefits, risks, burdens and effectiveness of a new intervention must be tested against those of the best proven intervention(s), except in the following circumstances: Where no proven intervention exists, the use of placebo, or no intervention, is acceptable; or Where for compelling and scientifically sound methodological reasons the use of any intervention less effective than the best proven one, the use of placebo, or no intervention is necessary to determine the efficacy or safety of an intervention and the patients who receive any intervention less effective than the best proven one, placebo, or no intervention will not be subject to additional risks of serious or irreversible harm as a result of not receiving the best proven intervention. Extreme care must be taken to avoid abuse of this option.

Post-Trial Provisions

34. In advance of a clinical trial, sponsors, researchers and host country governments should make provisions for post-trial access for all participants who still need an intervention identified as beneficial in the trial. This information must also be disclosed to participants during the informed consent process.

Research Registration and Publication and Dissemination of Results

35. Every research study involving human subjects must be registered in a publicly accessible database before recruitment of the first subject.

36. Researchers, authors, sponsors, editors and publishers all have ethical obligations with regard to the publication and dissemination of the results of research. Researchers have a duty to make publicly available the results of their research on human subjects and are accountable for the completeness and accuracy of their reports. All parties should adhere to accepted guidelines for ethical reporting. Negative and inconclusive as well as positive results must be published orotherwise made publicly available. Sources of funding, institutional affiliations and conflicts of

interest must be declared in the publication. Reports of research not in accordance with the principles of this Declaration should not be accepted for publication.

Unproven Interventions in Clinical Practice

37. In the treatment of an individual patient, where proven interventions do not exist or other known interventions have been ineffective, the physician, after seeking expert advice, with informedconsent from the patient or a legally authorised representative, may use an unproven intervention if in the physician's judgement it offers hope of saving life, re-establishing health or alleviating suffering. This intervention should subsequently be made the object of research, designed to evaluate its safety and efficacy. In all cases, new information must be recorded and, where appropriate, made publicly available.

17.3 ITALIAN VERSION OF THE ALSFRS-R

1. LINGUAGGIO

Normale processo fonatorio	4
Alterazione evidenziabile del linguaggio	3
Intelligibile con ripetizioni	2
Linguaggio associato a comunicazione non vocale	1
Perdita di linguaggio utile	0

2. **SALIVAZIONE**

Normale	4
Lieve ma definito eccesso di saliva nella bocca; può avere una perdita notturna	3
Saliva moderatamente eccessiva; può avere una perdita minima	2
Marcato eccesso di saliva con una certa perdita	1
Marcata perdita; richiede costantemente l'uso di fazzoletti	0

3. **DEGLUTIZIONE**

Normali abitudini alimentari	4
Iniziali problemi alimentari – occasionalmente va per traverso	3
Modificazioni della consistenza della dieta	2
Necessita di alimentazione enterale supplementare	1
Non in grado di deglutire (alimentazione esclusivamente parenterale o enterale)	0

4 SCRIVERE A MANO (si consideri la mano dominante prima dell'esordio della SLA)

Normale	4
Rallentato o approssimato: tutte le parole sono leggibili	3

Non tutte le parole sono leggibili	2
In grado di afferrare la penna ma incapace di scrivere	1
Incapace di afferrare la penna	0

5a.TAGLIARE IL CIBO E USARE UTENSILI (pazienti senza gastrostomia)

Normale	4
Talvolta rallentato e goffo, ma non richiede aiuto	3
Può tagliare la maggior parte dei cibi, anche se in modo rallentato e goffo; è necessario un certo aiuto	2
Il cibo deve essere tagliato da altri, ma riesce ancora a portarsi il cibo alla bocca da solo	1
Deve essere nutrito	0

5b.TAGLIARE IL CIBO E USARE UTENSILI (pazienti con gastrostomia)

Normale	4
Maldestro ma in grado di eseguire tutte le manipolazioni da solo	3
Necessario un certo aiuto con dispositivi di fissaggio	2
Fornisce una minima assistenza a chi lo aiuta	1
Incapace di eseguire qualsiasi aspetto di questi compiti	0

6 VESTIRSI E IGIENE

Funzione normale	4
Bada a se stesso in modo indipendente e completo con sforzo e ridotta efficienza	3
Assistenza intermittente o metodi sostitutivi	2
Necessita di aiuto per la cura del sé	1
Dipendenza totale	0

7. GIRARSI NEL LETTO E AGGIUSTARE LE COPERTE

Normale	4
Talvolta rallentato e goffo; ma non è necessario aiuto	3
Può girarsi da solo o mettere a posto le coperte ma con grande difficoltà	2
Può iniziare il movimento, ma non girarsi o mettere a posto le coperte da solo	1
Necessita di aiuto totale	0

8. CAMMINARE

Normale	4
Iniziali difficoltà di deambulazione	3
Cammina con assistenza (qualsiasi ausilio per la deambulazione comprese ortesi per la caviglia)	2
Solo movimenti funzionali che non portano alla deambulazione	1
Nessun movimento utile degli arti inferiori	0

Via Giardini, 1355
Baggiovara
41126 Modena
tel.: 059396111
fax.: 0593962409

9. SALIRE LE SCALE

Normale	4
Rallentato	3
Lieve instabilità o fatica	2
Necessita di assistenza (compreso il mancorrente)	1
Non può farlo	0

10. DISPNEA

Nessuna	4
Dispnea quando cammina	3
Dispnea nelle attività della vita quotidiana (mangiare, lavarsi vestirsi)	2
Dispnea a riposo, difficoltà a respirare da seduti o sdraiati	1
Dispnea rilevante, considerare l'uso di supporto respiratorio meccanico	0

11. ORTOPNEA

Nessuna	4
Qualche difficoltà nel dormire la notte, non usa più di due cuscini	3
Necessità di un cuscino aggiuntivo per dormire (più di due cuscini)	2
Può dormire solo seduto	1
Non riesce a dormire	0

12. INSUFFICIENZA RESPIRATORIA

Nessuna	4
Uso intermittente di BiPAP	3
Uso continuo di BiPAP la notte	2
Uso continuo di BiPAP la notte e il giorno	1
Ventilazione meccanica invasiva mediante intubazione o tracheostomia	0

TOTALE ____/48

17.4 ITALIAN VERSION OF ALSAQ-40

Le seguenti affermazioni si riferiscono ad alcune difficoltà che lei potrebbe aver riscontrato durante le ultime due settimane. Indichi per favore, ponendo una crocetta sulla casella appropriata, come si è sentito riguardo le seguenti affermazioni.
Se lei non può compiere affatto l'azione indicata dall'affermazione, la preghiamo di segnare la casella corrispondente a: ***Sempre / Non posso farlo affatto***

Quanto spesso durante le ultime due settimane ha pensato che le seguenti affermazioni fossero vere?

Ponga per favore una crocetta nella casella corrispondente ad ogni affermazione

	Mai	Raramente	Qualche Volta	Spesso	Sempre / non posso farlo affatto
1) Ho avuto difficoltà nel camminare anche per brevi distanze, per esempio in casa	|__|	|__|	|__|	|__|	|__|
2) Sono caduto/a e mentre camminavo	|__|	|__|	|__|	|__|	|__|
3) Sono inciampato/a mentre camminavo	|__|	|__|	|__|	|__|	|__|
4) Ho perso l'equilibrio mentre camminavo	|__|	|__|	|__|	|__|	|__|
5) Ho dovuto concentrarmi nel camminare	|__|	|__|	|__|	|__|	|__|
6) Camminare mi ha stancato moltissimo	|__|	|__|	|__|	|__|	|__|
7) Ho sentito male alle gambe mentre camminavo	|__|	|__|	|__|	|__|	|__|
8) Ho avuto difficoltà nel salire e scendere le scale	|__|	|__|	|__|	|__|	|__|
9) Ho trovato difficile stare in piedi	|__|	|__|	|__|	|__|	|__|
10) Ho avuto difficoltà nell'alzarmi	|__|	|__|	|__|	|__|	|__|

dalle sedie					
11) Ho avuto difficoltà nell' usare le braccia e le mani	|__|	|__|	|__|	|__|	|__|
12) Ho avuto difficoltà nel girarmi e muovermi nel letto	|__|	|__|	|__|	|__|	|__|
13) Ho avuto difficoltà nell'afferrare gli oggetti	|__|	|__|	|__|	|__|	|__|
14) Ho avuto difficoltà nel tenere in mano libri o giornali,o girarne le pagine	|__|	|__|	|__|	|__|	|__|
15) Ho avuto difficoltà nello scrivere con chiarezza	|__|	|__|	|__|	|__|	|__|
16) Ho avuto difficoltà nel fare i lavori in casa	|__|	|__|	|__|	|__|	|__|
17) Ho avuto difficoltà nel mangiare con le posate	|__|	|__|	|__|	|__|	|__|
18) Ho avuto difficoltà nel pettinarmi o nel lavarmi i denti	|__|	|__|	|__|	|__|	|__|
19) Ho avuto difficoltà nel vestirmi	|__|	|__|	|__|	|__|	|__|
20) Ho avuto difficoltà nel lavarmi sul lavandino del bagno	|__|	|__|	|__|	|__|	|__|
21) Ho avuto difficoltà nel deglutire	|__|	|__|	|__|	|__|	|__|
22) Ho avuto difficoltà nel mangiare cibi solidi	|__|	|__|	|__|	|__|	|__|
23) Ho avuto difficoltà nel bere bevande liquide	|__|	|__|	|__|	|__|	|__|
24) Ho avuto difficoltà nel partecipare alle conversazioni	|__|	|__|	|__|	|__|	|__|
25) Credo che non sia stato facile capirmi quando parlavo	|__|	|__|	|__|	|__|	|__|

26) Ho balbettato o detto parole non comprensibili	|__|	|__|	|__|	|__|	|__|
27) Ho dovuto parlare molto lentamente	|__|	|__|	|__|	|__|	|__|
28) Ho parlato meno di quanto fossi solito/a fare	|__|	|__|	|__|	|__|	|__|
29) Mi sono sentito/a frustrato/a a causa del mio modo di parlare	|__|	|__|	|__|	|__|	|__|
30) Mi sono sentito/a a disagio a causa del mio modo di parlare	|__|	|__|	|__|	|__|	|__|
31) Mi sono sentito/a solo/a	|__|	|__|	|__|	|__|	|__|
32) Mi sono annoiato/a	|__|	|__|	|__|	|__|	|__|
33) Mi sono sentito/a imbarazzato/a in alcune situazioni sociali	|__|	|__|	|__|	|__|	|__|
34) Mi sono sentito/a scoraggiato/a per il futuro	|__|	|__|	|__|	|__|	|__|
35) Ho temuto di essere un peso per gli altri	|__|	|__|	|__|	|__|	|__|
36) Mi sono sentito/a senza motivazioni per andare avanti	|__|	|__|	|__|	|__|	|__|
37) Ho provato rabbia a causa della mia malattia	|__|	|__|	|__|	|__|	|__|
38) Mi sono sentito depresso/a	|__|	|__|	|__|	|__|	|__|
39) Mi sono preoccupato/a di come la malattia potrebbe essere nel futuro	|__|	|__|	|__|	|__|	|__|
40) Mi sono sentito/a privato/a della mia libertà	|__|	|__|	|__|	|__|	|__|

Per favore si assicuri di aver segnato una casella per ciascuna domanda.

GRAZIE DI AVER COMPILATO QUESTO QUESTIONARIO.

17.5 ITALIAN VERSION OF MRC

**Arti superiori**	Dx	Sn
Deltoide		
Bicipite brachiale		
Tricipite brachiale		
Flessione polso		
Estensione polso		
Opposizione pollice		
Flessione dita		
Estensione dita		

**Arti inferiori**	Dx	Sn
Ileopsoas		
Quadricipite		
Bicipite femorale		
Tibiale anteriore		
Gemelli		
Flessione dita		
Estensione dita		

**Collo**	
Flessione	
Estensione	

PUNTEGGI:
5/5 alla scala MRC: movimento possibile contro resistenza massima;
4/5 alla scala MRC: movimento possibile solo contro resistenza minima;
3/5 alla scala MRC: movimento possibile solo contro gravità;
2/5 alla scala MRC: movimento possibile solo in assenza di gravità;
1/5 alla scala MRC: accenno al movimento;
0/5 alla scala MRC: assenza di movimento;
