# Supplementary material for: Randomized, double-blind, placebo-controlled trial of rapamycin in amyotrophic lateral sclerosis
Source: Nat Commun. 2023 Aug 17;14:4970. doi: 10.1038/s41467-023-40734-8 (PMC10435464; doi:10.1038/s41467-023-40734-8)
Supplement: Supplementary file 3 — Reporting Summary [file 41467_2023_40734_MOESM3_ESM.pdf]

nature portfolio

Corresponding author(s): JESSICA MANDRIOLI

Last updated by author(s): Jun 6, 2023

Reporting Summary

Nature Portfolio wishes to improve the reproducibility of the work that we publish. This form provides structure for consistency and transparency in reporting. For further information on Nature Portfolio policies, see our Editorial Policies and the Editorial Policy Checklist.

Statistics

For all statistical analyses, confirm that the following items are present in the figure legend, table legend, main text, or Methods section.

n/a	Confirmed	
☐	☒	The exact sample size (*n*) for each experimental group/condition, given as a discrete number and unit of measurement
☐	☒	A statement on whether measurements were taken from distinct samples or whether the same sample was measured repeatedly
☐	☒	The statistical test(s) used AND whether they are one- or two-sided<br>*Only common tests should be described solely by name; describe more complex techniques in the Methods section.*
☐	☒	A description of all covariates tested
☐	☒	A description of any assumptions or corrections, such as tests of normality and adjustment for multiple comparisons
☐	☒	A full description of the statistical parameters including central tendency (e.g. means) or other basic estimates (e.g. regression coefficient) AND variation (e.g. standard deviation) or associated estimates of uncertainty (e.g. confidence intervals)
☐	☒	For null hypothesis testing, the test statistic (e.g. *F*, *t*, *r*) with confidence intervals, effect sizes, degrees of freedom and *P* value noted<br>*Give P values as exact values whenever suitable.*
☒	☐	For Bayesian analysis, information on the choice of priors and Markov chain Monte Carlo settings
☐	☒	For hierarchical and complex designs, identification of the appropriate level for tests and full reporting of outcomes
☐	☒	Estimates of effect sizes (e.g. Cohen's *d*, Pearson's *r*), indicating how they were calculated

Our web collection on statistics for biologists contains articles on many of the points above.

Software and code

Policy information about availability of computer code

Data collection	Pseudoanonymized data collected at each trial site were input in an in-house web based electronic case report form (CRF) written in PHP version 7.2, jquery version 3.4.1 and PostgreSQL version 9.5 as a database backend, set up and managed by the Coordinator Center in Modena (Unit of Statistical and Methodological Support to Clinical Research, AOU, Modena, Italy) and only accessible to RAP-ALS investigators through password protected access.
Data analysis	Analyses were performed using STATA software, version 15 (StataCorp. 2017. Stata Statistical Software: Release 15. College Station, TX: StataCorp LLC) and R software, version 3.6.3 (The R Foundation for Statistical Computing, Wien).<br>Attune NxT Flow Cytometer was used for cells analyses.<br>Single-cell flow cytometry data analysis was performed by FlowJo X, and Prism 6.0.

For manuscripts utilizing custom algorithms or software that are central to the research but not yet described in published literature, software must be made available to editors and reviewers. We strongly encourage code deposition in a community repository (e.g. GitHub). See the Nature Portfolio guidelines for submitting code & software for further information.

Data

Policy information about availability of data

All manuscripts must include a data availability statement. This statement should provide the following information, where applicable:

- Accession codes, unique identifiers, or web links for publicly available datasets
- A description of any restrictions on data availability
- For clinical datasets or third party data, please ensure that the statement adheres to our policy

The data that support the findings of this study are available from the corresponding author (jessica.mandrioli@unimore.it) to investigators whose proposed use of the data has been approved by an independent review committee identified for this purpose. To gain access, data requestors will need to sign a data access agreement.
However, individual participant data will not be available because informed consent did not explicitly include this.
Source data for Figures 1-5 of the main text are provided as Source Data files with this paper. The study protocol, including the statistical analysis plan has been uploaded in the Supplementary Information file.

Research involving human participants, their data, or biological material

Policy information about studies with human participants or human data. See also policy information about sex, gender (identity/presentation), and sexual orientation and race, ethnicity and racism.

Reporting on sex and gender	The findings of this trial apply to both sexes. Both sexes were considered in study design. Gender was not considered in study design. Sex was determined based on self-reporting. Aggregated sex data will be available in the source data. All enrolled subjects signed a consent regarding the use of ano nymized data. Globally, 31 men and 32 women participated in this study.
Reporting on race, ethnicity, or other socially relevant groupings	This study does not use socially constructed or socially relevant categorization variables.
Population characteristics	The following population characteristics of the trial participants were considered: age, sex, BMI and BSA, treatment categories, ALS onset (bulbar versus limb onset), time since ALS symptom onset, ALSFRS-R sxcores, Prebaseline ALSFRS-R slope, Forced vital capacity, ALSAQ40 score.
Recruitment	This randomized, double-blind, placebo-controlled trial was conducted at 7 Italian ALS referral centers from 2017 through 2020. All the participants provided written informed consent before screening. Patients were screened for inclusion and exclusion criteria by the caring neurologist at the participant ALS Centers. Inclusion and exclusion criteria had to be fulfilled also at baseline. Patients were randomly assigned to the 3 treatment arms and randomization was stratified by progression rate.<br>The study promoter was Azienda Ospedaliero Universitaria (AOU) di Modena. The trial design, data analysis, and manuscript development were shared by the Steering Committee of the Study represented by all the local PIs.
Ethics oversight	The study was approved by Comitato Etico Provinciale di Modena on 23th May 2017 (file number 95/17); AIFA (national competent authority) approved the trial on 14th July 17

Note that full information on the approval of the study protocol must also be provided in the manuscript.

Field-specific reporting

Please select the one below that is the best fit for your research. If you are not sure, read the appropriate sections before making your selection.

☒ Life sciences ☐ Behavioural & social sciences ☐ Ecological, evolutionary & environmental sciences

For a reference copy of the document with all sections, see nature.com/documents/nr-reporting-summary-flat.pdf

Life sciences study design

All studies must disclose on these points even when the disclosure is negative.

Sample size	Sample size was calculated using data from an Italian study showing that ALS patients have a decreased number of circulating Treg% (mean +SD: 2.1+0.7) if compared to healthy controls (2.6+0.6), except for slow progressors40. Considering normal values of total Treg of 71.5+17/ mmc on a normal total lymphocytes count between 1,000 and 4,500/mmc, slowly progressing ALS patients presented a number of Tregs that was equal to healthy controls, whereas fast progressors had 31% fewer Tregs40.<br>Since Treg % were demonstrated to be inversely correlated with the rate of disease progression,41 we considered a "positive response" as an increase of the proportion of Tregs by at least 30% at treatment end. The null hypothesis was that Rapamycin could not significantly increase the proportion of positive responses in treated patients at week 18, compared to baseline and to placebo group. The alternative hypothesis was that Rapamycin could determine a positive response in at least 50% of treated patients compared to a maximum 5% of patients in the placebo group. The study was designed to reject the null hypothesis with an alpha error of 0.025 (in order to take into account multiple arms comparisons) and a power of 0.80.<br>With a 1:1:1 randomization ratio among the three arms we calculated that 54 participants would provide 80% power to detect a 30%

difference in the percentual of circulating Treg in at least 50% of treated patients versus less than 5% in the placebo group, using a chi-square test without any correction at a two-sided alpha level of 0.025.
The study was planned to reach a sample size of 63 patients taking into consideration a possible 15% of drop out. Safety analyses were performed

Data exclusions

We did not exclude data from analyses.

Replication

To ensure replication of results we used the gold standard design to assess drug effects in human subject, which is the randomized controlled design. Furthermore a protocol was shared with all participating center to ensure adherence to study design, training on clinical outcome measures, and shared procedures for plasma drug assessment. All outcome measures were collected at fixed time points as per clinical protocol (at baseline, week 8, week 18, week 30, and week 54).
As far as biological outcome measures, the quantification of the percentages of different cell populations were performed once for each patient and independently. This was allowed because of regular calibration of the Attune™ NxT flow cytometer was performed each day and ensured by "Performance Tracking Beads", (ThermoFisher, Catalog number: 4449754). This performance test ensure the accuracy and sensitivity of the instrument and allow easy identification of potential shifts or trends in instrument performance. Moreover, single stained controls were performed everyday and compensation matrix calculated accordingly. This method is largely used by our group and was well described in three recent publications in Nature Communications (De Biasi et al., Nat Commun 2020, PMID: 32632085; De Biasi et al., Nat Commun. 2021, PMID: 33723257: De Biasi et al., Nat Commun. 2021, PMID: 34326336).

Randomization

Eligible participants were randomly assigned in three treatment arms with a 1:1:1 ratio to receive rapamycin 1 mg per square meter (m2) of body surface area (BSA) a day (1 mg/m2/day) (21 patients), rapamycin 2 mg/m2/day (21 patients), or placebo (21 patients). Computerized randomization was stratified by rate of disease progression as measured by monthly decline of the Revised ALS Functional Rating Scale (ALSFRS-R) from onset to screening visit, with a cut off set at 0.7

Blinding

This was a randomized, doble.blind, placebo controlled clinical trial. Euromed Clinical Supply Services (ECLISSE) (Cantù, Como, Italy; http://www.css.euromed.it/en/) prepared the active formulation and the placebo complying the Good Manufacturing Practices of the European Union for active pharmaceutical ingredients and ICH Q7A guidelines. Treatment under investigation and placebo were made indistinguishable to patients and neurologists.

Reporting for specific materials, systems and methods

We require information from authors about some types of materials, experimental systems and methods used in many studies. Here, indicate whether each material, system or method listed is relevant to your study. If you are not sure if a list item applies to your research, read the appropriate section before selecting a response.

Materials & experimental systems

n/a	Involved in the study	
☐	☒	Antibodies
☒	☐	Eukaryotic cell lines
☒	☐	Palaeontology and archaeology
☒	☐	Animals and other organisms
☐	☒	Clinical data
☒	☐	Dual use research of concern
☒	☐	Plants

Methods

n/a	Involved in the study	
☒	☐	ChIP-seq
☐	☒	Flow cytometry
☒	☐	MRI-based neuroimaging

Antibodies

Antibodies used

Freshlysolated PBMC were washed and stained with commercially available monoclonal antibodies (mAb) directly conjugated with different fluorochromes. To fully dissect monocytes, NK cells and T cells we used three different flow cytometry panels. For monocyte phenotype: beside viability marker (AQUA, Live Dead, ThermoFisher, Eugene, OR) anti- CD14-APC, -CD16-AF488, HLA-DR-Pe-Cy7, and chemokine receptors CCR2-BV421, CXCR4-PE, CCR5-BV605 (all from Biolegend, San Diego, CA).
Classical monocytes were defined as CD14+, CD16+, intermediate monocytes were defined as CD14+, CD16bright, non-classical monocytes were defined as CD14-CD16bright. Median fluorescent intensity (MFI) of chemokine receptors was measured in all monocyte subpopulations.
For the immunophenotype of NK cells the following markers were used, beside viability marker (AQUA Live Dead, ThermoFisher, Eugene, OR): anti-CD16-BV421, -CD56-PE-Cy7, -CD158a-PE, -CD158b-FITC, HLA-DR-AF700, CD62L-BV605, CD57-APC, -CD3-PE-Dazzle-594, -CD4-PE-Dazzle-594-PE-Dazzle-594, -CD14-PE-Dazzle-594, -CD19-PE-Dazzle-594.
For T regulatory cells and T cells phenotype the following markers, beside viability marker (AQUA Live Dead) were used: anti-CD3-Pe-Cy5, -CD4-AF700, CD8-APC-Cy7, CD25-PE, CD127-APC-Cy7, HLA-DR-PE-Cy7, CXCR3-BV421, CD38-BV605, PD-1-BV605, CD39-BV421.
Cells were fixed and perm using Human FoxP3 Buffer Set (BD Bioscience, San José, CA) and the stained with anti-FoxP3-PE (BD).
Finally, functional analysis on freshly isolated PBMC after in vitro stimulation for 16 hours with anti-CD3 plus -CD28 (1ug/ml, each, Miltenyi, Germany) was performed to evaluate the metabolic mTOR function by the evaluation of the phosphorylation of S6 Ribosomal Protein, a protein phosphorylated by mTOR. Cells were stained with viability marker (AQUA Live Dead, ThermoFisher), anti- CD3-PE-Cy5, -CD4-AF700, -CD8-APC-Cy7, -CD127-APC-Cy7, -CCR7-FITC, -CD45RA-PE-Cy7, -CD25-BV605. Cells were washed and then fixed and perm with Intracellular Fixation & Permeabilization Buffer Set (eBioscience, San Diego, CA, USA). Finally, cells were stained with PE-conjugated S6 Ribosomal Protein (Ser235/Ser236) (eBioscience, San Diego, CA, USA) to evaluate the activation of mTOR. Cells were acquired on Attune NxT acoustic flow cytometer and a minimum of 500 000 cells was acquired.
B-cell immunophenotype was perfomed by using DuraClone IM B tubes (Beckman Coulter, Hialeah, FL). Thawed PBMC were stained

with viability marker Promokine IR-840 (PromoCell Gmb H, Heidelberg, Germany) for 20 min at room temperature in PBS. One million PBMC were washed with FACSbuffer and stained with DuraClone IM B cells containing the following lyophilized directly conjugated mAbs: IgD-FITC, CD21-PE, CD19-ECD, CD27-PC7, CD24-APC, CD38-AF750, IgM-PB, CD45-KrO. Cells were washed with FACS buffer and acquired at CytoflexLX flow cytometer (Beckman Coulter, Hialeah, FL). A minimum of 500 000 cells was acquired on a CytoFLEX LX flow cytometer (Beckman Coulter) according to the state-of-the-art methodology.

Marker Fluorochrome Clone Company Cat Number Titer (ul)

Live Dead Fixable AQUA ThermoFisher L34957 1.25
Live Dead Fixable RED ThermoFisher L34972 0.3
CD3 PE-dazzle 594 UCHT1 Biolegend 300450 0.3
CD4 PE-dazzle 594 RPTA-4 Biolegend 300548 0.3
CD14 PE-dazzle 594 M14-23 Biolegend 325634 0.3
CD19 PE-dazzle 594 HIB19 Biolegend 302252 0.3
CD16 BV421 3G8 Biolegend 302038 0.3
CD56 PE-CY7 MEM-188 Biolegend 304628 0.3
CD8 APC-CY7 RPTA-8 Biolegend 301016 0.3
CD62L BV603 DREG-56 Biolegend 304834 0.3
CD158A PE HP-MA4 Biolegend 339506 0.3
CD158B FITC DX27 Biolegend 312604 0.3
HLA-DR AF700 LN3 Biolegend 327014 0.3
CD57 APC HNK-1 Biolegend 359614 0.3
CD3 PE-CY5 UCHT1 Biolegend 300410 0.6
CD4 AF700 RPTA-4 Biolegend 300526 0.6
CD8 APC-CY7 RPTA-8 Biolegend 301016 0.6
CD127 APC A019D5 Biolegend 351316 0.6
CD25 PE BC96 Biolegend 302606 3.75
FOXP3 FITC 206D Biolegend 320106 20
CD38 BV605 HIT2 Biolegend 303532 1.25
HLA-DR PE-CY7 L243 Biolegend 307616 0.6
CXCR3 BV421 G025H7 Biolegend 353716 0.6
PD1 BV605 EH12.2H7 Biolegend 329924 2.5
CD39 PE-CY7 A1 Biolegend 328212 0.6
CCR7 BV421 G043H7 Biolegend 353208 0.6
CD45RA PE HI100 Biolegend 304108 1.25
CD25 BV603 BC96 Biolegend 302632 1.25
Phospho-S6 (Ser235, Ser236) PE-CY7 cupk43k eBioscience 25-9007-42 3.75
Phospho-S6 (Ser235, Ser236) PE cupk43k eBioscience 12-9007-42 5
CD16 AF488 3G8 Biolegend 302019 0.3
CD14 APC M14-23 Biolegend 367118 0.3
HLA-DR PE-CY7 LN3 Biolegend 307616 0.3
CCR2 BV605 K036C2 Biolegend 357214 0.3
CXCR4 PE 12G5 Biolegend 306506 0.3
CCR5 BV421 J418F1 Biolegend 359118 0.3
DURACLONE IMB Beckman Coulter B53318
ViaKrome 808 808 Beckman Coulter C36628 0.3

Validation

We used only monoclonal antibodies that are commercially available and have been validated by different companies. We have titrated each of them for the optimal use by flow cytometry, as recommended by the most recent guidelines for the use of cytometry in immunological studies (Cossarizza et al., Eur. J. Immunol. 49:1457-1973, 2019).

Clinical data

Policy information about clinical studies
All manuscripts should comply with the ICMJE guidelines for publication of clinical research and a completed CONSORT checklist must be included with all submissions.

Clinical trial registration

https://www.clinicaltrialsregister.eu/ctr-search/trial/2016-002399-28/IT; ClinicalTrials.gov, NCT03359538

Study protocol

The full trial protocol is available and attached to submitted article as supplementary material

Data collection

From 05/10/2017 to 02/01/2020 a total of 70 persons with ALS were screened for eligibility, of whom 63 were randomly assigned to a trial group: 21 to rapamycin 2 mg/m2/day, 21 to rapamycin 1 mg/m2/day and 21 to placebo. Post-hoc analysis of survival was performed with last observation set on 31st December 2021. The last data was collected on 15th February 2022. Data were colected through an online CRF in 7 Italian ALS Centers located in Modena, Padua, Milan, Turin, Novara, Genoa. Immunological analyses were performed in the Laboratory of Immunoloy of Prof. Cossarizza, in Modena.

Outcomes

The primary efficacy outcome was the proportion of positive response (Tregs number increase of at least 30%) at treatment end (18 weeks) with respect to baseline, in patients treated with rapamycin compared to the placebo arm.
This difference was established based on a previous study demonstrating that slowly progressing ALS patients presented a number of Tregs that was equal to healthy controls, whereas fast progressors had 31% fewer Tregs. Since Tregs % were demonstrated to be inversely correlated with the rate of disease progression, we considered a “positive response” as an increase of the proportion of

Tregs by at least 30% at treatment end.
Secondary outcomes were:

I. Assessment of rapamycin safety and tolerability through documentation of the occurrence of any AEs, changes on clinical examination including vital signs and weight, and laboratory examinations (biochemistry, hematology and urinalysis) that were registered throughout the study duration. Symptoms consistent with disease progression, were recorded as AEs.
II. Biological outcomes, assessed as the change from baseline to week 8, 18, 30, 54, comparing rapamycin and placebo arms, of the following biological variables: a) activation and homing capabilities of different T, B, NK cell subpopulations; b) relative expression of inflammasome genes and its activation status; c) phosphorylation of the S6 ribosomal protein (S6RP); d) plasma/CSF neurofilament heavy/light chain protein; e) creatinine and albumin, CK, vitamin D; f) the assessment of rapamycin in CSF samples at week 18 by LC-MS/MS.
Laboratory methods are explained in Supplementary Appendix, Section 3.3.
III. Clinical outcomes through comparison between placebo and treatment arms of: a) the changes in ALSFRS-R from baseline to weeks 4,8,12,18,30,42,54; b) overall survival from randomization to date of documented death or tracheostomy; c) survival rate at weeks 18, 30, 42, and 54; d) respiratory muscle function as assessed by FVC score from baseline to weeks 4,8,12,18,30,42,54 (Supplementary Appendix, Section 3.4).
IV. Quality of life, measured through absolute and relative change from baseline in ALSAQ-40 at week 4,8,12,18,30,42 and 54 comparing treatment and placebo arms.

Flow Cytometry

Plots

Confirm that:

☒ The axis labels state the marker and fluorochrome used (e.g. CD4-FITC).

☒ The axis scales are clearly visible. Include numbers along axes only for bottom left plot of group (a 'group' is an analysis of identical markers).

☒ All plots are contour plots with outliers or pseudocolor plots.

☒ A numerical value for number of cells or percentage (with statistics) is provided.

Methodology

Sample preparation

Blood samples (up to 30 mL) were obtained after informed consent. Peripheral blood mononuclear cells (PBMC) were isolated according to standard procedures and stored in liquid nitrogen until use. Plasma was collected and stored at -80°C until use. Measurements were taken from individual patients; in the case of plasma, each measurement was performed in duplicate and only the mean was considered and shown.

Instrument

Attune NxT acoustic flow Cytometer (ThermoF isher)

Software

FlowJo X, Prism 6.0, Attune NxT software

Cell population abundance

Aliquots of up to 5 million cells were thawed immediately before the experiment, and were used only if cell viability was >90%.

Gating strategy

Treg cells quantification. Lymphocytes have been identified according to physical parameters (FSC-H and SSC-H), doublets were removed according to FSC-A vs FSC-H plot, living CD3+ T cells were selected and in this population those cells expressing CD4 were selected. Treg cells were identified as FoxP3+, CD25++, CD127- cells within CD4+ T cells.
T cell Activation panel.
Lymphocytes have been identified according to physical parameters (FSC-H and SSC-H), doublets were removed according to FSC-A vs FSC-H plot, living CD3+ T cells were selected and in this population those cells expressing CD4 and CD8 were selected. Within CD4+ T cells and CD8+ T cells the expression of CD38, HLA-DR and CXCR3 was analysed. Activated cells were defined those expressing both CD38 and HLA-DR. Cells expressing CXCR3, a homing receptor, were also analyzed. Activation status and homing properties have been investigated also in Treg cells.
T cell Metabolic/Exhaustion panel. Lymphocytes have been identified according to physical parameters (FSC-H and SSC-H), doublets were removed according to FSC-A vs FSC-H plot, living CD3+ T cells were selected and in this population those cells expressing CD4 and CD8 were selected. Within CD4+ T cells and CD8+ T cells the expression of CD39, and PD1 was analysed. The expression of these molecules has been investigated also in Treg cells.
Gating strategy of NK cells. NK cells were identified according to physical parameters, i.e., FSC-H and SSC-H. Then, we excluded cell doublets from the analysis, and we identified live cells. Dump channel containing anti-CD19, -CD4, -CD3, -CD14 was used to get rid of all unwanted cells. In this negative population, cells expressing CD16 and CD56 has been defined as NK. In particular, three populations of NK cells have been identified according to the expression of CD56 (negative, dim and bright). In the main represented population (CD16+, CD56 dim), the expression of CD57, CD62L, HLA-DR, CD158, CD158B have been investigated.
T cell differentiation status and pS6 quantification in CD4+, CD8+ T cells and Treg cells. Lymphocytes have been identified according to physical parameters (FSC-H and SSC-H), doublets were removed according to FSC-A vs FSC-H plot, living CD3+ T cells were selected and in this population those cells expressing CD4 and CD8 were selected. Within CD4+ T cells and CD8+ T cells the expression of CD45RA, CCR7 and pS6 was analysed. The expression of these molecules has been investigated also in Treg cells. Naive T cells (N): CD45RA+CCR7+; effector memory T cells (EM): CD45RA-, CCR7; central memory T cells (CM) CD45RA-,CCR7; terminal differentiated effector memory T cells (EMRA): CCR4-,CD45RA+.
Gating strategy used for monocytes. Monocytes were identified according to physical parameters, i.e., forward scatter-height (FSC-H) and side scatter-height (SSC-H). Then, we excluded cell doublets from the analysis, and we identified alive monocytes

that express HLA-DR. Finally, we recognized monocyte subpopulations on the basis of CD14 and CD16 expression: classical (CD14++, CD16-), intermediate (CD14++, CD16+), and non-classical (CD14+, CD16+) monocytes. The median fluorescence intensity (MFI) value for the three membrane receptors CCR2, CCR5, and CXCR4 was evaluated in the different monocyte subsets.
Gating strategy used to identify B cells subpopulations. A first gate was set in IgD and time, then on CD45+ cells, on physical parameters (FSC-H vs FSC-W) to eliminate doublets, then on CD19 and promokine to identify alive B cells. Naïve, exhausted and IgD-IgM expressing B cells were evaluated on B cells gate. Switched memory and unswitched memory were evaluated on IgD- IgM- and IgD+ IgM+ gate, respectively. IgM-only memory B cells were evaluated on IgD- IgM+. Finally, transitional B cells were evaluated among CD38dim, CD38high gate.
Gating strategy used to investigate the expression of LAG-3, IL-10, CD49b and p38 MAPK in Treg cells
Treg cells have been identified as CD127-, FoxP3+ cells within alive CD3+, CD4+ T cells. In this population, Treg cells expressing of IL-10, LAG-3, CD49b have been analyzed. Median Fluorescence Intensity (MFI) of p38 MAPK has been quantified.
T helper differentiation
Using the frozen PBMC from the same patients analyzed in the previous experiment (section 3.3.3), CD4+ T cells and CD8+ T cells subpopulations have been investigated in terms of T helper differentiation, by analyzing the expression of CXCR3, TBET, CCR4, CCR6 and CD161. In particular, CXCR3+, TBET+ has been defined as Th1, CCR4+ as Th2 and CCR6+,CD161+ as putative mucosal associated invariant T cells (MAIT) or Th17. Moreover, CCR4+,CCR6+ has been defined as Th2/Th17.

☒ Tick this box to confirm that a figure exemplifying the gating strategy is provided in the Supplementary Information.
